# Transcriptomics and miRNomics data integration in lymphoblastoid cells highlights the key role of immune-related functions in lithium treatment response in Bipolar disorder

**DOI:** 10.1186/s12888-022-04286-3

**Published:** 2022-10-27

**Authors:** Nadia Cattane, Cindie Courtin, Elisa Mombelli, Carlo Maj, Cristina Mora, Bruno Etain, Frank Bellivier, Cynthia Marie-Claire, Annamaria Cattaneo

**Affiliations:** 1 Biological Psychiatry Unit, IRCCS Istituto Centro San Giovanni di Dio Fatebenefratelli, Brescia, Italy; 2 Université Paris Cité, INSERM UMR-S 1144, Optimisation Thérapeutique en Neurospsychopharmacologie, OTeN, F-75006 Paris, France; 3 Institute for Genomic Statistics and Bioinformatics, University Hospital, Bonn, Germany; 4 Département de Psychiatrie et de Médecine Addictologique, Hôpitaux Lariboisière-Fernand Widal, GHU APHP Nord_Université Paris Cité, F-75010 Paris, France; 5 Fondation FondaMental, Créteil, France; 6 Department of Pharmacological and Biomolecular Sciences, University of Milan, Milan, Italy

**Keywords:** Bipolar disorder, Lymphoblastoid cell line, Lithium, Gene expression, microRNAs, Transcriptome and miRNome integrative analysis

## Abstract

**Background:**

Bipolar Disorder (BD) is a complex mental disease characterized by recurrent episodes of mania and depression. Lithium (Li) represents the mainstay of BD pharmacotherapy, despite the narrow therapeutic index and the high variability in treatment response. However, although several studies have been conducted, the molecular mechanisms underlying Li therapeutic effects remain unclear.

**Methods:**

In order to identify molecular signatures and biological pathways associated with Li treatment response, we conducted transcriptome and miRNome microarray analyses on lymphoblastoid cell lines (LCLs) from 20 patients diagnosed with BD classified as Li responders (*n* = 11) or non-responders (*n* = 9).

**Results:**

We found 335 mRNAs and 77 microRNAs (miRNAs) significantly modulated in BD responders versus non-responders. Interestingly, pathway and network analyses on these differentially expressed molecules suggested a modulatory effect of Li on several immune-related functions. Indeed, among the functional molecular nodes, we found NF-κB and TNF. Moreover, networks related to these molecules resulted overall inhibited in BD responder patients, suggesting anti-inflammatory properties of Li.

From the integrative analysis between transcriptomics and miRNomics data carried out using miRComb R package on the same samples from patients diagnosed with BD, we found 97 significantly and negatively correlated mRNA-miRNA pairs, mainly involved in inflammatory/immune response.

**Conclusions:**

Our results highlight that Li exerts modulatory effects on immune-related functions and that epigenetic mechanisms, especially miRNAs, can influence the modulation of different genes and pathways involved in Li response. Moreover, our data suggest the potentiality to integrate data coming from different high-throughput approaches as a tool to prioritize genes and pathways.

**Supplementary Information:**

The online version contains supplementary material available at 10.1186/s12888-022-04286-3.

## Introduction

Bipolar disorder (BD) is a severe and disabling psychiatric condition characterized by intermitting states of mania and depression, affecting 1–3% of the population worldwide. The onset of the illness is around 20 years of age and it is associated with a reduced quality of life, substantial societal costs and the highest suicide rate among psychiatric disorders [1–3]. Management of *patients diagnosed with BD* requires both acute treatment of manic or hypomanic episodes above to a maintenance therapy to prevent relapses and further episodes.

Mood stabilizers are used as the first-line therapy in the treatment of BD, and among this class of compounds, lithium (Li), introduced by John Cade in 1949, still represents the gold standard treatment for stabilization, prophylaxis and suicide prevention [4, 5]. However, *patients diagnosed with BD* receiving Li need regular monitoring due to Li narrow therapeutic index and the risk to develop multiple side effects [5, 6]. Further complexity in the management of Li therapy is represented by the heterogeneity in Li response: approximately 30% of patients appear good responders, whereas 70% of them are classified as partial or non-responders [7–9].

A growing body of evidence suggests that the response to Li prophylaxis has a strong genetic background and familiar heritability [10–16]. Recent findings from the largest genome-wide association study (GWAS) conducted by the International Consortium on Lithium Genetics (ConLiGen) have indeed suggested the involvement of two long non-coding RNA (lncRNA) genes, AL157359.3 and AL157359.4, in Li response [7].

However, so far, findings from pharmacogenetic studies have been able to explain only a small proportion of the observed variability, suggesting that other factors could be involved, including for example epigenetic mechanisms, such as DNA methylation, histone modification and microRNAs (miRNAs). MiRNAs are single-stranded, non-coding RNA molecules, 18–25 nucleotides-long, with a key role in the regulation of messenger RNA (mRNA). Indeed, they act by inducing either degradation or translational silencing of their target mRNA, but in certain instances, miRNAs may activate translation or even act at the level of transcription by binding to specific gene promoters [17–20]. MiRNAs are involved in key processes such as neurogenesis [21], neural plasticity and higher brain functioning [22] and they have been also associated with neurodegenerative disorders [23, 24] and psychiatric illnesses [25]. Interestingly, although still limited, available data on miRNAs expression support the hypothesis that these small non-coding RNAs could influence the treatment response in patients diagnosed with BD [8, 26, 27].

Among human cellular models, lymphoblastoid cell lines (LCLs) represent a valid and useful experimental tool to study Li response in patients diagnosed with BD, including epigenetic mechanisms, as already demonstrated [28–34]. Despite some limitations associated with the Epstein-Barr Virus (EBV) transformation, LCLs show several advantages [35]. Indeed, the genomes of LCLs remain stable during subsequent cell divisions. This stability in part reflects the fact that the EBV genome is not incorporated into the germ-line genome but rather remains in the cell cytosol [35]. Interestingly, although some of the epigenomic signatures are lost during the EBV immortalization process and the subsequent in vitro propagation, many of them persist and may be useful to study disease-related epigenomic modifications [36–42].

Although Li treatment response in patients diagnosed with BD has been studying for decades, findings from already available studies have been elusive mainly due to: i) the complexity of the mechanism of action of Li [43, 44], ii) the involvement of multiple molecular processes [45, 46], and iii) the use of different animal models or in vitro human cell lines (for a review see [8, 34]). Moreover, the heterogeneity among all studies (in terms of analyzed samples, technologies and methods used, inclusion/exclusion criteria of patients’ selection, sample size), the lack of independent replication cohorts to validate initial findings and of standard operative procedures among different laboratories (i.e. methods of collecting, storing, processing and analyzing markers), as well as the high-throughput approaches across different studies have produced big data sets, contributing to make the picture even more complex to understand.

To overcome all these limitations and to reduce heterogeneity among the studies, new successful strategies have been introduced by combining modalities coming from different high-throughput methods, which can be useful to prioritize a stringent number of genes and pathways involved in Li treatment response. For example, Hunsberger and colleagues have conducted an integrative approach combining miRNAs and mRNAs expression profiling of LCLs derived from BD Li responder and non-responder patients before and after in vitro Li treatment [30]. Interestingly, the results of this study suggested that in vitro Li treatment down-regulated miRNA Let-7 family in both groups, but specifically in the group of BD responders [30]. However, these interesting findings have not been replicated by further studies [47]. Another example of high-throughput data integration has been proposed by Pisanu and colleagues who applied a convergent analysis of genome-wide genotyping and transcriptomic data from LCLs of patients diagnosed with BD identifying a zinc finger gene, ZNF493, as a potential Li-responsive target [48].

Within this scenario, in the present study, we aimed to investigate the effects of Li treatment response in LCLs obtained from patients diagnosed with BD characterized for Li response. Thus, we performed an integrative analysis on both mRNAs and miRNAs microarray data by combining differential expression analyses and correlation of expression levels with miRComb, an R package able to combine miRNA and mRNA expression data with hybridization information [49]. Our goal was to identify molecular signatures and biological pathways that could underlie the effects of Li therapeutic response. This could have a clinical utility helping clinicians to develop a personalized medicine approach. Moreover, we aimed to find biological signatures (mRNAs, miRNAs) involved in Li response not only to better understand the molecular mechanisms associated with Li therapeutic treatment, but also to identify novel targets for the development of new drugs.

## Materials and methods

### Patients

Twenty patients with a diagnosis of Bipolar Disorder type I were recruited at the Expert Center for bipolar disorder of Paris (France) as part of a research protocol (Clinical Trials Number NCT02627404) approved by the ethical committee (Comité de Protection des Personnes – La Pitié- Salpétrière Hospital – Paris – France) (reference: P111002-IDRCB2008-AO1465–50). All participants provided written informed consent prior to inclusion.

Diagnosis was made according to the Diagnostic and Statistical Manual of Mental Disorders (DSM-IV Edition), and the Li response was evaluated by using the Retrospective Criteria of Long-Term Treatment Response in Research Subjects with BD score, also known as ALDA scale.

Briefly, the ALDA scale is composed of two subscales: the A scale and the B scale. The A scale evaluates the improvement of symptoms during the treatment with Li, while the B scale describes five clinical factors that may potentially confound the pharmacological response.

The ALDA score assumes an integer value between 0 and 10 calculated by subtracting the B from the A score. Subjects with ALDA score ≥ 7 are classified as “responders”, whereas those with ALDA score < 7 represent “non-responders” [50]. Accordingly, in our study *patients diagnosed with BD* were defined as Li responders (R, *n* = 11) or non-responders (NR, *n* = 9).

Demographical information, details of the clinical characterization and assessment of Li response of all patients diagnosed with BD have been reported in Table 1.

**Table 1:** Demographical and clinical information of BD responder (R) and non-responder (NR) patients

	R (***n*** = 11)	NR (***n*** = 9)
Age (mean ± SD)	49.06 ± 12.51	45.81 ± 6.62
Sex (M/F)	6/5	6/3
Ethnicity	Caucasian	Caucasian
Smoking (yes/no)	6/5	5/4
Age of onset (mean ± SD)	27.91 ± 9.78	29.67 ± 10.22
N° of lifetime mood episodes (mean ± SD)	7.09 ± 4.89	11.29 ± 5.79
ALDA total score (mean ± SD)	8.27 ± 1.01	1,89 ± 1.27
CTQ total score (mean ± SD)	38.55 ± 7.54	41.56 ± 10.70
Medication (yes/no) - Lithium	9/2	5/4
- Atypical antipsychotics	1/10	6/3
- Anticonvulsants	2/9	4/5

### Lymphoblastoid cell lines

Lymphoblastoid cell lines (LCLs) from *patients diagnosed with BD* were established from fresh blood by transforming lymphocytes with Epstein-Barr virus (EBV) following standard protocols [28, 51]. When reaching the confluence state, each cell line was stored in liquid nitrogen until use. For the present study, LCLs were thawed and cultured in RPMI-1640 medium supplemented with 10% foetal bovine serum, 1% penicillin/streptomycin, 1% L-Glutamine 200 mM (Thermo Fisher Scientific, USA) in a 5% CO_2_ humidified incubator at 37 °C. LCLs were seeded at 2 × 10^5^ cells/ml and grown in T75 Flask. After 4 days, cells were harvested for RNA isolation.

All the cells were tested free of mycoplasma by using TransDetect® PCR Mycoplasma Detection Kit (Clinisciences, France).

### RNA extraction

From each cell line, total RNA, including miRNAs, was isolated from 5 × 10^6^ cell pellets with the miRNeasy mini kit according to the manufacturer’s protocol (QIAGEN, Hilden, Germany). RNA quantity and quality were determined by using a NanoDrop-1000 spectrophotometer (NanoDrop Technologies, Thermo Fisher Scientific, USA). RNA purity was considered adequate when the A260/280 ratio was in the range of 1.8–2.0 and when the A260/230 ratio was in the range of 2.0–2.2. RNA integrity number, assessed using the Agilent 2100 Bioanalyzer (Agilent, Santa Clara, CA, USA), was in the range of 7–10.

### Microarrays

#### Transcriptome analysis

For the whole transcriptomic profiling, 250 ng of total RNA were processed with the WT PLUS Reagent Kit (Thermo Fisher Scientific, USA) and were subsequently hybridized onto the GeneChip Human Gene 2.1 ST Array Strips on a GeneAtlas platform (Thermo Fisher Scientific, USA). The comprehensive coverage of these array strips allows the simultaneously evaluation of > 30.000 coding transcripts, of > 11.000 long intergenic non-coding transcripts and alternative splicing events/transcript variants with probes designed to maximize exon coverage. Samples have been randomized and stratified in a way that each array strip included the same number of Li responder and Li non-responder patients diagnosed with BD. Washing and staining were conducted on the Fluidics station; scanning was performed on the Imaging station. All procedures were run following the manufacturer’s instructions.

#### miRNome analysis

250 ng of total RNA including miRNAs were processed with the FlashTag Biotin HSR RNA Labeling kit (Thermo Fisher Scientific, USA) and subsequently hybridized onto the GeneChip miRNA 4.1 Array Strip on a GeneAtlas platform (Thermo Fisher Scientific, USA). As reported before, samples have been randomized and stratified in a way that each array strip included the same number of Li responder and Li non-responder patients diagnosed with BD. Washing and staining were conducted on the Fluidics station, whereas scanning was performed on the Imaging station. All procedures were run following the manufacturer’s instructions.

### Statistics

#### Microarray data analysis

Strip array data quality control was checked by GeneAtlas integrated analysis software (Thermo Fisher Scientific, USA) for both GeneChip Human Gene 2.1 ST Array Strips and GeneChip miRNA 4.1 Array Strips.

Briefly, raw microarray data (.CEL files) were imported and analyzed with the commercially available software Partek® Genomic Suite® (Partek, St. Louis, MO, USA). Probe set normalization and summarization were computed using Robust Multi-Array (RMA) algorithm. We checked for quality control and batch effects using the Principal Component Analyses (PCA), and we did not observe any outliers or batch effect.

Analysis of variance (ANOVA) was performed to assess, separately, the expression levels of both mRNAs and miRNAs in LCLs obtained from BD responders versus non-responders.

To identify the list of differentially expressed mRNAs and miRNAs, we filtered ANOVA results using both fold change |FC| ≥ 1.2 and *p*-value ≤0.05. Moreover, in the list of miRNAs we selected the probe sets only for the Human organism.

#### Pathway analysis of differentially expressed mRNAs

Differentially expressed genes with RefSeq annotations were analyzed using the Core Analysis functionality in Ingenuity Pathway Analysis (IPA) software (QIAGEN, Hilden, Germany) to describe key aspects of the molecular function, biological process and cellular component of gene products. This software is based on a proprietary database able to identify specific cellular pathways and to generate biological networks.

#### Pathway analysis of differentially expressed mature miRNAs

The identification of potentially altered molecular pathways as targeted by differentially modulated miRNAs was performed by using DIANA-miRPath (v.3) tool web-server [52]. Specifically, an in silico target prediction was performed on differentially expressed mature miRNAs by using DIANA-microT-CDS algorithm, which combines the analysis of 3′-untranslated regions (3′-UTRs) and coding sequence regions [53]. MiRNA-gene interactions were filtered using the default threshold equal to 0.8 and the False Discovering Rate (FDR) algorithm (FDR cut-off = 0.05) was applied.

An enrichment analysis of miRNA target genes was executed in Kyoto Encyclopedia of Genes and Genomes (KEGG) database and a list of significant pathways (*p*-value ≤0.05) showing all targeted genes and number of miRNAs was computed [52].

#### mRNAs-miRNAs combined analysis

In order to detect the relevant pairs of interacting mRNA-miRNA for Li response, an integrated analysis of mRNAs and miRNAs expression levels was performed. First, differentially expressed mRNAs and miRNAs between BD responders and non-responders were filtered to focus on gene expression regulation mechanisms associated with Li response. Among all identified mRNAs and miRNAs, a correlation analysis was performed to detect mRNA-miRNA pairs that were inversely correlated based on the biological assumption that a miRNA negatively regulates the expression of its mRNA targets. The analysis has been performed using miRComb, an R package able to combine miRNA and mRNA expression data with hybridization information [49].

We first used LIMMA package to perform differential analysis and to filter for differentially expressed mRNAs and miRNAs (|FC| ≥ 1.2 and nominal *p*-value ≤0.05). The intensity signals of microarray (after RMA normalization) among all the couples of identified differentially expressed mRNAs and miRNAs have been tested for association with Pearson correlation (that is to test for the presence of an inverse linear relationship between a given couple of mRNA-miRNA).

We also computed an S score = − 2 (logratio_miRNA_ x logratio_mRNA_) to have a weigh within each couple of mRNA and miRNA, representing the level of alteration among Li responders and non-responders (the higher the score the higher the deregulation). We considered a positive S score (> 0), to take into account only mRNA-miRNA pairs within which mRNA and miRNA showed an opposite direction of expression, in terms of FC [49].

We extracted the most relevant mRNA-miRNA pairs by filtering for Pearson correlation coefficient ρ ≤ − 0.38, showing an anti-correlation trend, a nominal *p*-value ≤0.05 and a positive S score.

## Results

### Transcriptomic signatures associated with lithium response

Our first aim was to identify changes at the transcriptome level that could be associated with Li response. From the comparison between BD responder and non-responder patients, we identified 335 differentially modulated genes (217 were up-regulated and 118 down-regulated) (Supplementary Table 1 for the entire list of significant genes) in LCLs of Li responders.

To strengthen our analysis, we compared our results to already available transcriptomic studies by performing a bibliographic search in PubMed, using the following keywords: “*lithium; bipolar disorder; peripheral blood; gene expression*” OR “*lithium; bipolar disorder; lymphoblastoid; gene expression*”. We found 15 studies conducted in peripheral blood samples and 21 performed in LCLs obtained from patients diagnosed with BD, responders or non-responders to Li, but also from controls. When we looked for common genes within our list and already available studies, we found an overlap of 42 Li-responsive genes modulated in the same direction upon treatment with Li (Supplementary Table 2) [29, 30, 32, 33, 54–57].

### mRNAs pathway and network analyses

**Fig. 1:**
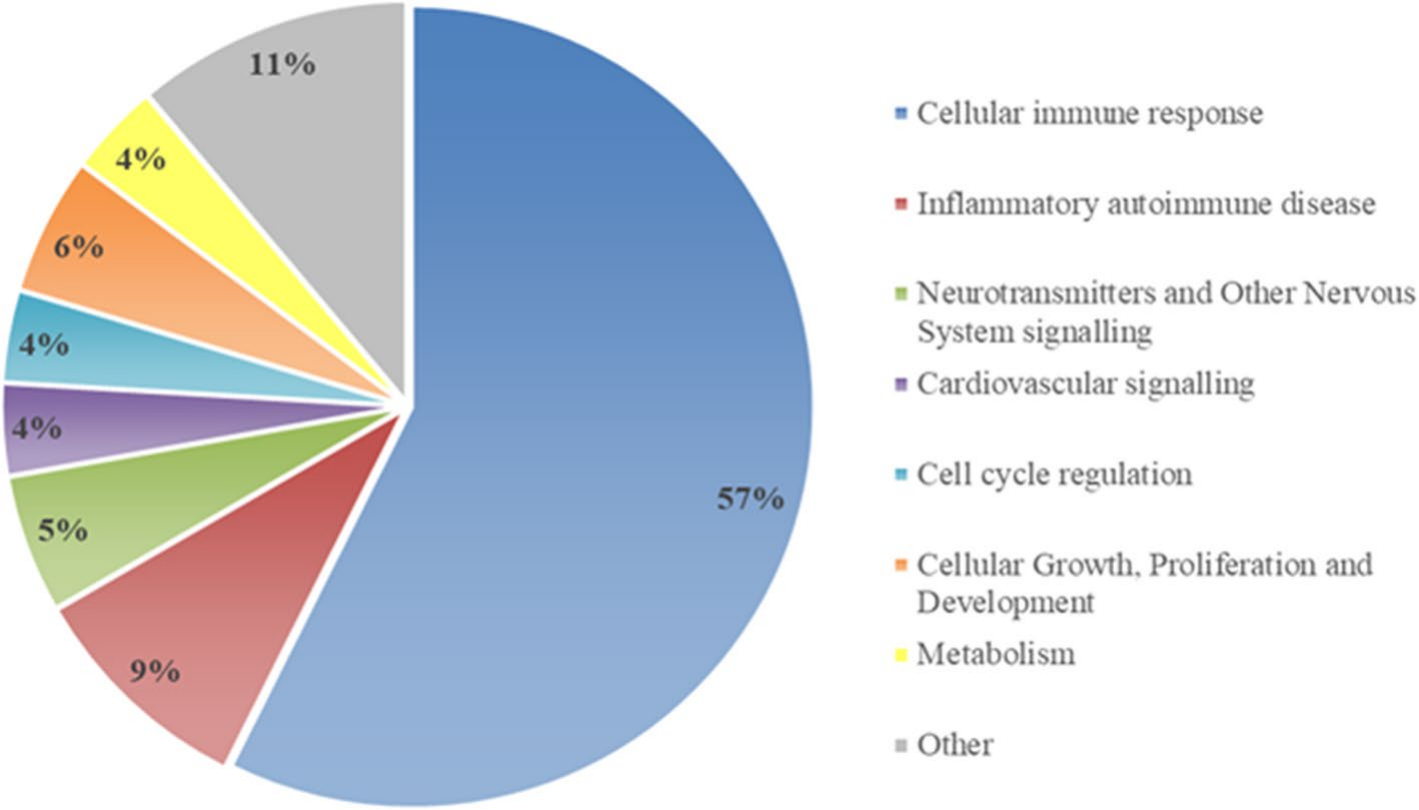
Pie chart gathering 54 significant pathways, identified in patients diagnosed with BD who respond to Li, according to their biological functions

To identify possible deregulated biological processes underlying Li treatment response, we performed a pathway and network analysis on the list of 335 differentially expressed genes. We found 54 significantly modulated pathways represented in the pie chart (Fig. 1) and in Supplementary Table 3. As shown in the pie chart, 57% of the pathways are involved in cellular immune response, including Th1 and Th2 pathways, OX40 signalling, B cell development, T helper cell differentiation, Antigen Presentation, Calcium-induced T Lymphocyte Apoptosis, Inflammasome pathway and Nur77 signalling in T Lymphocytes. Other pathways relevant to BD pathophysiology and Li treatment response are related to neurotransmitters and other nervous system signalling (5%) (i.e. ErbB signalling), cell cycle regulation (4%) (i.e. Cdc42 and 14–3-3 mediated signalling) and metabolism (4%) (i.e. Type I Diabetes Mellitus Signalling*).*

**Fig. 2:**
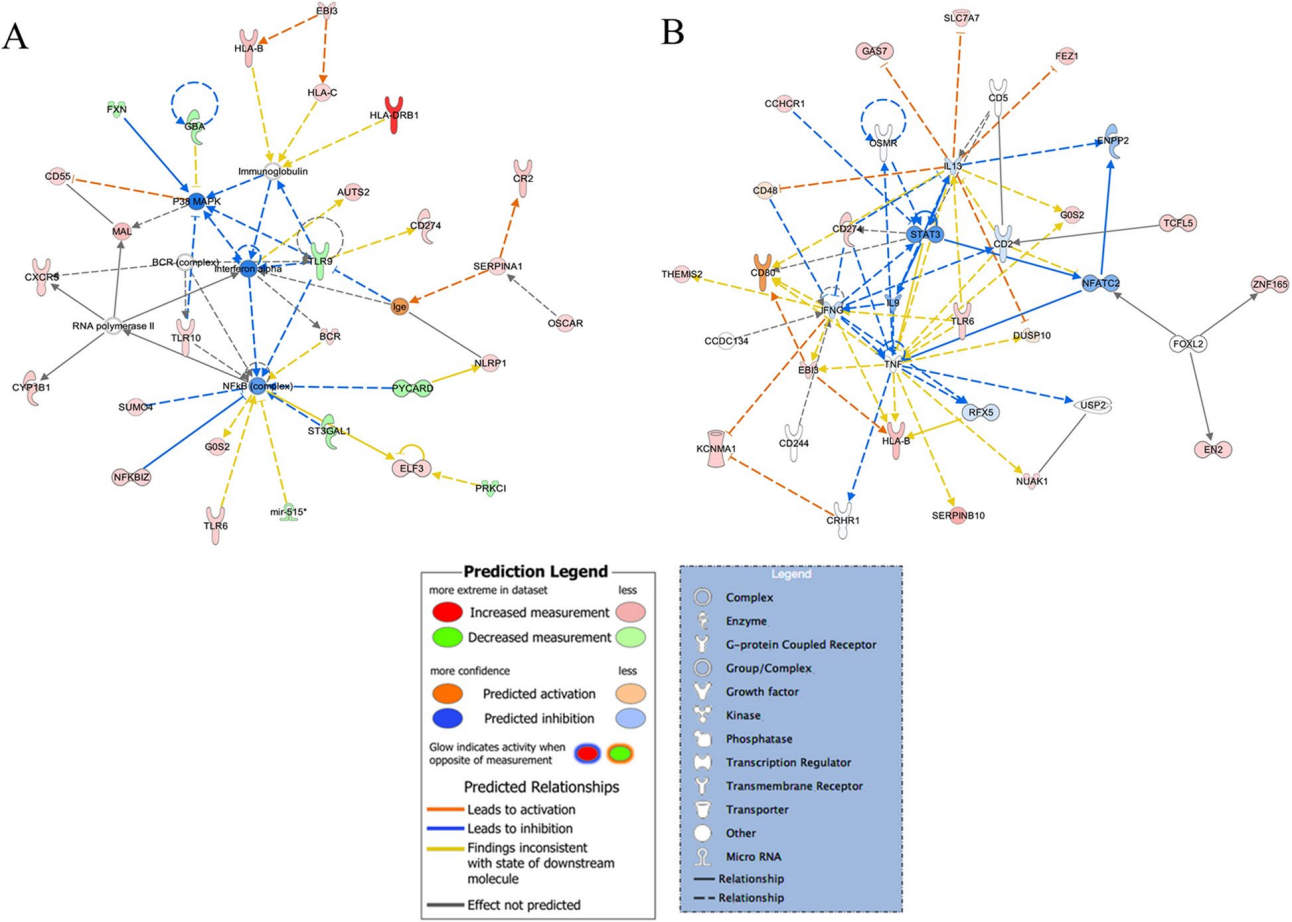
Networks implicated in response to Li treatment in BD responders. NF-kB, TNF and STAT3 are among the hub genes

In addition, to predict how genes in our data set could interact with each other, we performed a network analysis on the same list of 335 differentially expressed genes. Interestingly, IPA revealed 21 relevant functional networks (Supplementary Table 4). As shown in Fig. 2*,* Nuclear Factor kappa B (NF-κB), Tumor Necrosis Factor (TNF) and Signal Transducer and Activator of Transcription 3 (STAT3) might represent important functional nodes associated with Li treatment response.

Furthermore, to predict directional effects of the networks, we used Molecule Activity Predictor (MAP) and Path designer functionalities in IPA, which take into account the observed expression changes to compute functional effects on neighbouring molecules. Networks showing NF-κB, TNF and STAT3 among the hub genes resulted globally inhibited in patients diagnosed with BD who responded to Li treatment (Fig. 2).

### Results of eQTL-mapping studies from blood and/or brain tissues

To better investigate the effects of Li response on gene expression levels in BD responders and non-responders, we performed an *in-silico* analysis based on the matching between expression Quantitative Trait Loci (eQTL) and the genetic signals available from BD GWAS [58].

Our aim was to identify to which extent the genetic regulation of gene expression was associated with the genetic signal of the analyzed phenotype, represented by BD symptomatology, hypothesizing that Li treatment in BD responders could counterbalance alterations in gene expression levels relevant for BD. For our purpose, we retrieved the list of differentially regulated genes in blood and brain tissues (according to GTEx eQTL data) considering BD GWAS summary statistics from the Psychiatric Genomics Consortium [59]. We took into account only those genes that reached significance (nominally significant *p*-value < 0.05). We then compared this list with the whole data set of differentially expressed genes between BD responder and non-responder patients found in our transcriptome microarray analysis (Table 2). Specifically, we were interested in those genes with an opposite directionality between case/control and Li response. The only gene that survived this analysis was fasciculation and elongation protein zeta (FEZ1). A significant association by TWAS suggests that a down-regulation of FEZ1 expression levels increases the risk for BD (TWAS Z = − 2.15; *p*-value = 0.0318). Interestingly, in our analysis, we found a significant increase of FEZ1 mRNA levels in BD responders versus non-responders (FC = 1.51; p-value = 0.0314), suggesting FEZ1 as a potential target of Li therapeutic effects.

**Table 2:**
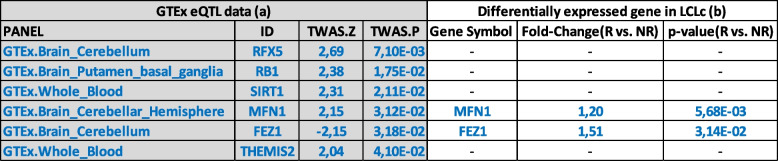
Common genes between GTEx data and differentially expressed genes in our analysis. TWAS: Transcriptome-Wide Association Studies; TWAS. Z is an estimation of the strength of association between the predicted expression of a gene and a complex trait. TWAS. P is the p-value associated with the TWAS test. On the left (**a**), genes that reached the significance in reference data set from GTEx; on the right (**b**), genes in common between GTEx eQTL data and our list of differentially expressed genes

### miRNomic signatures associated with Li response

In order to evaluate which miRNAs could be differentially modulated by Li, we also performed a miRNome microarray analysis in LCLs. We found a list of 77 mature miRNAs that were significantly modulated in BD responders versus non-responders. Out of these, 46 were up-regulated and 31 down-regulated (Supplementary Table 5). To verify whether these differentially expressed mature miRNAs have been already associated with Li response, we conducted a search in PubMed using the following keywords “*lithium; bipolar disorder; miRNAs*” and we found 17 articles. We identified only two common miRNAs modulated in the same direction (up-regulated in LCLs of patients diagnosed with BD following chronic Li treatment) between our list and previous studies: *hsa-miR-34a-5p* (FC = 1.26, *p*-value = 0.0378) and *hsa-miR-152-3p* (FC = 1.54, p-value = 0.007), which were consistent with the literature [60].

### MiRNAs pathway analysis

In order to investigate the biological systems regulated by differentially expressed miRNAs in BD responder patients, we conducted a pathway analysis by using Diana Tool software (miRPath v.3). Interestingly, we identified a list of 77 statistically significant pathways (Table 3 and Supplementary Table 6) mainly involved in: i) *neurodevelopment*, such as Hippo signalling pathway, Axon guidance, Glutamatergic synapse, Neurotrophin signalling pathway, FoxO signalling pathway, mTOR signalling pathway, GABAergic synapse, Cholinergic synapse, Wnt signalling pathway and Dopaminergic synapse; ii) *intracellular signal transduction* (i.e. ErbB signalling pathway, Ras signalling pathway, Rap1 signalling pathway, MAPK signalling pathway, cAMP signalling pathway, AMPK signalling pathway, PI3K-Akt signalling pathway and cGMP-PKG signalling pathway); iii) *inflammatory/immune system response*, including TGF-beta signalling pathway and T cell receptor signalling pathway.

**Table 3:** List of 77 statistically significant pathways targeted by differentially modulated miRNAs identified using Diana Tool software (miRPath v.3)

	KEGG pathway	***p***-value	genes	miRNAs
1	Mucin type O-Glycan biosynthesis	5.16E-15	27	41
2	Proteoglycans in cancer	1.17E-11	165	63
3	Pathways in cancer	1.43E-07	312	67
4	Arrhythmogenic right ventricular cardiomyopathy (ARVC)	5.37–07	62	56
5	Hippo signalling pathway	5.37E-07	124	64
6	Renal cell carcinoma	8.87E-07	60	56
7	ECM-receptor interaction	6.54E-06	66	55
8	Axon guidance	6.54E-06	104	60
9	ErbB signalling pathway	1.03E-05	75	55
10	TGF-beta signalling pathway	1.67E-05	68	55
11	Thyroid hormone signalling pathway	1.67E-05	98	61
12	Signalling pathways regulating pluripotency of stem cells	3.42E-05	114	61
13	Adherens junction	5.07E-05	65	60
14	Focal adhesion	5.07E-05	165	64
15	Ras signalling pathway	5.07E-05	177	68
16	Rap1 signalling pathway	5.27E-05	167	62
17	Lysine degradation	1.13E-04	39	57
18	Bacterial invasion of epithelial cells	3.08E-04	65	60
19	Glutamatergic synapse	3.79E-04	90	60
20	Endocytosis	3.79E-04	163	62
21	Glioma	4.34E-04	53	53
22	Adrenergic signalling in cardiomyocytes	6.85E-04	113	61
23	Pancreatic cancer	7.03E-04	56	54
24	Morphine addiction	7.88E-04	74	59
25	Phosphatidylinositol signalling system	8.54E-04	63	55
26	Adipocytokine signalling pathway	1.07E-03	58	57
27	Gap junction	1.78E-03	70	59
28	Oxytocin signalling pathway	2.12E-03	122	59
29	MAPK signalling pathway	2.13E-03	192	66
30	cAMP signalling pathway	2.20E-03	153	65
31	Platelet activation	2.36E-03	103	62
32	Circadian rhythm	3.00E-03	29	42
33	AMPK signalling pathway	3.00E-03	96	62
34	Neurotrophin signalling pathway	3.00E-03	96	65
35	FoxO signalling pathway	3.16E-03	105	59
36	Small cell lung cancer	3.21E-03	70	52
37	Regulation of actin cytoskeleton	4.44E-03	162	61
38	PI3K-Akt signalling pathway	4.69E-03	252	65
39	mTOR signalling pathway	4.73E-03	50	51
40	Estrogen signalling pathway	4.73E-03	76	58
41	Chronic myeloid leukemia	4.83E-03	61	54
42	Prostate cancer	4.89E-03	71	57
43	Ubiquitin mediated proteolysis	5.43E-03	105	60
44	Dorso-ventral axis formation	5.73E-03	25	37
45	Long-term depression	6.81E-03	46	51
46	Prolactin signalling pathway	6.81E-03	56	55
47	Protein processing in endoplasmic reticulum	8.06E-03	122	67
48	Acute myeloid leukemia	8.36E-03	47	52
49	Prion diseases	1.05E-02	22	38
50	Retrograde endocannabinoid signalling	1.05E-02	77	56
51	Colorectal cancer	1.05E-02	50	56
52	Choline metabolism in cancer	1.05E-02	79	60
53	Dilated cardiomyopathy	1.21E-02	70	52
54	GABAergic synapse	1.25E-02	64	54
55	Other types of O-glycan biosynthesis	1.43E-02	24	36
56	T cell receptor signalling pathway	1.43E-02	81	53
57	Gastric acid secretion	1.58E-02	60	54
58	Endometrial cancer	1.58E-02	43	56
59	Circadian entrainment	1.63E-02	77	62
60	Amoebiasis	1.64E-02	78	55
61	p53 signalling pathway	1.64E-02	55	51
62	Chagas disease (American trypanosomiasis)	1.67E-02	77	52
63	Non-small cell lung cancer	1.73E-02	44	51
64	Cholinergic synapse	1.92E-02	85	57
65	Glycosaminoglycan biosynthesis - heparan sulfate / heparin	2.30E-02	19	40
66	Hepatitis B	3.16E-02	102	61
67	cGMP-PKG signalling pathway	3.16E-02	122	63
68	Calcium signalling pathway	3.31E-02	131	61
69	Melanoma	3.46E-02	56	52
70	HIF-1 signalling pathway	3.56E-02	79	55
71	Hypertrophic cardiomyopathy (HCM)	3.63E-02	63	51
72	Shigellosis	3.68E-02	50	52
73	Cocaine addiction	3.82E-02	37	46
74	Wnt signalling pathway	4.33E-02	105	62
75	Caffeine metabolism	4.71E-02	5	10
76	Dopaminergic synapse	4.71E-02	97	65
77	Thyroid hormone synthesis	4.86E-02	52	50

### Predicted mRNA-miRNA interactions

The innovative approach of our study mainly consists in the integration analysis between transcriptomic and miRNomic data carried out on the same patients diagnosed with BD. Briefly, by using miRComb R package [49], we combined transcriptomic and miRNomic expression data with microarray hybridization information to compute all possible correlations between deregulated and differentially modulated mRNAs and miRNAs. Our goal was to highlight those mRNA-miRNA interactions that may play an important role in the outcome of Li treatment response.

**Fig. 3:**
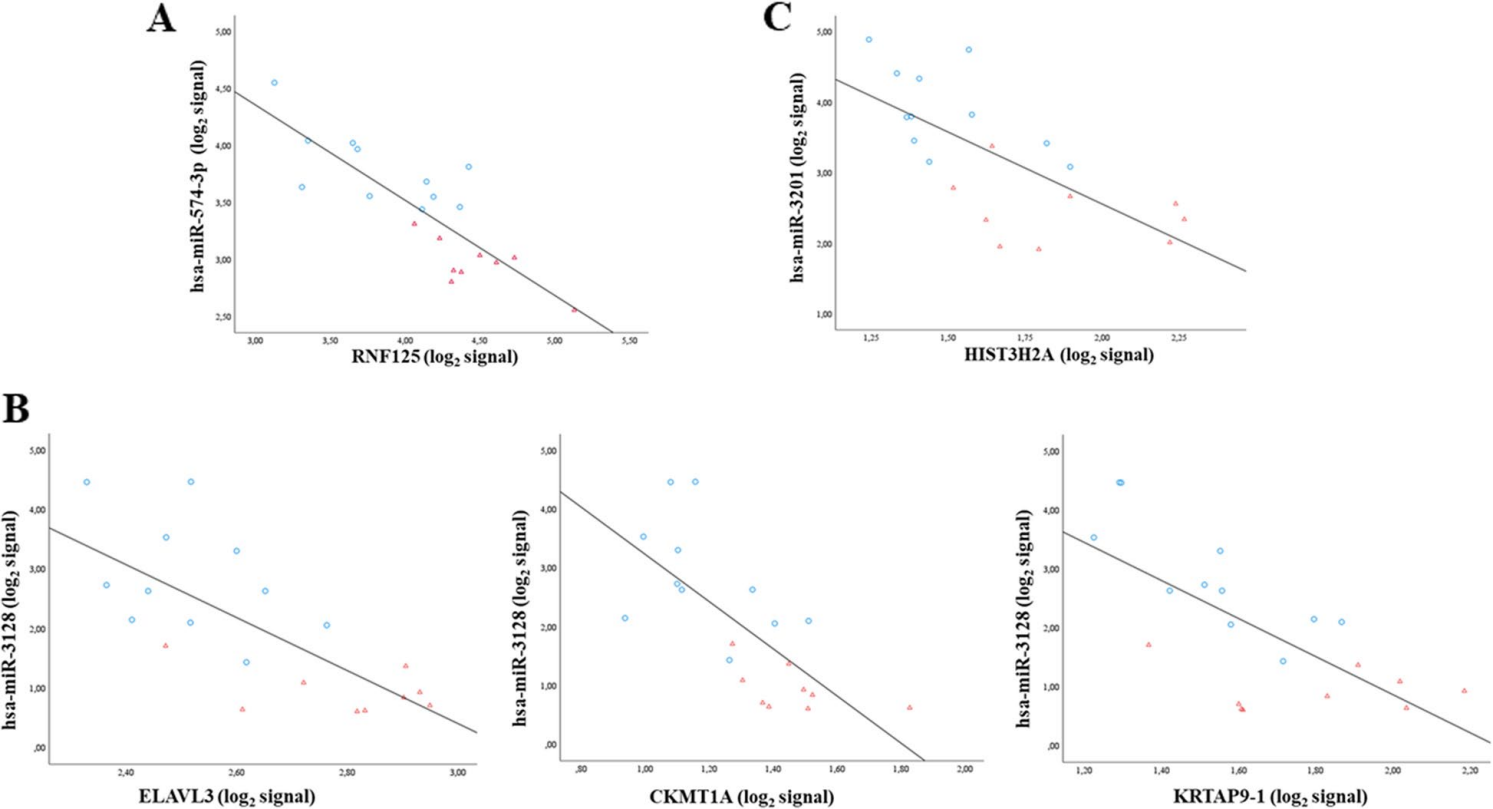
Top 5 mRNA-miRNA miRComb interactions. Panel **A** shows the interaction between *hsa-miR-574-3p* and RNF125. Panel **B** shows interactions between *hsa-miR-3128*, ELAVL3, CKMT1A and KRTAP9–1, respectively. Panel **C** shows the correlation between *hsa-miR-3201* and HIST3H2A. Li responders are depicted in blue circles, whereas non-responders are shown in red triangles. The graphs show the correlation between normalized values of log2 intensity signal. Regression line shows the negative correlation

We thus selected the previously mentioned 335 and 77 significantly deregulated mRNAs and miRNAs, respectively, and we computed all possible correlations. Among all mRNA-miRNA possible interactions, 7627 had a *p*-value ≤0.05. We considered only mature mRNA-miRNA pairs and we eliminated the isoforms of the same gene, obtaining 5450 significant mature mRNA-miRNA interactions with a Pearson correlation index ρ ≤ − 0.38 (Supplementary Table *7*). In order to further reduce the number of interactions, we used a cut-off of p-value ≤0.05 and filtered mRNA-miRNA pairs by Pearson correlation index ρ ≤ − 0.5. We identified a list of 2027 significant mature mRNA-miRNA interactions (Supplementary Table 8). Moreover, as miRComb does not take into account competitivity among different miRNAs targeting the same gene, we also used the S score (> 0), an index representing the level of alteration among mRNAs and miRNAs, to further prioritize the mRNA-miRNA pairs in those cases where: i) mRNA and miRNA within the pair showed an opposite direction of expression and ii) different miRNAs shared the same mRNA target: higher score means that both mRNA and miRNA are highly deregulated in Li responders. We obtained 97 mRNA-miRNA interactions representing those that are more likely to occur in the context of good response to Li treatment (Table 4). As an example, we show some plots of the most significant pairs in that table, namely *hsa-miR-574-3p* and Ring Finger protein 125 (RNF125) (ρ = − 0.83), *hsa-miR-3128* and ELAV Like RNA Binding Protein 3 (ELAVL3) (ρ = − 0.72), Creatine Kinase, Mitochondrial 1A (CKMT1A) (ρ = − 0.71), Keratin Associated Protein 9–1 (KRTAP9–1) (ρ = − 0.70), *hsa-miR-3201* and Histone Cluster 3, H2a (HIST3H2A) (ρ = − 0.69) (Fig. 3).

**Table 4:** Top 97 mRNA-miRNA pairs with the most significant negative correlations predicted by miRComb, ranked by the Pearson correlation index and the S score. All interactions show a *p*-value ≤ 0.05, a Pearson correlation index *ρ* ≤ -0.5 and an S score > 0

	Mature miRNA	mRNA	cor	***p***-value	logratio.miRNA	meanExp.miRNA	logratio.mRNA	meanExp.mRNA	adj.***p***-value	S score	FC.miRNA (R vs NR)	FC.mRNA (R vs NR)
1	hsa-miR-574-3p	RNF125	-0.83	2.53E-06	0.83	3.42	-0.65	4.12	0.04	1.07	1.78	-1.56
2	hsa-miR-3128	ELAVL3	-0.72	1.62E-04	1.92	2.00	-0.28	2.64	0.09	1.06	3.78	-1.21
3	hsa-miR-3128	CKMT1A	-0.71	2.26E-04	1.92	2.00	-0.28	1.31	0.10	1.06	3.78	-1.21
4	hsa-miR-3128	KRTAP9-1	-0.70	3.12E-04	1.92	2.00	-0.27	1.65	0.10	1.03	3.78	-1.20
5	hsa-miR-3201	HIST3H2A	-0.69	3.99E-04	1.46	3.24	-0.38	1.66	0.11	1.11	2.75	-1.30
6	hsa-miR-3128	RASGEF1A	-0.68	5.44E-04	1.92	2.00	-0.42	3.71	0.12	1.62	3.78	-1.34
7	hsa-miR-3128	AQP1	-0.66	7.06E-04	1.92	2.00	-0.28	2.74	0.12	1.07	3.78	-1.21
8	hsa-miR-3201	MIR4461	-0.66	7.40E-04	1.46	3.24	-0.39	5.53	0.12	1.13	2.75	-1.31
9	hsa-miR-3128	STK39	-0.66	7.76E-04	1.92	2.00	-0.26	2.14	0.12	1.02	3.78	-1.20
10	hsa-miR-3128	MIR516B2	-0.65	8.80E-04	1.92	2.00	-0.26	1.71	0.13	1.01	3.78	-1.20
11	hsa-miR-3128	LOC100132314	-0.65	9.39E-04	1.92	2.00	-0.31	1.30	0.13	1.20	3.78	-1.24
12	hsa-miR-3128	ZC2HC1B	-0.65	1.03E-03	1.92	2.00	-0.49	3.33	0.13	1.88	3.78	-1.41
13	hsa-miR-3128	ZNF487P	-0.63	1.42E-03	1.92	2.00	-0.29	1.76	0.15	1.12	3.78	-1.22
14	hsa-miR-3128	SPC25	-0.63	1.44E-03	1.92	2.00	-0.33	4.59	0.15	1.26	3.78	-1.26
15	hsa-miR-3128	SULT1A1	-0.63	1.45E-03	1.92	2.00	-0.38	2.72	0.15	1.47	3.78	-1.30
16	hsa-miR-3128	CERS6	-0.63	1.46E-03	1.92	2.00	-0.38	7.55	0.15	1.45	3.78	-1.30
17	hsa-miR-3128	FXN	-0.62	1.62E-03	1.92	2.00	-0.28	5.44	0.15	1.07	3.78	-1.21
18	hsa-miR-574-3p	SAMD12	-0.62	1.76E-03	0.83	3.42	-0.79	4.27	0.15	1.30	1.78	-1.72
19	hsa-miR-3128	LINC01011	-0.62	1.81E-03	1.92	2.00	-0.30	2.10	0.15	1.16	3.78	-1.23
20	hsa-miR-3613-3p	RAB42	-0.62	1.90E-03	1.20	7.84	-0.47	3.23	0.15	1.14	2.30	-1.39
21	hsa-miR-8084	ZC2HC1B	-0.62	1.90E-03	1.03	3.55	-0.49	3.33	0.15	1.01	2.04	-1.41
22	hsa-miR-3128	CHAC2	-0.62	1.93E-03	1.92	2.00	-0.30	4.21	0.15	1.15	3.78	-1.23
23	hsa-miR-3613-3p	RASGEF1A	-0.61	1.96E-03	1.20	7.84	-0.42	3.71	0.15	1.02	2.30	-1.34
24	hsa-miR-574-3p	GAPT	-0.61	1.98E-03	0.83	3.42	-0.62	3.48	0.15	1.03	1.78	-1.54
25	hsa-miR-3128	ZNF416	-0.61	2.17E-03	1.92	2.00	-0.29	3.21	0.16	1.10	3.78	-1.22
26	hsa-miR-3128	MIR4461	-0.61	2.21E-03	1.92	2.00	-0.39	5.53	0.16	1.48	3.78	-1.31
27	hsa-miR-204-3p	LOC389906	-0.61	2.33E-03	0.55	2.00	-0.95	3.70	0.16	1.04	1.46	-1.93
28	hsa-miR-3128	ESYT1	-0.61	2.35E-03	1.92	2.00	-0.27	5.90	0.16	1.05	3.78	-1.21
29	hsa-miR-3128	MNX1	-0.60	2.53E-03	1.92	2.00	-0.26	3.73	0.16	1.01	3.78	-1.20
30	hsa-miR-3201	PRAMEF22	-0.60	2.56E-03	1.46	3.24	-0.36	4.14	0.16	1.04	2.75	-1.28
31	hsa-miR-3201	IMPA2	-0.60	2.56E-03	1.46	3.24	-0.46	4.75	0.16	1.35	2.75	-1.38
32	hsa-miR-3128	LOC100132781	-0.59	2.87E-03	1.92	2.00	-0.31	2.09	0.16	1.19	3.78	-1.24
33	hsa-miR-3128	KRT16P3	-0.59	2.98E-03	1.92	2.00	-0.27	2.20	0.16	1.03	3.78	-1.21
34	hsa-miR-3201	PIK3CG	-0.59	3.00E-03	1.46	3.24	-0.35	6.20	0.16	1.03	2.75	-1.28
35	hsa-miR-3201	RASGEF1A	-0.59	3.07E-03	1.46	3.24	-0.42	3.71	0.16	1.24	2.75	-1.34
36	hsa-miR-3128	PRR5L	-0.59	3.09E-03	1.92	2.00	-0.29	2.78	0.16	1.12	3.78	-1.23
37	hsa-miR-3605-3p	HLA-DRB1	-0.58	3.60E-03	-0.28	0.57	2.72	4.04	0.17	1.50	-1.21	6.58
38	hsa-miR-27a-3p	IGKV1-5	-0.58	3.95E-03	0.30	5.94	-2.37	6.00	0.17	1.41	1.23	-5.17
39	hsa-miR-3128	RN5S358	-0.57	4.08E-03	1.92	2.00	-0.33	1.82	0.18	1.25	3.78	-1.25
40	hsa-miR-6893-5p	TRAJ52	-0.57	4.12E-03	-0.71	3.12	0.92	2.95	0.18	1.31	-1.63	1.89
41	hsa-miR-3201	ZC2HC1B	-0.57	4.16E-03	1.46	3.24	-0.49	3.33	0.18	1.43	2.75	-1.41
42	hsa-miR-629-3p	NAALADL2-AS2	-0.57	4.19E-03	-0.49	2.74	1.19	6.73	0.18	1.16	-1.40	2.28
43	hsa-miR-3128	HIST3H2A	-0.57	4.22E-03	1.92	2.00	-0.38	1.66	0.18	1.46	3.78	-1.30
44	hsa-miR-4423-3p	IGLV4-60	-0.57	4.46E-03	0.98	2.02	-0.59	4.19	0.18	1.16	1.98	-1.50
45	hsa-miR-3128	MIR4299	-0.56	4.77E-03	1.92	2.00	-0.28	2.85	0.18	1.07	3.78	-1.21
46	hsa-miR-3128	PMCHL1	-0.56	5.22E-03	1.92	2.00	-0.33	1.72	0.18	1.25	3.78	-1.25
47	hsa-miR-3613-3p	SAMD12	-0.56	5.30E-03	1.20	7.84	-0.79	4.27	0.19	1.89	2.30	-1.72
48	hsa-miR-3201	GBP3	-0.56	5.31E-03	1.46	3.24	-0.34	4.94	0.19	1.00	2.75	-1.27
49	hsa-miR-3128	CTF1	-0.56	5.41E-03	1.92	2.00	-0.29	4.12	0.19	1.11	3.78	-1.22
50	hsa-miR-342-5p	IGLV1-44	-0.56	5.44E-03	0.46	3.63	-2.32	2.92	0.19	2.12	1.37	-5.00
51	hsa-miR-3128	PIK3CG	-0.55	5.75E-03	1.92	2.00	-0.35	6.20	0.19	1.35	3.78	-1.28
52	hsa-miR-16-2-3p	HLA-DRB5	-0.55	5.76E-03	-0.46	2.88	2.56	3.12	0.19	2.37	-1.38	5.88
53	hsa-miR-3605-3p	HLA-DRB5	-0.55	5.79E-03	-0.28	0.57	2.56	3.12	0.19	1.41	-1.21	5.88
54	hsa-miR-6893-5p	LOC643401	-0.55	5.84E-03	-0.71	3.12	0.76	4.58	0.19	1.07	-1.63	1.69
55	hsa-miR-6774-5p	NAALADL2-AS2	-0.55	5.94E-03	-0.57	2.02	1.19	6.73	0.19	1.36	-1.49	2.28
56	hsa-miR-3613-3p	TNFRSF21	-0.55	6.03E-03	1.20	7.84	-0.62	4.75	0.19	1.49	2.30	-1.54
57	hsa-miR-3201	CERS6	-0.55	6.04E-03	1.46	3.24	-0.38	7.55	0.19	1.10	2.75	-1.30
58	hsa-miR-4668-5p	CLEC6A	-0.55	6.05E-03	0.78	7.49	-0.84	3.14	0.19	1.32	1.72	-1.79
59	hsa-miR-3128	LOC253039	-0.55	6.26E-03	1.92	2.00	-0.47	4.91	0.19	1.81	3.78	-1.39
60	hsa-miR-6893-5p	NAALADL2-AS2	-0.55	6.38E-03	-0.71	3.12	1.19	6.73	0.19	1.68	-1.63	2.28
61	hsa-miR-6893-5p	CR2	-0.55	6.43E-03	-0.71	3.12	0.76	6.15	0.19	1.08	-1.63	1.70
62	hsa-miR-6746-5p	HLA-DRB1	-0.54	6.51E-03	-0.36	3.38	2.72	4.04	0.19	1.94	-1.28	6.58
63	hsa-miR-635	IGLV1-44	-0.54	6.56E-03	0.30	0.56	-2.32	2.92	0.20	1.40	1.23	-5.00
64	hsa-miR-574-3p	LOC642838	-0.54	6.62E-03	0.83	3.42	-0.73	3.65	0.20	1.21	1.78	-1.66
65	hsa-miR-4668-5p	SAMD12	-0.53	7.62E-03	0.78	7.49	-0.79	4.27	0.20	1.23	1.72	-1.72
66	hsa-miR-3128	SLC22A5	-0.53	8.05E-03	1.92	2.00	-0.32	3.43	0.20	1.22	3.78	-1.25
67	hsa-miR-8063	TRAJ52	-0.53	8.11E-03	-0.64	3.32	0.92	2.95	0.20	1.18	-1.56	1.89
68	hsa-miR-659-3p	IGLV1-44	-0.53	8.49E-03	0.28	1.30	-2.32	2.92	0.21	1.32	1.22	-5.00
69	hsa-miR-3201	SULT1A1	-0.53	8.49E-03	1.46	3.24	-0.38	2.72	0.21	1.12	2.75	-1.30
70	hsa-miR-4423-3p	CLEC6A	-0.52	8.74E-03	0.98	2.02	-0.84	3.14	0.21	1.65	1.98	-1.79
71	hsa-miR-181a-3p	HECW2	-0.52	8.77E-03	-0.50	1.56	1.02	4.89	0.21	1.01	-1.41	2.03
72	hsa-miR-4423-3p	FOXP1	-0.52	9.02E-03	0.98	2.02	-0.58	4.99	0.21	1.13	1.98	-1.49
73	hsa-miR-6785-5p	IGLV1-44	-0.52	9.16E-03	0.39	3.10	-2.32	2.92	0.21	1.79	1.31	-5.00
74	hsa-miR-331-3p	IGKV2D-29	-0.52	9.20E-03	0.56	2.87	-1.70	2.36	0.21	1.90	1.47	-3.25
75	hsa-miR-5088-5p	HLA-DRB1	-0.52	9.33E-03	-0.41	1.24	2.72	4.04	0.21	2.23	-1.33	6.58
76	hsa-miR-34a-5p	IGKV2-29	-0.52	9.68E-03	0.33	5.61	-1.55	3.92	0.21	1.04	1.26	-2.94
77	hsa-miR-4462	SERPINB10	-0.52	9.71E-03	-0.51	2.40	1.21	6.50	0.21	1.22	-1.42	2.31
78	hsa-miR-16-2-3p	HLA-DRB1	-0.52	9.79E-03	-0.46	2.88	2.72	4.04	0.21	2.52	-1.38	6.58
79	hsa-miR-3128	IMPA2	-0.52	9.83E-03	1.92	2.00	-0.46	4.75	0.21	1.78	3.78	-1.38
80	hsa-miR-195-3p	SERPINB10	-0.52	9.89E-03	-0.46	1.41	1.21	6.50	0.21	1.11	-1.38	2.31
81	hsa-miR-3128	RN5S431	-0.52	9.91E-03	1.92	2.00	-0.27	1.30	0.21	1.04	3.78	-1.21
82	hsa-miR-3128	CCR6	-0.52	9.94E-03	1.92	2.00	-0.33	5.36	0.21	1.26	3.78	-1.25
83	hsa-miR-3201	CHL1-AS1	-0.52	1.00E-02	1.46	3.24	-0.35	2.12	0.21	1.02	2.75	-1.27
84	hsa-miR-3201	LAMB1	-0.51	1.03E-02	1.46	3.24	-0.39	2.44	0.21	1.14	2.75	-1.31
85	hsa-miR-3128	LOC100506571	-0.51	1.05E-02	1.92	2.00	-0.27	3.73	0.21	1.04	3.78	-1.21
86	hsa-miR-3128	RN5S128	-0.51	1.06E-02	1.92	2.00	-0.50	2.05	0.21	1.90	3.78	-1.41
87	hsa-miR-6774-5p	NRN1	-0.51	1.06E-02	-0.57	2.02	0.93	4.30	0.21	1.07	-1.49	1.91
88	hsa-miR-421	IGKV1-5	-0.51	1.12E-02	0.49	1.63	-2.37	6.00	0.22	2.32	1.40	-5.17
89	hsa-miR-3201	CLEC6A	-0.51	1.13E-02	1.46	3.24	-0.84	3.14	0.22	2.46	2.75	-1.79
90	hsa-miR-6893-5p	MAL	-0.51	1.14E-02	-0.71	3.12	0.92	5.08	0.22	1.30	-1.63	1.89
91	hsa-miR-550a-3-5p	IGLV1-44	-0.51	1.15E-02	0.28	0.98	-2.32	2.92	0.22	1.29	1.21	-5.00
92	hsa-miR-4785	IGLV1-44	-0.50	1.16E-02	0.51	2.09	-2.32	2.92	0.22	2.35	1.42	-5.00
93	hsa-miR-3128	ANK3	-0.50	1.18E-02	1.92	2.00	-0.27	2.14	0.22	1.02	3.78	-1.20
94	hsa-miR-3128	HIST1H3G	-0.50	1.18E-02	1.92	2.00	-0.30	7.85	0.22	1.16	3.78	-1.23
95	hsa-miR-3201	RN5S128	-0.50	1.20E-02	1.46	3.24	-0.50	2.05	0.22	1.45	2.75	-1.41
96	hsa-miR-3201	SAMD12-AS1	-0.50	1.21E-02	1.46	3.24	-0.58	1.78	0.22	1.68	2.75	-1.49
97	hsa-miR-3128	LINC00891	-0.50	1.22E-02	1.92	2.00	-0.27	3.96	0.22	1.03	3.78	-1.21

## Discussion

In this exploratory study, we investigated the effects of Li response in LCLs derived from patients diagnosed with BD, responders or non-responders to therapeutic treatment, to further elucidate the biological and molecular processes involved in Li therapeutic action. We used an integrative approach on both mRNAs and miRNAs microarray data by combining differential expression analyses with correlation of expression levels. The premise is that the expression changes of specific mRNAs and miRNAs will reflect alterations in specific target genes and biological pathways that underlie therapeutic action, thus, we sought to identify differentially regulated mRNAs and miRNAs in LCLs from BD responder and non-responder patients. This molecular signature may allow the identification of biomarkers able to shed light on both Li therapeutic effects and BD pathophysiology.

Through an exploratory transcriptome microarray analysis, we identified 335 differentially expressed genes between BD responder and non-responder patients and 42 of them were in common with previously published transcriptomic studies performed in LCLs or in peripheral blood samples of patients diagnosed with BD [29, 30, 32, 33, 54–57], supporting the reliability and reproducibility of our analysis. Among all common genes, the most studied and recurring ones were represented by Branched Chain Amino Acid Transaminase 1 (BCAT1), which was up-regulated in our data set and in three studies, all performed in LCLs [33, 54, 55] and by RAB11 Family Interacting Protein 1 (RAB11FIP1) whose up-regulation was reported by our study and by three different works, all performed in LCLs as well [29, 33, 55].

The pathway and network analyses performed on the list of 335 differentially expressed genes revealed that they are mainly involved in cellular immune response and, to a lesser extent, in neurotransmitters and other nervous system signalling, cell cycle regulation and metabolism. In support of these findings, our data suggest that the therapeutic effects of Li mainly affect the immune system and the inflammatory response: indeed, functional molecular nodes with a biological role in the treatment response to Li are represented by NF-κB, TNF and STAT3, known for their pro-inflammatory role in mental disorders [61, 62]. Interestingly, networks related to these molecules resulted globally inhibited in patients diagnosed with BD who responded to Li treatment, confirming the anti-inflammatory properties of Li [62].

Worth of note is that top canonical pathways identified in our study are also represented among the most significantly ones obtained from the cross-trait meta-GWAS and pathway analysis based on GWAS summary statistics and treatment response to Li coming from GWAS of schizophrenia and from ConLiGen, respectively [63]. Briefly, in this study, the authors tested whether a polygenic score for schizophrenia was associated with Li treatment response in patients affected by BD and explored the potential molecular underpinnings of this association [63]. According to their results, patients diagnosed with BD, who had a low polygenic load for schizophrenia, responded better to Li treatment. Moreover, the authors found that genetic variants in the Human Leukocyte Antigens (HLA) genes, the antigen presentation pathway, and inflammatory cytokines such as TNF, IL-4 and IFN-γ could represent central and functional hub genes, suggesting that pathways associated with immunity and inflammation could play a key role in Li treatment response [63].

In this regard, previous studies have reported modulatory anti-inflammatory effects of Li and highlighted the possibility that mechanisms involving pro-inflammatory cytokines might play a role in mediating the responsiveness of Li in patients suffering from BD (for a review see [62]). These mechanisms are not clearly understood, but it is commonly thought to be dependent upon the inhibition of Glycogen synthase kinase-3β (GSK-3β), the well-established pharmacological target of Li. GSK-3β, a serine/threonine kinase ubiquitously distributed in mammalian tissues, is able to enhance inflammation and immune responses [64] through the activation of NF-κB (for a review see [62, 65]). In a simplified model, Li might reduce the production of pro-inflammatory mediators by inhibiting GSK-3β, thus resulting in a decreased activity of NF-κB, which leads to an attenuated expression of inflammatory-associated molecules and enzymes [62].

According to this hypothesis, Guloksuz and colleagues found higher plasma levels of TNF-α in patients diagnosed with BD characterized for a poor response to Li compared to those showing a good response, suggesting that increased TNF-α levels may affect the clinical response to Li [66].

Furthermore, from the analysis of tissue specific eQTL (GTEx) and GWAS association data [59], we found that decreased expression levels of FEZ1, a well-recognized risk factor for schizophrenia [67, 68] also involved in neuronal development, neuropathology, and viral infections [69], increases the risk for BD. Interestingly, in our transcriptome analysis we observed a significant increase in the expression levels of FEZ1 in BD responders to Li treatment. Although no eQTL and GWAS data are already available for FEZ1 and Li treatment response, we can hypothesize that, if validated and replicated in larger cohorts of BD responder patients, this gene might be involved in Li therapeutic effects.

Using a similar approach, we also identified 77 miRNAs whose expression differentially changed between BD Li responders and non-responders. These findings indicate that epigenetic modifications may play a key role in modulating the effect of mood stabilizers, shed light on their molecular targets and possibly explain the high heterogeneity in the treatment response to Li. Among the most significant biological processes modulated by these miRNAs in BD responders, we identified several pathways involved in neurodevelopment, as the WNT signalling, highlighting the key role of this pathway in Li response, and in inflammatory/immune system response, corroborating transcriptomic findings.

Following the same study design previously used for the transcriptome analysis, we found that, in line with previous studies [60, 70, 71], hsa-miR-34a-5p and hsa-miR-152-3p were up-regulated in BD responders compared to non-responders. Interestingly, high levels of miR-34a have been found in postmortem cerebellar tissue from patients diagnosed with BD, as well as in BD patient-derived neuronal cultures generated by reprogramming human fibroblasts into induced pluripotent stem cells (iPSCs) subsequently differentiated into neurons [72]. Although contrasting results exist, several studies have suggested that Li has a modulatory effect on *miR-34a* expression levels [73, 74], which have been found consistently up-regulated in LCLs following Li treatment [60]. In non-neuronal cells, *miR-34a* has been shown to be a strong inhibitor of the WNT signalling and β-catenin-mediated transcription in response to p53 activation [75, 76]. Given the well-known effects of Li on the WNT signalling, through the inhibition of GSK-3β, a key molecule within this pathway [77, 78], we can speculate that *miR-34a* might contribute to the therapeutic effects of Li [60, 72–74, 79, 80].

In addition to *miR-34a*, we found that *miR-152-3p* expression levels were consistently up-regulated in LCLs from Li responders. To our knowledge, no study has investigated the effect of Li on the modulation of this miRNA. However, *miR-152-3p* targets a DNA methyltransferase (DNMT1) and could interfere with gene expression levels and with Li treatment response by acting through DNA methylation. Indeed, it is well established that Li monotherapy induces a global DNA hypomethylation in leukocytes and converging evidence from clinical and preclinical studies has suggested that different mood stabilizers may exert specific effects on the epigenome [81, 82]. According to all these findings, our hypothesis, which, however, needs to be further examined, is that Li response could be, at least in part, mediated by epigenetic mechanisms, which may interact each other in a miRNA-epigenetics regulatory circuit, influencing the expression of relevant genes possibly involved in Li response.

However, the core analysis of the present work is based on an innovative approach that enables correlation analysis between transcriptome and miRNome data, simultaneously carried out on the same patients, in order to highlight those mRNA-miRNA interactions that may play an important role in the outcome of Li treatment response. To date, only two studies have already integrated miRNAs and mRNAs expression profiling in LCLs from BD responder or non responder patients [30, 83]. However, their main findings have not been replicated by our study. Although the rationale for the experimental design is based on a similar hypothesis, these studies show differences with our study. Indeed, Hunsberger and collaborators applied GRANITE, an integrative genomic systems biology-based tool, to genome-wide mRNA and miRNA expression data obtained from LCLs of BD excellent responders or non-responders to Li treatment. They found that the Let-7 miRNA family was consistently downregulated by Li in the BD responder group. Conversely to our study, LCLs were cultured for 7 days with either a therapeutic dose of Li or vehicle in order to highlight genetic networks differentially influenced by the treatment in the responder and non-responder patients [30]. In the other study, Pisanu and colleagues sequenced small non-coding RNAs with next generation sequencing (NGS) in LCLs from patients diagnosed with BD and characterized for Li response. Then, they integrated these data with differentially expressed mRNAs identified by microarray-based techniques in the same subject. The results suggest that miR-320a, miR-155-3p and their target genes might represent relevant players in modulating clinical response to Li [83]. In our study, we used a microarray-based approach for both transcriptomic and miRNomic analyses, taking advantage of the same microarray platform (GeneAtlas platform, Thermo Fisher Scientific, USA). Therefore, the use of the same platform for both analyses (mRNAs and miRNAs) has allowed us to follow simpler and leaner bioinformatics and data integration analyses. In addition, while Pisanu and collaborators focused their attention only on the most promising genes, we decided to perform a more complex approach by investigating biological pathways modulated by differentially expressed mRNAs and miRNAs, for a better understanding of the biological systems involved in Li treatment response.

MiRNAs that most likely occur in our data set of mRNA-miRNA interactions are *hsa-miR-574-3p, hsa-miR-3128* and *hsa-miR-3201.* Bibliographic references about these miRNAs are scarce, and mostly highlight a putative role in cancer or cardiovascular disorders [84, 85] as miRNAs could regulate a broad range of biological processes, like cell cycle, apoptosis, stress response, differentiation, proliferation and angiogenesis among the others [73, 86, 87]. Thus, it is possible that their involvement in such processes will in part explain the well-known neuroprotective and anti-apoptotic effects of Li [44, 77, 78, 88].

Regarding mRNAs potentially targeted by these miRNAs, we found genes previously associated with Li response. Among others, we found Inositol Monophosphatase 2 (IMPA2), which has been implicated in BD and Li response [44, 77, 89, 90], HLA genes that have been recently associated with Li response in BD [63] and Phosphatidylinositol-4,5-Bisphosphate 3-Kinase Catalytic Subunit Gamma (PIK3CG) whose expression has been already found to be modulated by Li treatment in LCLs of patients diagnosed with BD [33].

In order to identify mRNA-miRNA interactions that may play an important role in Li treatment response, we filtered the entire list of significant mature mRNA-miRNA correlations considering the strength of correlation and we obtained 97 mRNA-miRNA pairs. Interestingly, the best pair was represented by hsa-miR-574-3p and RNF125, because it showed the strongest negative correlation coefficient among all mRNA-miRNA interactions.

RNF125, also named T cell RING protein in activation (TRAC-1), is an E3 ubiquitin ligase that contains a RING finger domain in the N-terminus and three zinc-binding and one ubiquitin-interacting motif in the C-terminus. RNF125 protein may function as a positive regulator in the T-cell receptor signalling pathway [91–93]. Activation of T cells results in a pro-inflammatory response necessary to prevent the spread of infection. Limiting T cell signalling, however, is essential to prevent this protective response from causing injury to the host. Findings from our analysis suggest a down-regulation of RNF125, probably mediated by *hsa-miR-574-3p* that could result in a mitigation of immune response.

Therefore, all data seem to converge on the anti-inflammatory effects of Li in BD responders, supporting the hypothesis that pathways associated with cellular immune response could play a biological role in Li treatment response.

We are aware that our findings should be interpreted with caution in light of several limitations. In our study, we are not able to discriminate whether the observed effect is specifically associated with BD or is a general effect of Li treatment response. Indeed, in our dataset we have a few missing data and therefore we cannot provide information about the length of Li treatment. Moreover, we cannot exclude that the lack or the presence of Li treatment may have interfered with the results.

## Conclusions

Taken together, our results strengthen the evidence that Li may act in modulating the inflammatory signaling and immune functions, and provide further evidence for the involvement of miRNAs in Li response. However, future studies should be conducted in larger cohorts of BD subjects by integrating data from different high-throughput approaches to offer a successful strategy for the identification of molecular signatures related both to mechanism of action and efficacy of Li. This would enable the personalization of treatment, define criteria for predicting whether patients diagnosed with BD will respond or not to Li, and improve their long-term management and prognosis, thus reducing the risk for suicidal behaviours.

## Supplementary Information

**Additional file 1 Supplementary Table 1.** List of 335 differentially expressed mRNAs identified in Li responders (R) versus non-responders (NR) with |FC| ≥ 1.2 and *p*-value ≤0.05.

**Additional file 2 Supplementary Table 2.** List of 57 common transcripts between those differentially expressed in our dataset (BD responders (R) versus non-responders (NR)) and other available transcriptomic studies in Pubmed. *: LCLs from Li-responders versus Li non-responders patients diagnosed with BD. #: Li-treated vs vehicle-treated LCLs from patients diagnosed with BD. Δ: before Li-treatment versus after Li-treatment in healthy controls. +: Li-treated vs vehicle-treated LCLs from Li-responders and Li non-responders patients diagnosed with BD. •: Li-treated LCLs versus vehicle-treated LCLs from Li-responders patients diagnosed with BD. □: Li-treated versus vehicle-treated LCLs from healthy controls.

**Additional file 3 Supplementary Table 3.** List of 54 statistically significant pathways modulated by 335 differentially expressed genes identified using IPA. Ratio is calculated as the number of analysis-ready genes in a given pathway, divided by the total number of genes in the reference dataset that makes up that pathway. Z-score predicts the activation state of the canonical pathways, using the gene expression patterns of the genes within the pathway. A negative z value connotes an overall pathway’s inhibition while a positive z value is representative of an overall pathway’s activation. A NaN z-score is assigned to a pathway when gene expression patterns within that pathway are not sufficient to perform the analysis and predict the activation/inhibition state.

**Additional file 4 Supplementary Table 4.** List of 21 relevant functional networks identified by performing a network analysis in IPA on 335 differentially expressed mRNAs.

**Additional file 5 Supplementary Table 5.** List of 77 differentially expressed mature miRNAs identified in Li responders (R) versus non-responders (NR) with |FC| ≥ 1.2 and *p*-value ≤0.05.

**Additional file 6 Supplementary Table 6.** List of 77 statistically significant pathways targeted by differentially modulated miRNAs identified using Diana Tool software (miRPath v.3). MiRNAs and their targeted mRNAs are also reported.

**Additional file 7 Supplementary Table 7.** List of 5450 significant mature miRNA-mRNA interactions with p-value ≤0.05 and Pearson correlation index ρ ≤ − 0.38.

**Additional file 8 Supplementary Table 8.** List of 2027 significant mature miRNA-mRNA interactions with p-value ≤0.05 and Pearson correlation index ρ ≤ − 0.5.

## Data Availability

The datasets used and/or analysed during the current study are available from the corresponding author on reasonable request. We are willing to make the data public, but we are internally setting up the appropriate procedure, and in any case we are happy to share the data with researchers if they are interested, once the manuscript is published.

## Abbreviations

BD: Bipolar disorder
Li: Lithium
GWAS: Genome-wide association study
ConLiGen: International Consortium on Lithium Genetics
lncRNA: long non-coding RNA
miRNAs: microRNAs
mRNA: messenger RNA
LCLs: Lymphoblastoid cell lines
EBV: Epstein-Barr Virus
R: Responders
NR: Non-responders
RMA algorithm: Robust Multi-Array
PCA: Principal Component Analysis
ANOVA: Analysis of variance
IPA: Ingenuity Pathway Analysis
3′-UTRs: 3′-untranslated regions
FDR algorithm: False Discovering Rate
KEGG database: Kyoto Encyclopedia of Genes and Genomes
NF-κB: Nuclear Factor kappa B
Akt/ERK: AKT serine/threonine kinase 1/ extracellular regulated MAP kinase
TNF: Tumor Necrosis Factor
MAP: Molecule Activity Predictor
eQTL: expression Quantitative Trait Loci
FEZ1: Fasciculation and elongation protein zeta
RNF125: Ring Finger protein 125
ELAVL3: ELAV Like RNA Binding Protein 3
CKMT1A: Creatine Kinase, Mitochondrial 1A
KRTAP9–1: Keratin Associated Protein 9–1
HIST3H2A: Histone Cluster 3, H2a
BCAT1: Branched Chain Amino Acid Transaminase 1
RAB11FIP1: RAB11 Family Interacting Protein 1
HLA: Human Leukocyte Antigens
GSK-3β: Glycogen synthase kinase-3β
iPSCs: induced pluripotent stem cells
DNMT1: DNA methyltransferase
IMPA2: Inositol Monophosphatase 2
PIK3CG: Phosphatidylinositol-4,5-Bisphosphate 3-Kinase Catalytic Subunit Gamma
TRAC-1: T cell RING protein in activation
