# Supplementary material for: Transcriptomics and miRNomics data integration in lymphoblastoid cells highlights the key role of immune-related functions in lithium treatment response in Bipolar disorder
Source: BMC Psychiatry. 2022 Oct 27;22:665. doi: 10.1186/s12888-022-04286-3 (PMC9615157; doi:10.1186/s12888-022-04286-3)
Supplement: Supplementary file 1 — Additional file 1 Supplementary Table 1. List of 335 differentially expressed mRNAs identified in Li responders (R) versus non-responders (NR) with |FC| ≥ 1.2 and p-value ≤0.05. [file 12888_2022_4286_MOESM1_ESM.docx]

**Supplementary Table 1.** List of 335 differentially expressed mRNAs identified in Li responders (R) versus non-responders (NR) with |FC| ≥ 1.2 and p-value ≤ 0.05.

|  | **Gene symbol** | **p-value  (R vs. NR)** | **Fold-Change**  **(R vs. NR)** |
| --- | --- | --- | --- |
| 1 | ABCA5 | 4.09E-02 | 1.22 |
| 2 | AGPAT4 | 2.38E-02 | 1.21 |
| 3 | AKAP11-IT1 | 2.81E-02 | 1.26 |
| 4 | ALOX12P2 | 8.92E-03 | 1.52 |
| 5 | ANKRD18A | 3.67E-02 | 1.21 |
| 6 | AP1S3 | 3.33E-02 | 1.32 |
| 7 | AQP1 | 6.59E-03 | -1.21 |
| 8 | AQP7 | 1.30E-02 | 1.27 |
| 9 | ARHGAP26-IT1 | 6.33E-03 | 1.38 |
| 10 | ARHGEF11 | 4.55E-02 | 1.21 |
| 11 | ASNA1 | 2.57E-03 | -1.23 |
| 12 | AUTS2 | 2.77E-02 | 1.51 |
| 13 | BARX2 | 8.81E-03 | 1.22 |
| 14 | BAZ2B | 1.77E-02 | 1.35 |
| 15 | BCAS1 | 1.72E-02 | 1.55 |
| 16 | BCAT1 | 2.73E-02 | 1.37 |
| 17 | BCR | 2.23E-02 | 1.30 |
| 18 | C11orf75 | 4.65E-02 | 1.21 |
| 19 | C12orf63 | 7.66E-03 | 1.54 |
| 20 | C16orf90 | 6.09E-04 | 1.25 |
| 21 | C1orf173 | 1.26E-02 | 1.21 |
| 22 | C1orf38 | 2.68E-03 | 1.20 |
| 23 | C8orf46 | 3.29E-02 | -1.22 |
| 24 | C8orf47 | 2.89E-02 | -1.25 |
| 25 | C8orf74 | 5.57E-03 | -1.23 |
| 26 | CAV1 | 3.84E-02 | 1.22 |
| 27 | CCDC122 | 1.76E-02 | 1.44 |
| 28 | CCHCR1 | 1.51E-02 | 1.24 |
| 29 | CD274 | 8.72E-03 | 1.33 |
| 30 | CD55 | 5.09E-03 | 1.24 |
| 31 | CDKL5 | 2.61E-02 | 1.47 |
| 32 | CDKN2C | 4.35E-02 | -1.26 |
| 33 | CERS6 | 1.25E-02 | -1.30 |
| 34 | CES3 | 1.78E-02 | 1.28 |
| 35 | CGB8 | 3.89E-02 | -1.32 |
| 36 | CHAC2 | 1.76E-03 | -1.23 |
| 37 | CHL1-AS1 | 1.53E-02 | -1.27 |
| 38 | CKMT1A | 2.01E-03 | -1.21 |
| 39 | CLEC2B | 3.78E-02 | 1.49 |
| 40 | CLEC4D | 9.49E-03 | -1.38 |
| 41 | CLEC6A | 5.42E-03 | -1.79 |
| 42 | CLIC6 | 6.71E-03 | 1.47 |
| 43 | CR2 | 2.07E-02 | 1.70 |
| 44 | CRTAP | 1.41E-02 | -1.23 |
| 45 | CTF1 | 5.84E-03 | -1.22 |
| 46 | CTTNBP2 | 1.17E-02 | -1.21 |
| 47 | CXCR2P1 | 7.83E-03 | 1.54 |
| 48 | CXCR5 | 1.06E-02 | 1.28 |
| 49 | CYB5R2 | 4.33E-03 | 1.42 |
| 50 | CYBRD1 | 2.23E-02 | 1.29 |
| 51 | CYP1B1 | 2.27E-02 | 1.45 |
| 52 | CYP3A7-CYP3AP1 | 1.03E-02 | -1.22 |
| 53 | CYTH3 | 5.38E-03 | 1.33 |
| 54 | DNAJB6 | 6.91E-05 | 1.20 |
| 55 | DPYSL2 | 1.98E-02 | -1.21 |
| 56 | DTX1 | 1.80E-02 | 1.34 |
| 57 | EBF1 | 3.95E-02 | 1.21 |
| 58 | EBI3 | 4.16E-03 | 1.31 |
| 59 | EDC4 | 9.09E-03 | 1.32 |
| 60 | ELAVL3 | 5.28E-04 | -1.21 |
| 61 | ELF3 | 1.04E-02 | 1.22 |
| 62 | EN2 | 5.56E-03 | 1.25 |
| 63 | ENPP4 | 2.76E-02 | 1.28 |
| 64 | ESYT1 | 8.15E-04 | -1.21 |
| 65 | ETV3L | 3.53E-03 | 1.21 |
| 66 | F5 | 3.70E-02 | 1.38 |
| 67 | FAM114A1 | 1.94E-02 | 1.33 |
| 68 | FAM165A | 5.17E-03 | -1.25 |
| 69 | FAM174B | 1.57E-02 | 1.27 |
| 70 | FAM213A | 1.14E-02 | 1.21 |
| 71 | FAM25A | 3.05E-04 | -1.24 |
| 72 | FAM27L | 3.32E-02 | -1.26 |
| 73 | FARP1 | 3.02E-02 | 1.54 |
| 74 | FEZ1 | 3.14E-02 | 1.51 |
| 75 | FOXP1 | 3.04E-02 | -1.49 |
| 76 | FXN | 9.11E-03 | -1.21 |
| 77 | G0S2 | 3.36E-02 | 1.32 |
| 78 | GAPT | 1.38E-02 | -1.54 |
| 79 | GAS7 | 9.93E-03 | 1.43 |
| 80 | GBA | 8.30E-03 | -1.21 |
| 81 | GBP3 | 3.80E-02 | -1.27 |
| 82 | GDF11 | 3.15E-02 | -1.26 |
| 83 | GMPR | 4.37E-02 | -1.51 |
| 84 | GOLGA6L5 | 1.67E-02 | 1.31 |
| 85 | GPC5 | 2.27E-02 | 1.28 |
| 86 | GPR137B | 2.20E-02 | 1.22 |
| 87 | GPR141 | 1.82E-02 | 1.33 |
| 88 | GPR150 | 2.91E-03 | 1.27 |
| 89 | GPR171 | 3.29E-02 | -1.54 |
| 90 | GRAMD1B | 3.44E-02 | 1.26 |
| 91 | GRAMD1C | 3.20E-02 | 1.22 |
| 92 | GSTA4 | 2.81E-02 | 1.59 |
| 93 | GTF2H2C | 3.06E-02 | 1.22 |
| 94 | HAL | 7.70E-03 | 1.21 |
| 95 | HDGFRP3 | 2.36E-02 | 1.51 |
| 96 | HECW2 | 1.06E-02 | 2.03 |
| 97 | HHAT | 1.71E-04 | 1.27 |
| 98 | HIGD2A | 3.21E-03 | 1.22 |
| 99 | HIST1H1B | 8.02E-03 | -1.23 |
| 100 | HIST1H1D | 9.13E-03 | -1.24 |
| 101 | HIST1H2BC | 4.69E-02 | -1.29 |
| 102 | HIST1H3G | 1.81E-02 | -1.23 |
| 103 | HIST3H2A | 3.17E-03 | -1.30 |
| 104 | HLA-B | 3.76E-02 | 1.86 |
| 105 | HLA-C | 2.84E-02 | 1.26 |
| 106 | HLA-DRB1 | 4.77E-02 | 6.58 |
| 107 | HLA-DRB5 | 4.52E-02 | 5.88 |
| 108 | HLA-F-AS1 | 4.85E-02 | 1.24 |
| 109 | HMSD | 4.50E-03 | 1.33 |
| 110 | IGKV1D-8 | 1.39E-02 | -1.49 |
| 111 | IGKV2-29 | 4.17E-02 | -2.94 |
| 112 | IGKV2D-29 | 3.05E-02 | -3.25 |
| 113 | IGLV1-44 | 1.43E-02 | -5.00 |
| 114 | IGSF3 | 2.78E-02 | 1.33 |
| 115 | IMPA2 | 1.29E-03 | -1.38 |
| 116 | ISPD | 2.36E-02 | -1.22 |
| 117 | JMY | 1.04E-02 | 1.23 |
| 118 | KCNK12 | 1.01E-02 | 1.23 |
| 119 | KCNMA1 | 6.67E-04 | 1.41 |
| 120 | KIAA1210 | 6.12E-04 | -1.20 |
| 121 | KIAA1522 | 4.59E-03 | 1.22 |
| 122 | KIF21A | 4.69E-02 | 1.26 |
| 123 | KLF3 | 5.67E-03 | 1.57 |
| 124 | KLHL22-IT1 | 3.39E-02 | 1.22 |
| 125 | KRT16P3 | 2.17E-02 | -1.21 |
| 126 | KRT6C | 2.47E-02 | -1.20 |
| 127 | KRTAP10-1 | 1.80E-02 | 1.47 |
| 128 | KRTAP9-1 | 2.13E-02 | -1.20 |
| 129 | LACTB | 2.73E-02 | 1.23 |
| 130 | LAMB1 | 2.48E-02 | -1.31 |
| 131 | LCN12 | 7.10E-03 | -1.20 |
| 132 | LGALS3 | 5.01E-03 | 1.27 |
| 133 | LINC00083 | 2.71E-02 | 1.21 |
| 134 | LINC00891 | 2.73E-02 | -1.24 |
| 135 | LINC01011 | 6.87E-03 | -1.23 |
| 136 | LINC01181 | 3.55E-03 | 1.29 |
| 137 | LMO7 | 3.98E-02 | 1.29 |
| 138 | LOC100034248 | 4.39E-02 | 1.22 |
| 139 | LOC100129534 | 2.85E-02 | 1.21 |
| 140 | LOC100129617 | 2.85E-02 | 1.20 |
| 141 | LOC100130298 | 2.42E-02 | -1.25 |
| 142 | LOC100130476 | 8.87E-03 | 1.23 |
| 143 | LOC100131289 | 1.06E-04 | 1.26 |
| 144 | LOC100131395 | 2.27E-02 | 1.20 |
| 145 | LOC100132314 | 3.81E-03 | -1.24 |
| 146 | LOC100132781 | 1.08E-02 | -1.24 |
| 147 | LOC100216479 | 1.58E-02 | 1.40 |
| 148 | LOC100289637 | 8.59E-03 | 1.23 |
| 149 | LOC100505626 | 4.60E-03 | -1.21 |
| 150 | LOC100506157 | 1.47E-02 | 1.20 |
| 151 | LOC100506571 | 2.63E-03 | -1.21 |
| 152 | LOC100507139 | 1.22E-02 | 1.41 |
| 153 | LOC100509247 | 2.18E-02 | 1.24 |
| 154 | LOC100509457 | 4.00E-03 | 1.24 |
| 155 | LOC100653076 | 2.57E-04 | 1.22 |
| 156 | LOC220729 | 3.91E-03 | 1.20 |
| 157 | LOC253039 | 4.67E-02 | -1.39 |
| 158 | LOC389906 | 9.67E-03 | -1.93 |
| 159 | LOC401321 | 7.89E-03 | 1.26 |
| 160 | LOC441698 | 2.26E-02 | 1.22 |
| 161 | LOC441956 | 6.40E-03 | 1.22 |
| 162 | LOC642838 | 2.91E-02 | -1.66 |
| 163 | LOC643401 | 1.33E-02 | 1.69 |
| 164 | LOC643723 | 5.97E-03 | -1.23 |
| 165 | LOC644189 | 2.47E-02 | 1.21 |
| 166 | LOC730268 | 2.41E-03 | 1.28 |
| 167 | LRRC8B | 7.32E-03 | 1.23 |
| 168 | LUC7L | 3.71E-04 | 1.22 |
| 169 | MAL | 3.91E-02 | 1.89 |
| 170 | MALL | 4.82E-02 | -1.23 |
| 171 | MFN1 | 5.68E-03 | 1.20 |
| 172 | MIR107 | 2.48E-03 | 1.31 |
| 173 | MIR1205 | 1.47E-02 | 1.33 |
| 174 | MIR1229 | 2.59E-02 | 1.23 |
| 175 | MIR1284 | 4.56E-02 | -1.23 |
| 176 | MIR193B | 2.66E-02 | 1.25 |
| 177 | MIR2116 | 4.69E-02 | 1.29 |
| 178 | MIR3162 | 2.63E-02 | 1.25 |
| 179 | MIR3922 | 1.86E-02 | -1.24 |
| 180 | MIR4266 | 3.61E-02 | -1.28 |
| 181 | MIR4299 | 1.14E-02 | -1.21 |
| 182 | MIR4429 | 2.45E-02 | -1.27 |
| 183 | MIR4461 | 1.04E-03 | -1.31 |
| 184 | MIR4509-1 | 2.37E-03 | 1.25 |
| 185 | MIR4530 | 1.97E-02 | -1.39 |
| 186 | MIR4535 | 6.05E-03 | -1.25 |
| 187 | MIR4640 | 5.20E-03 | 1.20 |
| 188 | MIR4660 | 4.84E-02 | -1.20 |
| 189 | MIR4742 | 3.62E-03 | 1.76 |
| 190 | MIR500A | 3.22E-02 | -1.21 |
| 191 | MIR516B2 | 1.61E-02 | -1.20 |
| 192 | MIR521-2 | 1.32E-02 | -1.22 |
| 193 | MIR548A2 | 1.56E-02 | 1.25 |
| 194 | MIR548B | 2.62E-02 | -1.22 |
| 195 | MIR550A1 | 3.83E-02 | 1.24 |
| 196 | MIR579 | 2.61E-02 | 1.26 |
| 197 | MIR744 | 2.33E-02 | 1.25 |
| 198 | MIR877 | 1.94E-02 | 1.26 |
| 199 | MNX1 | 2.81E-02 | -1.20 |
| 200 | MRPL33 | 2.99E-02 | -1.20 |
| 201 | MYT1 | 4.66E-03 | 1.21 |
| 202 | NAALADL2-AS2 | 2.39E-03 | 2.28 |
| 203 | NFIC | 1.79E-03 | -1.22 |
| 204 | NFKBIZ | 4.66E-02 | 1.27 |
| 205 | NIPSNAP3B | 9.14E-03 | 1.49 |
| 206 | NLRP1 | 4.13E-02 | 1.25 |
| 207 | NR1D1 | 1.16E-02 | 1.24 |
| 208 | NRG4 | 3.09E-02 | 1.29 |
| 209 | NRXN3 | 2.84E-02 | 1.22 |
| 210 | NSMCE1-DT | 1.60E-02 | 1.20 |
| 211 | NT5DC3 | 4.07E-03 | 1.22 |
| 212 | NUAK1 | 3.17E-03 | 1.24 |
| 213 | NYAP1 | 4.54E-03 | -1.22 |
| 214 | OR2A1 | 4.11E-02 | 1.41 |
| 215 | OR2L3 | 2.96E-02 | 1.25 |
| 216 | OSCAR | 7.93E-03 | 1.22 |
| 217 | PDX1 | 2.43E-02 | 1.23 |
| 218 | PEG10 | 3.05E-02 | -1.35 |
| 219 | PFN4 | 1.18E-04 | 1.24 |
| 220 | PGAM1 | 4.68E-02 | 1.22 |
| 221 | PIK3CG | 4.96E-02 | -1.28 |
| 222 | PLEKHF2 | 3.13E-02 | 1.22 |
| 223 | PMCHL1 | 9.71E-03 | -1.25 |
| 224 | PPAPDC1B | 8.33E-03 | 1.26 |
| 225 | PPM1A | 4.16E-03 | 1.25 |
| 226 | PPP1R32 | 2.34E-03 | 1.22 |
| 227 | PRAMEF22 | 1.20E-02 | -1.28 |
| 228 | PRF1 | 6.62E-03 | 1.20 |
| 229 | PRKCI | 1.10E-02 | -1.25 |
| 230 | PRR5L | 2.82E-03 | -1.23 |
| 231 | PYCARD | 3.17E-02 | -1.22 |
| 232 | RAB11FIP1 | 3.57E-02 | 1.21 |
| 233 | RAB39A | 2.14E-02 | 1.36 |
| 234 | RAB42 | 1.04E-02 | -1.39 |
| 235 | RASGEF1A | 6.59E-04 | -1.34 |
| 236 | RHOU | 3.01E-02 | -1.49 |
| 237 | RN5S128 | 2.38E-03 | -1.41 |
| 238 | RN5S134 | 4.02E-02 | 1.38 |
| 239 | RN5S158 | 3.41E-02 | 1.32 |
| 240 | RN5S177 | 1.96E-02 | 1.29 |
| 241 | RN5S208 | 9.38E-04 | 1.22 |
| 242 | RN5S309 | 3.47E-02 | 1.22 |
| 243 | RN5S325 | 5.13E-03 | 1.52 |
| 244 | RN5S358 | 1.20E-02 | -1.25 |
| 245 | RN5S387 | 2.22E-02 | 1.34 |
| 246 | RN5S388 | 2.18E-02 | -1.24 |
| 247 | RN5S431 | 4.13E-02 | -1.21 |
| 248 | RN5S448 | 2.99E-03 | 1.23 |
| 249 | RNF125 | 1.88E-03 | -1.56 |
| 250 | RNU1-13P | 3.68E-02 | -1.31 |
| 251 | RNU4ATAC3P | 2.55E-02 | 1.21 |
| 252 | RNU6-66 | 3.77E-03 | 1.30 |
| 253 | RNU6-82 | 4.22E-02 | 1.23 |
| 254 | RNU7-19P | 4.32E-03 | 1.22 |
| 255 | RNU7-24P | 1.16E-02 | 1.35 |
| 256 | RNU7-2P | 6.74E-03 | -1.44 |
| 257 | RNU7-35P | 2.40E-02 | 1.21 |
| 258 | RORA | 1.36E-02 | 1.40 |
| 259 | RPL23AP32 | 3.40E-02 | 1.68 |
| 260 | S100A6 | 2.90E-02 | 1.23 |
| 261 | SAMD12 | 3.77E-02 | -1.72 |
| 262 | SCARNA1 | 2.11E-02 | -1.21 |
| 263 | SERPINA1 | 3.66E-02 | 1.21 |
| 264 | SERPINA9 | 9.49E-03 | 1.27 |
| 265 | SERPINB10 | 1.92E-02 | 2.31 |
| 266 | SLC12A8 | 1.40E-02 | 1.30 |
| 267 | SLC35G5 | 6.72E-03 | 1.28 |
| 268 | SLC44A5 | 2.16E-02 | 1.96 |
| 269 | SLC7A7 | 1.60E-02 | 1.28 |
| 270 | SNORA33 | 1.47E-03 | 1.23 |
| 271 | SNORA38B | 1.15E-02 | 1.59 |
| 272 | SNORD114-31 | 1.56E-02 | -1.24 |
| 273 | SNORD37 | 4.28E-02 | 1.31 |
| 274 | SNRPN | 2.69E-02 | -1.27 |
| 275 | SPC25 | 2.66E-03 | -1.26 |
| 276 | SPRED2 | 1.21E-02 | -1.20 |
| 277 | SPRYD4 | 4.80E-02 | -1.20 |
| 278 | ST3GAL1 | 6.14E-03 | -1.23 |
| 279 | ST6GALNAC2 | 1.10E-02 | 1.34 |
| 280 | STK39 | 5.23E-05 | -1.20 |
| 281 | SULT1A1 | 2.95E-03 | -1.30 |
| 282 | SUMO4 | 1.08E-02 | 1.20 |
| 283 | SYCP2L | 4.35E-02 | -1.37 |
| 284 | SYNPO | 1.09E-02 | 1.22 |
| 285 | TAS2R19 | 4.30E-02 | 1.34 |
| 286 | TAS2R31 | 3.08E-02 | 1.32 |
| 287 | TBC1D12 | 8.49E-03 | 1.21 |
| 288 | TBC1D25 | 6.41E-03 | -1.26 |
| 289 | TBX22 | 2.68E-02 | -1.22 |
| 290 | TC2N | 2.43E-02 | 1.59 |
| 291 | TCFL5 | 1.71E-02 | 1.33 |
| 292 | TLR10 | 2.91E-02 | 1.23 |
| 293 | TLR6 | 1.28E-02 | 1.40 |
| 294 | TLR9 | 1.45E-02 | -1.21 |
| 295 | TNFRSF14 | 3.92E-02 | 1.26 |
| 296 | TPI1P3 | 1.39E-02 | -1.21 |
| 297 | TRAJ21 | 4.73E-02 | 1.36 |
| 298 | TRAJ24 | 4.26E-02 | 1.32 |
| 299 | TRAJ36 | 3.06E-02 | 1.47 |
| 300 | TRAJ38 | 2.32E-02 | 1.49 |
| 301 | TRAJ41 | 2.79E-02 | 1.38 |
| 302 | TRAJ42 | 4.37E-02 | 1.35 |
| 303 | TRAJ43 | 2.76E-02 | 1.31 |
| 304 | TRAJ44 | 4.03E-02 | 1.49 |
| 305 | TRAJ45 | 1.84E-02 | 1.62 |
| 306 | TRAJ47 | 4.64E-02 | 1.38 |
| 307 | TRAJ50 | 2.15E-02 | 1.24 |
| 308 | TRAJ52 | 2.61E-03 | 1.89 |
| 309 | TRAJ54 | 4.88E-02 | 1.33 |
| 310 | TRBV21OR9-2 | 1.60E-02 | 1.20 |
| 311 | TRBV23-1 | 4.84E-02 | -1.21 |
| 312 | TRBV4-2 | 4.27E-03 | -1.26 |
| 313 | TREX2 | 1.22E-03 | 1.44 |
| 314 | TRGV2 | 4.85E-02 | -1.25 |
| 315 | TRIM2 | 3.56E-03 | 1.32 |
| 316 | TRIM53AP | 2.04E-02 | 1.22 |
| 317 | TSPAN11 | 1.31E-02 | -1.21 |
| 318 | TUBA8 | 4.96E-02 | 1.32 |
| 319 | TUBB2B | 1.09E-02 | 1.44 |
| 320 | UBE2Q2P1 | 2.04E-03 | -1.24 |
| 321 | UBE2QL1 | 1.40E-02 | 1.20 |
| 322 | ULK2 | 2.08E-02 | 1.28 |
| 323 | XXYLT1-AS1 | 2.24E-02 | 1.33 |
| 324 | ZBTB20 | 1.21E-02 | 1.22 |
| 325 | ZBTB32 | 3.24E-02 | 1.24 |
| 326 | ZC2HC1B | 4.06E-03 | -1.41 |
| 327 | ZC3H12C | 4.19E-02 | 1.31 |
| 328 | ZG16B | 8.01E-04 | 1.59 |
| 329 | ZNF165 | 6.10E-03 | 1.26 |
| 330 | ZNF382 | 3.03E-02 | 1.23 |
| 331 | ZNF416 | 5.87E-03 | -1.22 |
| 332 | ZNF487P | 2.36E-04 | -1.22 |
| 333 | ZNF492 | 5.90E-03 | -1.24 |
| 334 | ZNF608 | 4.82E-02 | 1.70 |
| 335 | ZNF765 | 3.91E-03 | 1.25 |
