# Supplementary material for: Transcriptomics and miRNomics data integration in lymphoblastoid cells highlights the key role of immune-related functions in lithium treatment response in Bipolar disorder
Source: BMC Psychiatry. 2022 Oct 27;22:665. doi: 10.1186/s12888-022-04286-3 (PMC9615157; doi:10.1186/s12888-022-04286-3)
Supplement: Supplementary file 2 — Additional file 2 Supplementary Table 2. List of 57 common transcripts between those differentially expressed in our dataset (BD responders (R) versus non-responders (NR)) and other available transcriptomic studies in Pubmed. *: LCLs from Li-responders versus Li non-responders patients diagnosed with BD. #: Li-treated vs vehicle-treated LCLs from patients diagnosed with BD. Δ: before Li-treatment versus after Li-treatment in healthy controls. +: Li-treated vs vehicle-treated LCLs from Li-responders and Li non-responders patients diagnosed with BD. •: Li-treated LCLs versus vehicle-treated LCLs from Li-responders patients diagnosed with BD. □: Li-treated versus vehicle-treated LCLs from healthy controls. [file 12888_2022_4286_MOESM2_ESM.docx]

**Supplementary Table 2.** List of 42 common transcripts between those differentially expressed in our dataset (BD responders (R) versus non-responders (NR)) and other available transcriptomic studies in Pubmed.

|  | **GENE** | **Fold-Change observed in our data set**  **(R vs NR)** | **REFERENCES** |
| --- | --- | --- | --- |
| 1 | ALOX12P2 | 1.52 | *Milanesi et al., 2019* * |
| 2 | AUTS2 | 1.51 | *Kittel-Schneider et al., 2019* # |
| 3 | BCAT1 | 1.37 | *Milanesi et al., 2019* *; *Fries et al., 2017* #; *Kittel-Schneider et al., 2019* # |
| 4 | C11orf75 | 1.21 | *Kittel-Schneider et al., 2019* # |
| 5 | C8orf47 | -1.25 | *Kittel-Schneider et al., 2019* # |
| 6 | CAV1 | 1.22 | *Fries et al., 2017* # |
| 7 | CD274 | 1.33 | *Watanabe et al., 2014* Δ |
| 8 | CHAC2 | -1.23 | *Kittel-Schneider et al., 2019* # |
| 9 | CR2 | 1.70 | *Squassina et al., 2013* *; *Kittel-Schneider et al., 2019* # |
| 10 | CYP1B1 | 1.45 | *Breen et al., 2016* +; *Kittel-Schneider et al., 2019* # |
| 11 | CYTH3 | 1.33 | *Kittel-Schneider et al., 2019* # |
| 12 | DTX1 | 1.34 | *Breen et al., 2016* + |
| 13 | ENPP4 | 1.28 | *Breen et al., 2016* +; *Kittel-Schneider et al., 2019* # |
| 14 | FAM174B | 1.27 | *Kittel-Schneider et al., 2019* # |
| 15 | FXN | -1.21 | *Kittel-Schneider et al., 2019* # |
| 16 | GAS7 | 1.43 | *Breen et al., 2016* + |
| 17 | GSTA4 | 1.59 | *Breen et al., 2016* + |
| 18 | HDGFRP3 | 1.51 | *Milanesi et al., 2019* * |
| 19 | HECW2 | 2.03 | *Kittel-Schneider et al., 2019* # |
| 20 | IMPA2 | -1.38 | *Kittel-Schneider et al., 2019* # |
| 21 | JMY | 1.23 | *Fries et al., 2017* # |
| 22 | KCNMA1 | 1.41 | *Fries et al., 2017* # |
| 23 | KLF3 | 1.57 | *Kittel-Schneider et al., 2019* # |
| 24 | LGALS3 | 1.27 | *Breen et al., 2016* + |
| 25 | LMO7 | 1.29 | *Hunsberger et al., 2015* • |
| 26 | MAL | 1.89 | *Kittel-Schneider et al., 2019* # |
| 27 | MYT1 | 1.21 | *Hunsberger et al., 2015* • |
| 28 | NFKBIZ | 1.27 | *Milanesi et al., 2019* * |
| 29 | PEG10 | -1.35 | *Breen et al., 2016* + |
| 30 | PPM1A | 1.25 | *Kittel-Schneider et al., 2019* # |
| 31 | PYCARD | -1.22 | *Kittel-Schneider et al., 2019* # |
| 32 | RAB11FIP1 | 1.21 | *Breen et al., 2016* +; *Fries et al., 2017* #; *Kittel-Schneider et al., 2019* # |
| 33 | RNF125 | -1.56 | *Watanabe et al., 2014* Δ |
| 34 | RORA | 1.40 | *Kittel-Schneider et al., 2019* # |
| 35 | S100A6 | 1.23 | *Fries et al., 2017* # |
| 36 | SERPINB10 | 2.31 | *Kittel-Schneider et al., 2019* # |
| 37 | SLC7A7 | 1.28 | *Sugawara et al., 2010* □ |
| 38 | TLR6 | 1.40 | *Kittel-Schneider et al., 2019* # |
| 39 | TLR9 | -1.21 | *Milanesi et al., 2019* * |
| 40 | TRIM2 | 1.32 | *Hunsberger et al., 2015* • |
| 41 | TUBA8 | 1.32 | *Breen et al., 2016* + |
| 42 | UBE2QL1 | 1.20 | *Kittel-Schneider et al., 2019* # |

**Gene expression comparisons in referenced transcriptomic studies**

* : LCLs from Li-responders versus Li non-responders patients diagnosed with BD

# : Li-treated vs vehicle-treated LCLs from patients diagnosed with BD

Δ : before Li-treatment versus after Li-treatment in healthy controls

+ : Li-treated vs vehicle-treated LCLs from Li-responders and Li non-responders patients diagnosed with BD

• : Li-treated LCLs versus vehicle-treated LCLs from Li-responders patients diagnosed with BD

□ : Li-treated versus vehicle-treated LCLs from healthy controls
