# Supplementary material for: Transcriptomics and miRNomics data integration in lymphoblastoid cells highlights the key role of immune-related functions in lithium treatment response in Bipolar disorder
Source: BMC Psychiatry. 2022 Oct 27;22:665. doi: 10.1186/s12888-022-04286-3 (PMC9615157; doi:10.1186/s12888-022-04286-3)
Supplement: Supplementary file 4 — Additional file 4 Supplementary Table 4. List of 21 relevant functional networks identified by performing a network analysis in IPA on 335 differentially expressed mRNAs. [file 12888_2022_4286_MOESM4_ESM.docx]

**Supplementary Table 4.** List of 21 relevant functional networks identified by performing a network analysis in IPA on 335 differentially expressed mRNAs.

| **ID** | **Molecules in the Network** | **Score** | **Focus Molecules** | **Top Diseases and Functions** |
| --- | --- | --- | --- | --- |
| 1 | AUTS2, BCR, BCR(complex), CD274, CD55, CR2, CXCR5, CYP1B1, EBI3, ELF3, FXN, G0S2, GBA, HLA-B, HLA-C, HLA-DRB1, Ige, Immunoglobulin, Interferon alpha, MAL, mir-515, NFkB (complex), NFKBIZ, NLRP1, OSCAR, P38 MAPK, PRKCI, PYCARD, RNA polymerase II, SERPINA1, ST3GAL1, SUMO4, TLR10, TLR6, TLR9 | 49 | 28 | Gastrointestinal Disease, Hepatic System Disease, Liver Damage |
| 2 | Akt, CAV1, CD3, CGB3 (includes others), CLEC2B, CSPG4, DPYSL2, ERK, ERK1/2, F5, FSHR, GPR183, Histone h3, HMGB1, Hsp27, Hsp90, JMY, Jnk, KIR3DL1, LGALS3, MAFA, MAP3K10, mir-103, MYT1, PDX1, PGAM1, PIK3CG, PRF1, S100A6, STK39, SYNPO, TCR, TDGF1, TUBA8, ZBTB32 | 26 | 18 | Cell Signaling, Nucleic Acid Metabolism, Small Molecule Biochemistry |
| 3 | CCDC134, CCHCR1, CD2, CD244, CD274, CD48, CD5, CD80, CRHR1, DUSP10, EBI3, EN2, ENPP2, FEZ1, FOXL2, G0S2, GAS7, HLA-B, IFNG, IL13, IL9, KCNMA1, NFATC2, NUAK1, OSMR, RFX5, SERPINB10, SLC7A7, STAT3, TCFL5, THEMIS2, TLR6, TNF, USP2, ZNF165 | 22 | 16 | Cell Signaling, Nucleic Acid Metabolism, Small Molecule Biochemistry |
| 4 | ACSL5, AGTR1, ATR, BRD7, CDK9, CERS6, Ck2, CYTH3, DICER1, EBF1, ETS2, GBP3, GZMB, H2AFX, HIPK2, HNRNPA2B1, IGKV1D-8, mir-1205, mir-15, mir-155, miR-22-3p (miRNAs w/seed AGCUGCC), mir-548, NAMPT, NFIC, PEG10, PPM1A, PRDM5, RB1CC1, SERPINA1, SNORA33, SULT1A1, TP53, ULK2, VCAN, YWHAH | 18 | 14 | Cancer, Hematological Disease, Immunological Disease |
| 5 | ACACA, ACLY, Actin, ANK1, ATF3, ATR, BBC3, BRCA1, CDC25C, CENPC, ELK1, FAM114A1, FAM213A, FASN, GPC5, GSTA4, H2AFX, HAL, HIST1H1B, HNF1A, HNF4A, KAT5, KLF3, LMO7, MAPK9, miR-483-3p (miRNAs w/seed CACUCCU), NR1D1, NT5DC3, NUPR1, PARP1, PRR5L, RORA, SIRT1, SPC24, SPC25 | 17 | 13 | Cell Death and Survival, DNA Replication, Recombination, and Repair, Lipid Metabolism |
| 6 | APP, AQP1, ARHGEF11, BCAT1, CCND1, CDKN2C, CLIC6, CRTAP, CTTNBP2, CTTNBP2NL, Dynamin, ENPP2, FOXP1, GCSAM, GRB2, HIST1H2BC, IL2, mir-193, mir-320, mir-486, miR-486-5p (and other miRNAs w/seed CCUGUAC), mir-500, mir-744, MOB4, PPP2CA, SPRED2, SSB, STRIP1, STRN, STRN3, STRN4, SYK, SYVN1, VAV, WNT1 | 17 | 13 | Cell Morphology, Cellular Assembly and Organization, Inflammatory Disease |
| 7 | 26s Proteasome, BARX2, CAV2, CDKL5, CDKN3, CENPA, CTCF, DTX1, DYNLL1, ELF1, ENPP4, ERBB2, ESR1, FOXA1, GDF11, HDAC3, HIPK2, HIST1H1D, HIST3H2A, KRT6C, LACTB, LAMP1, MALL, MECP2, MFN1, MYO10, N-cor, NCOA3, OGA, PINK1, RB1, SYCP2L, TFF3, TRIM2, TYMS | 17 | 13 | Cell Cycle, Cellular Assembly and Organization, Cellular Movement |
| 8 | ABCA5, CANX, CAV1, CD44, CST5, CYBRD1, DNAJB6, EDC4, ESYT1, FYN, GMPR, HIF1A, IGSF3, ITGB4, LAMB1, MMP7, MNX1, NPM1, OSM, PMAIP1, PRL, RB1CC1, RPTOR, SBDS, SERPINA1, SMAD2, SNAI2, SPP1, STAT2, TP73, TRPC1, TWIST1, TXN, UBE2QL1, ZBTB20 | 17 | 13 | Cellular Movement, Cellular Development, Cellular Growth and Proliferation |
| 9 | ACSL5, AREG, ARFGEF1, ARRDC4, ATF3, AZGP1, BRD4, CCL3, CSF1, CTSH, CXCL2, CYB5R2, EGR1, ETS2, FOS, HECW2, ICAM1, IL23A, INHBA, IRF1, ITGA4, ITGA5, ITGB1, KANK1, KIF21A, PLEKHF2, RAB11FIP1, RHOU, RNF125, SMARCA4, SPP1, TIMP1, TNFRSF14, TNFSF14, TREM1 | 8 | 8 | Hematological System Development and Function, Immune Cell Trafficking, Inflammatory Response |
| 10 | AGPAT4, HR | 2 | 1 | Dermatological Diseases and Conditions, Developmental Disorder, Embryonic Development |
| 11 | EIF3E, FARP1 | 2 | 1 | Cellular Growth and Proliferation, Organismal Development, Cell Cycle |
| 12 | SNRPB, SNRPN | 2 | 1 | Connective Tissue Disorders, Developmental Disorder, Gastrointestinal Disease |
| 13 | ASNA1, CAMLG | 2 | 1 | Cell Death and Survival, Hematological System Development and Function, Cellular Function and Maintenance |
| 14 | F2, TC2N | 2 | 1 | Carbohydrate Metabolism, Cardiovascular Disease, Cell Cycle |
| 15 | BCAS1, NCOA2 | 2 | 1 | Lipid Metabolism, Molecular Transport, Small Molecule Biochemistry |
| 16 | GLI1, IMPA2 | 2 | 1 | Cancer, Cell Death and Survival, Embryonic Development |
| 17 | PDLIM2, RASGEF1A | 2 | 1 | Cell Morphology, Cellular Assembly and Organization, Cell-To-Cell Signaling and Interaction |
| 18 | CIP2A, SLC12A8 | 2 | 1 | Cell-To-Cell Signaling and Interaction, Cancer, Gastrointestinal Disease |
| 19 | MIR3162, RNA polymerase iii | 2 | 1 | Connective Tissue Disorders, Hematological Disease, Immunological Disease |
| 20 | CKMT1A/CKMT1B, EPAS1 | 2 | 1 | Cell Morphology, Cellular Assembly and Organization, Cancer |
| 21 | FOXQ1, NRXN3, ZNF217 | 1 | 1 | Cellular Development, Cardiovascular System Development and Function, Cellular Growth and Proliferation |
