# Supplementary material for: Transcriptomics and miRNomics data integration in lymphoblastoid cells highlights the key role of immune-related functions in lithium treatment response in Bipolar disorder
Source: BMC Psychiatry. 2022 Oct 27;22:665. doi: 10.1186/s12888-022-04286-3 (PMC9615157; doi:10.1186/s12888-022-04286-3)
Supplement: Supplementary file 5 — Additional file 5 Supplementary Table 5. List of 77 differentially expressed mature miRNAs identified in Li responders (R) versus non-responders (NR) with |FC| ≥ 1.2 and p-value ≤0.05. [file 12888_2022_4286_MOESM5_ESM.docx]

**Supplementary Table 5.** List of 77 differentially expressed mature miRNAs identified in Li responders (R) versus non-responders (NR) with |FC| ≥ 1.2 and p-value ≤ 0.05.

|  | **miRNA** | **p-value**  **(R vs. NR)** | **Fold-Change**  **(R vs. NR)** |
| --- | --- | --- | --- |
| 1 | hsa-miR-3128 | 3.22E-05 | 3.78 |
| 2 | hsa-miR-3201 | 1.58E-05 | 2.75 |
| 3 | hsa-miR-3613-3p | 4.25E-04 | 2.30 |
| 4 | hsa-miR-8084 | 5.00E-03 | 2.04 |
| 5 | hsa-miR-4423-3p | 3.33E-04 | 1.98 |
| 6 | hsa-miR-4445-3p | 4.86E-03 | 1.82 |
| 7 | hsa-miR-574-3p | 4.82E-06 | 1.78 |
| 8 | hsa-miR-98-5p | 6.25E-03 | 1.73 |
| 9 | hsa-miR-4668-5p | 1.69E-05 | 1.72 |
| 10 | hsa-miR-3148 | 1.62E-02 | 1.64 |
| 11 | hsa-miR-152-3p | 6.68E-03 | 1.54 |
| 12 | hsa-miR-335-5p | 3.82E-02 | 1.51 |
| 13 | hsa-miR-4448 | 3.59E-02 | 1.49 |
| 14 | hsa-miR-885-3p | 2.82E-02 | 1.49 |
| 15 | hsa-miR-29c-3p | 3.83E-02 | 1.47 |
| 16 | hsa-miR-204-3p | 1.36E-02 | 1.46 |
| 17 | hsa-miR-6787-5p | 7.19E-04 | 1.46 |
| 18 | hsa-miR-4785 | 7.57E-04 | 1.42 |
| 19 | hsa-miR-6813-5p | 1.93E-02 | 1.40 |
| 20 | hsa-miR-342-5p | 3.16E-03 | 1.37 |
| 21 | hsa-miR-6837-5p | 2.22E-02 | 1.36 |
| 22 | hsa-miR-6830-5p | 9.62E-04 | 1.36 |
| 23 | hsa-miR-4750-5p | 2.06E-02 | 1.35 |
| 24 | hsa-miR-548a-3p | 3.13E-02 | 1.34 |
| 25 | hsa-miR-1468-3p | 8.42E-03 | 1.34 |
| 26 | hsa-miR-3609 | 3.89E-02 | 1.33 |
| 27 | hsa-miR-330-5p | 4.04E-02 | 1.32 |
| 28 | hsa-miR-1260b | 3.16E-02 | 1.32 |
| 29 | hsa-miR-6785-5p | 4.33E-02 | 1.31 |
| 30 | hsa-miR-4446-3p | 3.88E-02 | 1.29 |
| 31 | hsa-miR-3175 | 2.18E-02 | 1.28 |
| 32 | hsa-miR-34a-5p | 3.78E-02 | 1.26 |
| 33 | hsa-miR-4720-5p | 4.05E-03 | 1.26 |
| 34 | hsa-miR-548ac | 2.13E-02 | 1.25 |
| 35 | hsa-miR-579-5p | 9.66E-03 | 1.25 |
| 36 | hsa-miR-664b-3p | 3.92E-02 | 1.23 |
| 37 | hsa-miR-27a-3p | 3.28E-02 | 1.23 |
| 38 | hsa-miR-6825-5p | 8.68E-03 | 1.23 |
| 39 | hsa-miR-635 | 1.59E-03 | 1.23 |
| 40 | hsa-miR-659-3p | 1.68E-02 | 1.22 |
| 41 | hsa-let-7f-1-3p | 1.62E-02 | 1.22 |
| 42 | hsa-miR-6867-5p | 8.06E-03 | 1.22 |
| 43 | hsa-miR-4439 | 6.70E-03 | 1.22 |
| 44 | hsa-miR-5100 | 4.11E-02 | 1.21 |
| 45 | hsa-miR-550a-3-5p | 1.24E-02 | 1.21 |
| 46 | hsa-miR-548x-3p | 2.05E-02 | 1.20 |
| 47 | hsa-miR-3613-5p | 9.19E-03 | -1.20 |
| 48 | hsa-miR-1208 | 7.79E-03 | -1.21 |
| 49 | hsa-miR-3605-3p | 6.08E-03 | -1.21 |
| 50 | hsa-miR-3194-3p | 1.02E-03 | -1.21 |
| 51 | hsa-miR-6507-3p | 2.27E-02 | -1.22 |
| 52 | hsa-miR-296-5p | 2.03E-02 | -1.22 |
| 53 | hsa-miR-4663 | 2.23E-02 | -1.24 |
| 54 | hsa-miR-4270 | 1.55E-02 | -1.24 |
| 55 | hsa-miR-6746-5p | 1.70E-02 | -1.28 |
| 56 | hsa-miR-196b-3p | 1.70E-02 | -1.28 |
| 57 | hsa-miR-5189-3p | 6.76E-03 | -1.29 |
| 58 | hsa-miR-7-5p | 4.74E-03 | -1.29 |
| 59 | hsa-miR-5088-5p | 5.70E-03 | -1.33 |
| 60 | hsa-miR-3689f | 1.79E-02 | -1.35 |
| 61 | hsa-miR-101-5p | 7.33E-03 | -1.36 |
| 62 | hsa-miR-574-5p | 2.25E-02 | -1.37 |
| 63 | hsa-miR-16-2-3p | 4.95E-02 | -1.38 |
| 64 | hsa-miR-195-3p | 8.45E-03 | -1.38 |
| 65 | hsa-miR-4635 | 6.36E-03 | -1.38 |
| 66 | hsa-miR-629-3p | 3.22E-02 | -1.40 |
| 67 | hsa-miR-181a-3p | 3.16E-02 | -1.41 |
| 68 | hsa-miR-4462 | 3.55E-02 | -1.42 |
| 69 | hsa-miR-7-1-3p | 4.09E-02 | -1.43 |
| 70 | hsa-miR-6886-5p | 4.82E-02 | -1.44 |
| 71 | hsa-miR-371b-5p | 2.26E-02 | -1.46 |
| 72 | hsa-miR-6774-5p | 7.69E-03 | -1.49 |
| 73 | hsa-miR-338-5p | 3.79E-02 | -1.50 |
| 74 | hsa-miR-6744-5p | 2.98E-02 | -1.51 |
| 75 | hsa-miR-4518 | 2.04E-02 | -1.53 |
| 76 | hsa-miR-8063 | 3.63E-02 | -1.56 |
| 77 | hsa-miR-6893-5p | 2.27E-02 | -1.63 |
