# Supplementary material for: Transcriptomics and miRNomics data integration in lymphoblastoid cells highlights the key role of immune-related functions in lithium treatment response in Bipolar disorder
Source: BMC Psychiatry. 2022 Oct 27;22:665. doi: 10.1186/s12888-022-04286-3 (PMC9615157; doi:10.1186/s12888-022-04286-3)
Supplement: Supplementary file 6 — Additional file 6 Supplementary Table 6. List of 77 statistically significant pathways targeted by differentially modulated miRNAs identified using Diana Tool software (miRPath v.3). MiRNAs and their targeted mRNAs are also reported. [file 12888_2022_4286_MOESM6_ESM.docx]

**Supplementary Table 6.** List of 77 statistically significant pathways targeted by differentially modulated miRNAs identified using Diana Tool software (miRPath v.3). MiRNAs and their targeted mRNAs are also reported.

| **KEGG pathway** | **Genes** | **miRNAs** |
| --- | --- | --- |
| **Mucin type O-Glycan biosynthesis** | POC1B-GALNT4, GALNT7, GALNTL6, GALNT15, B4GALT5, GALNTL5, GCNT4, GALNT11, ST3GAL1, GALNT18, GALNT13, GALNT4, GALNT6, ST3GAL2, GCNT3, GALNT1, GALNT3, GALNT14, GALNT10, WBSCR17, GALNT2, C1GALT1, GCNT1, C1GALT1C1, GALNT12, GALNT5, GALNT16 | hsa-miR-1468-3p, hsa-miR-3613-3p, hsa-miR-98-5p, hsa-miR-3148, hsa-miR-548a-3p,  hsa-miR-548ac, hsa-miR-16-2-3p, hsa-miR-195-3p, hsa-miR-7-1-3p,  hsa-miR-548x-3p, hsa-miR-4720-5p, hsa-miR-6785-f5p, hsa-miR-3609, hsa-miR-8063,  hsa-miR-6893-5p, hsa-miR-4439, hsa-miR-550a-3-5p, hsa-miR-4446-3p, hsa-miR-6867-5p, hsa-miR-342-5p, hsa-miR-635, hsa-miR-6774-5p, hsa-miR-152-3p, hsa-miR-659-3p,  hsa-miR-5189-3p, hsa-miR-4668-5p, hsa-miR-296-5p, hsa-miR-664b-3p, hsa-miR-27a-3p, hsa-miR-204-3p, hsa-miR-6825-5p, hsa-miR-338-5p, hsa-miR-629-3p, hsa-miR-6787-5p,  hsa-miR-7-5p, hsa-miR-574-5p, hsa-miR-3689f, hsa-miR-8084, hsa-miR-34a-5p,  hsa-let-7f-1-3p, hsa-miR-29c-3p |
| **Proteoglycans in cancer** | FZD7, ESR1, CAMK2D, BRAF, ACTB, TLR2, HBEGF, PRKCA, STAT3, FZD5, PDCD4, ERBB2, MET, ITGB1, WNT7A, EZR, ROCK1, SOS2, FLNC, SMAD2, CBL, SDC1, NRAS, THBS1, PIK3CB, PTCH1, WNT10B, CAV1, WNT5A, ARHGEF12, PIK3R5, PPP1CC, MAPK14, ACTG1, PXN, ROCK2, FRS2, FZD6, RDX, IQGAP1, ITGA5, RAF1, WNT2B, WNT4, TIAM1, WNT3, IGF1R, EGFR, MRAS, TLR4, RHOA, PPP1R12B, CAV2, ERBB3, FZD8, KRAS, TGFB1, TWIST2, FZD3, RRAS2, FASLG, MSN, ANK2, RPS6KB2, ARHGEF1, PAK1, COL21A1, HSPG2, HPSE, CTTN, VAV2, CAMK2A, AKT2, PTK2, CBLB, ITGAV, ANK3, HCLS1, SLC9A1, FZD4, NUDT16L1, TFAP4, PPP1R12A, DROSHA, MMP2, DCN, PLCG1, CASP3, PIK3CD, PIK3R3, PTPN6, CCND1, CTNNB1, HIF1A, AKT1, MYC, IGF2, FLNB, PRKACG, PRKACA, EIF4B, TIMP3, FLNA, ITGA2, ITPR1, PIK3R1, SOS1, PTPN11, PRKX, CTSL, DDX5, RAC1, MAPK3, FGF2, CDC42, LUM, PLCG2, FZD1, PRKCB, FAS, IGF1, TGFB2, GAB1, WNT10A, ANK1, AKT3, CAMK2B, PLCE1, NANOG, PIK3CA, SDC2, HOXD10, FN1, CDKN1A, MAP2K1, SDC4, SMO, PDPK1, WNT2, VMP1, HGF, WNT9B, MTOR, TWIST1, TNF, ITPR3, VEGFA, FGFR1, MAPK1, ITPR2, KDR, GRB2, WNT7B, CD63, MDM2, ELK1, WNT9A, ERBB4, RRAS, RPS6KB1, CD44, PRKACB, PPP1CB, ITGB3, PIK3R2 | hsa-miR-3613-3p, hsa-miR-5100, hsa-miR-629-3p, hsa-miR-4668-5p, hsa-miR-3148,  hsa-miR-548ac, hsa-miR-664b-3p, hsa-miR-548x-3p, hsa-miR-1208, hsa-miR-4635,  hsa-miR-548a-3p, hsa-miR-7-1-3p, hsa-miR-8084, hsa-miR-6867-5p, hsa-miR-8063,  hsa-miR-6785-5p, hsa-miR-7-5p, hsa-miR-1468-3p, hsa-miR-4720-5p, hsa-miR-6744-5p,  hsa-miR-204-3p, hsa-miR-6825-5p, hsa-miR-885-3p, hsa-miR-330-5p, hsa-miR-6893-5p,  hsa-miR-98-5p, hsa-miR-152-3p, hsa-miR-27a-3p, hsa-let-7f-1-3p, hsa-miR-34a-5p,  hsa-miR-3128, hsa-miR-342-5p, hsa-miR-6837-5p, hsa-miR-4446-3p, hsa-miR-4663,  hsa-miR-4270, hsa-miR-5088-5p, hsa-miR-659-3p, hsa-miR-29c-3p, hsa-miR-635,  hsa-miR-6774-5p, hsa-miR-371b-5p, hsa-miR-101-5p, hsa-miR-550a-3-5p, hsa-miR-296-5p, hsa-miR-338-5p, hsa-miR-3194-3p, hsa-miR-16-2-3p, hsa-miR-195-3p, hsa-miR-3689f,  hsa-miR-4439, hsa-miR-3613-5p, hsa-miR-6813-5p, hsa-miR-3609, hsa-miR-3175,  hsa-miR-6830-5p, hsa-miR-4423-3p, hsa-miR-5189-3p, hsa-miR-4448, hsa-miR-6507-3p,  hsa-miR-574-5p, hsa-miR-196b-3p, hsa-miR-6886-5p |
| **Pathways in cancer** | BRAF, LAMB2, FGF12, FOS, GSK3B, PRKCA, DVL3, STAT3, PDGFRA, FZD5, E2F1, BDKRB1, TGFBR1, ERBB2, GNG13, NFKB1, MET, ADCY1, ADCY5, ITGB1, WNT7A, FIGF, PTGER4, ROCK1, SOS2, CDK4, LAMB1, CXCL8, FGF14, GNG11, SMAD2, CBL, E2F2, CXCR4, ADCY7, NRAS, FGFR3, CRKL, STK4, ADCY2, BID, AGTR1, LPAR3, FGF13, GNAS, APC, CRK, RUNX1, PIK3CB, PTCH1, MSH3, CUL2, TPR, WNT10B, TCF7L2, RXRG, WNT5A, CTBP1, TGFA, HHIP, COL4A5, ZBTB16, ARHGEF12, PIK3R5, CDK2, GNA13, BAX, GLI2, ROCK2, TCF7L1, BDKRB2, ARNT, ETS1, GNG12, BCR, RALA, FZD6, FGF10, CTBP2, RAF1, SMAD3, ITGA3, CHUK, LAMA1, RARB, BCL2, CDKN1B, WNT2B, TRAF4, BIRC5, RALBP1, PLD1, WNT4, CDKN2B, CXCL12, WNT3, BRCA2, IGF1R, EGFR, GNAI3, GNB1, FGF20, PTCH2, F2RL3, RHOA, TRAF5, APPL1, FZD8, KRAS, RET, CDK6, STAT5B, LPAR6, TGFB1, TCEB1, FZD3, FASLG, PML, TPM3, PAX8, VHL, EPAS1, PTGER3, ARHGEF1, MITF, LPAR4, FADD, IKBKB, FGF11, AKT2, PTK2, GNA11, CBLB, ITGAV, FH, ARHGEF11, PLCB1, CDH1, FZD4, MMP2, RUNX1T1, MAPK9, ARNT2, GNB2, DCC, AR, PLCG1, CASP3, PIK3CD, PIK3R3, CCND1, SMAD4, CTNNB1, LPAR1, MSH6, CTNNA1, CCNE2, AXIN2, RASGRP1, SKP2, COL4A2, HIF1A, E2F3, MAPK8, TRAF6, AKT1, PGF, CEBPA, F2R, MYC, NTRK1, EDNRB, PTGER2, GLI3, RASGRP4, DAPK1, PDGFB, PRKACG, PRKACA, GNG7, EGLN3, NFKBIA, FLT3LG, KIT, VEGFC, GNG2, PTGS2, COL4A3, ITGA2, PIK3R1, RB1, COL4A4, SOS1, FGF9, HSP90B1, HDAC2, PRKX, KITLG, NOS2, RAC1, MAPK3, FGF2, CDC42, RARA, CASP9, FGF18, BMP2, PLCG2, FZD1, FLT3, FGF5, EGLN2, PRKCB, MAX, FAS, LAMC1, IGF1, TGFB2, EP300, PPARG, CASP8, WNT10A, GNAQ, NKX3-1, BCL2L1, AKT3, PIAS2, CTNNA3, GNAI2, PIK3CA, RALGDS, RBX1, LEF1, COL4A6, ADCY8, MECOM, FN1, FGF8, CDKN1A, MAP2K1, BIRC3, RALB, SMO, SLC2A1, GNG4, EDNRA, RASGRP3, LAMA2, WNT2, CKS2, LAMC2, STAT1, RASSF1, CYCS, FGF23, HGF, WNT9B, MTOR, SUFU, FGFR2, ITGA6, SHH, RELA, RXRB, TCF7, CTNNA2, VEGFA, PTEN, FGFR1, FOXO1, FGF16, MAPK1, DAPK2, FGF1, CREBBP, FGF7, TRAF3, GNG5, GRB2, ADCY9, ADCY4, WNT7B, DVL2, GNB4, JUP, TRAF1, TGFBR2, TFG, PPARD, JAK1, MDM2, WNT9A, ABL1, EGF, PLCB4, PLCB2, STK36, BMP4, ITGA2B, GNB5, CCDC6, MAPK10, GNAI1, PRKACB, XIAP, EGLN1, COL4A1, PDGFA, LAMA4, ADCY6, PIK3R2 | hsa-miR-4663, hsa-miR-371b-5p, hsa-miR-7-5p, hsa-miR-629-3p, hsa-miR-29c-3p,  hsa-miR-3613-3p, hsa-miR-3148, hsa-miR-152-3p, hsa-miR-1468-3p, hsa-miR-330-5p,  hsa-miR-548ac, hsa-miR-27a-3p, hsa-miR-548x-3p, hsa-miR-6507-3p, hsa-miR-4635,  hsa-miR-7-1-3p, hsa-miR-34a-5p, hsa-miR-4668-5p, hsa-miR-548a-3p, hsa-miR-204-3p,  hsa-miR-4446-3p, hsa-miR-635, hsa-let-7f-1-3p, hsa-miR-6774-5p, hsa-miR-8063,  hsa-miR-6893-5p, hsa-miR-6830-5p, hsa-miR-574-5p, hsa-miR-338-5p, hsa-miR-342-5p,  hsa-miR-6785-5p, hsa-miR-664b-3p, hsa-miR-659-3p, hsa-miR-3689f, hsa-miR-98-5p,  hsa-miR-6813-5p, hsa-miR-4720-5p, hsa-miR-1208, hsa-miR-6825-5p, hsa-miR-550a-3-5p, hsa-miR-3613-5p, hsa-miR-3175, hsa-miR-6744-5p, hsa-miR-296-5p, hsa-miR-8084,  hsa-miR-6867-5p, hsa-miR-4462, hsa-miR-3128, hsa-miR-3194-3p, hsa-miR-4518,  hsa-miR-885-3p, hsa-miR-3609, hsa-miR-6886-5p, hsa-miR-6787-5p, hsa-miR-4448,  hsa-miR-16-2-3p, hsa-miR-195-3p, hsa-miR-6837-5p, hsa-miR-5189-3p, hsa-miR-4270,  hsa-miR-5088-5p, hsa-miR-5100, hsa-miR-4439, hsa-miR-335-5p, hsa-miR-3201,  hsa-miR-3605-3p, hsa-miR-4423-3p |
| **Arrhythmogenic right ventricular cardiomyopathy (ARVC)** | ACTB, CACNG8, DES, ACTN2, CDH2, CACNA2D3, DSC2, ITGB1, ITGB8, ITGA9, CACNA1F, ITGA8, CACNB4, TCF7L2, CACNG7, ACTG1, ITGB6, TCF7L1, SGCD, ITGA5, ITGA3, ITGA1, PKP2, ITGAV, ITGA11, DMD, CTNNB1, CTNNA1, RYR2, CACNB1, SLC8A1, DAG1, CACNG4, ITGA2, ITGA10, CACNA2D4, CACNA2D2, CACNG2, SGCA, DSG2, ACTN4, ITGA7, CTNNA3, LEF1, LMNA, GJA1, LAMA2, ITGA4, CACNG6, CACNA2D1, ATP2A2, CACNB2, ITGA6, CACNA1D, TCF7, CTNNA2, SGCB, JUP, ITGA2B, CACNB3, DSP, ITGB3 | hsa-miR-574-5p, hsa-miR-34a-5p, hsa-miR-4720-5p, hsa-miR-548ac, hsa-miR-548x-3p,  hsa-miR-6744-5p, hsa-miR-4518, hsa-miR-664b-3p, hsa-miR-3613-3p, hsa-miR-548a-3p,  hsa-miR-659-3p, hsa-let-7f-1-3p, hsa-miR-4663, hsa-miR-3128, hsa-miR-635, hsa-miR-7-1-3p, hsa-miR-6774-5p, hsa-miR-330-5p, hsa-miR-27a-3p, hsa-miR-3148, hsa-miR-1468-3p,  hsa-miR-7-5p, hsa-miR-4439, hsa-miR-8063, hsa-miR-8084, hsa-miR-4668-5p,  hsa-miR-6830-5p, hsa-miR-5100, hsa-miR-371b-5p, hsa-miR-6893-5p, hsa-miR-152-3p,  hsa-miR-29c-3p, hsa-miR-101-5p, hsa-miR-204-3p, hsa-miR-296-5p, hsa-miR-629-3p,  hsa-miR-6825-5p, hsa-miR-3175, hsa-miR-338-5p, hsa-miR-4635, hsa-miR-98-5p,  hsa-miR-885-3p, hsa-miR-6785-5p, hsa-miR-342-5p, hsa-miR-6837-5p, hsa-miR-4270,  hsa-miR-550a-3-5p, hsa-miR-16-2-3p, hsa-miR-195-3p, hsa-miR-5088-5p, hsa-miR-3201,  hsa-miR-1208, hsa-miR-5189-3p, hsa-miR-6867-5p, hsa-miR-3613-5p, hsa-miR-3194-3p |
| **Hippo signalling pathway** | FZD7, ACTB, GSK3B, DVL3, FZD5, TGFBR1, YWHAH, ID2, PARD6G, WNT7A, YAP1, SMAD2, YWHAE, BTRC, APC, PPP2R2C, PRKCI, WNT10B, PPP2CA, TCF7L2, NF2, WNT5A, DLG1, BMPR1B, YWHAG, PPP1CC, CCND2, BMP5, ACTG1, GLI2, TCF7L1, AREG, FZD6, SNAI2, SMAD3, WNT2B, BIRC5, WNT4, MOB1B, WNT3, YWHAB, CRB1, TEAD3, PPP2R2B, WWC1, AMOT, PPP2R2D, WWTR1, FZD8, TP53BP2, TGFB1, FZD3, BMP8B, LIMD1, YWHAQ, MPP5, LLGL2, AFP, CDH1, FZD4, CSNK1D, DLG4, CCND1, SMAD4, CTNNB1, CTNNA1, AXIN2, TP73, NKD1, PPP2R2A, MYC, RASSF6, PPP2R1A, LLGL1, DLG3, FRMD6, SAV1ID1, TEAD1, YWHAZ, GDF6, STK3, BMP2, FZD1, GDF5, TGFB2, CSNK1E, BBC3, WNT10A, BMPR1A, CTNNA3, WTIP, DLG2, LEF1, PARD3, WNT2, SMAD7, RASSF1, WNT9B, MOB1A, TCF7, CTNNA2, LATS1, SOX2, BMP7, FGF1, LATS2, FBXW1, PPP2R1B, SERPINE1, SMAD1, PARD6B, WNT7B, DVL2, INADL, TGFBR2, GDF7, WNT9A, BMP4, BMPR2, CCND3, AJUBA, CTGF, PPP1CB | hsa-miR-330-5p, hsa-miR-6774-5p, hsa-miR-4518, hsa-miR-885-3p, hsa-miR-6785-5p,  hsa-miR-6825-5p, hsa-miR-659-3p, hsa-miR-629-3p, hsa-miR-3613-3p, hsa-miR-548ac,  hsa-miR-548x-3p, hsa-miR-1208, hsa-miR-3194-3p, hsa-miR-8063, hsa-miR-3128,  hsa-miR-4720-5p, hsa-miR-34a-5p, hsa-miR-4668-5p, hsa-miR-3148, hsa-miR-152-3p,  hsa-miR-7-1-3p, hsa-miR-338-5p, hsa-miR-98-5p, hsa-miR-29c-3p, hsa-miR-548a-3p,  hsa-miR-27a-3p, hsa-miR-6744-5p, hsa-miR-6867-5p, hsa-miR-5189-3p, hsa-miR-4448,  hsa-miR-6837-5p, hsa-miR-4270, hsa-miR-550a-3-5p, hsa-miR-664b-3p, hsa-let-7f-1-3p,  hsa-miR-204-3p, hsa-miR-371b-5p, hsa-miR-1468-3p, hsa-miR-3689f, hsa-miR-8084,  hsa-miR-7-5p, hsa-miR-195-3p, hsa-miR-16-2-3p, hsa-miR-4446-3p, hsa-miR-6830-5p,  hsa-miR-3609, hsa-miR-574-5p, hsa-miR-3175, hsa-miR-5100, hsa-miR-4663, hsa-miR-4439,  hsa-miR-6893-5p, hsa-miR-6813-5p, hsa-miR-296-5p, hsa-miR-4423-3p, hsa-miR-4462,  hsa-miR-579-5p, hsa-miR-635, hsa-miR-4635, hsa-miR-3613-5p, hsa-miR-342-5p,  hsa-miR-5088-5p, hsa-miR-335-5p, hsa-miR-101-5p |
| **Renal cell carcinoma** | BRAF, MET, SOS2, RAPGEF1, NRAS, CRKL, CRK, PIK3CB, CUL2, PAK2, TGFA, PIK3R5, RAP1A, ARNT, ETS1, PAK7, RAF1, KRAS, PAK3, TGFB1, TCEB1, VHL, EPAS1, PAK1, AKT2, FH, ARNT2, PIK3CD, PIK3R3, HIF1A, AKT1, PDGFB, EGLN3, PIK3R1, SOS1, PTPN11, RAC1, MAPK3, PAK4, CDC42, PAK6, EGLN2, TGFB2, EP300, GAB1, AKT3, PAK6, PIK3CA, RBX1, MAP2K1, SLC2A1, HGF, VEGFA, MAPK1, CREBBP, GRB2, RAP1B, EGLN1, FLCN, PIK3R2 | hsa-miR-548a-3p, hsa-miR-34a-5p, hsa-miR-548ac, hsa-miR-664b-3p, hsa-miR-548x-3p,  hsa-miR-3194-3p, hsa-miR-7-5p, hsa-miR-7-1-3p, hsa-miR-6744-5p, hsa-miR-8063,  hsa-miR-3613-3p, hsa-miR-6785-5p, hsa-miR-6893-5p, hsa-miR-152-3p, hsa-miR-6837-5p, hsa-miR-3609, hsa-miR-6825-5p, hsa-miR-6867-5p, hsa-miR-338-5p, hsa-miR-16-2-3p,  hsa-miR-195-3p, hsa-miR-8084, hsa-miR-335-5p, hsa-miR-5100, hsa-miR-5189-3p,  hsa-miR-101-5p, hsa-miR-371b-5p, hsa-miR-27a-3p, hsa-miR-4462, hsa-miR-4668-5p,  hsa-miR-3148, hsa-let-7f-1-3p, hsa-miR-3689f, hsa-miR-1208, hsa-miR-330-5p,  hsa-miR-29c-3p, hsa-miR-3128, hsa-miR-6830-5p, hsa-miR-4439, hsa-miR-4448,  hsa-miR-1468-3p, hsa-miR-659-3p, hsa-miR-342-5p, hsa-miR-4635, hsa-miR-574-5p,  hsa-miR-4270, hsa-miR-3613-5p, hsa-miR-98-5p, hsa-miR-6886-5p, hsa-miR-629-3p,  hsa-miR-3175, hsa-miR-885-3p, hsa-miR-4518, hsa-miR-6507-3p, hsa-miR-204-3p,  hsa-miR-6813-5p |
| **ECM-receptor interaction** | LAMB2, GP5, ITGB1, ITGB8, LAMB1, ITGA9, SDC1, CD36, SV2B, THBS1, ITGA8, THBS2, COL4A5, COL24A1, COL27A1, ITGB6, AGRN, ITGA5, ITGA3, LAMA1, COL6A6, ITGA1, COL3A1, SV2A, HSPG2, COL6A1, ITGAV, ITGA11, COL2A1, GP1BA, COL4A2, RELN, COL5A1, COL1A1, COL4A3, DAG1, ITGA2, COL4A4, ITGA10, COL11A2, COL1A2, LAMC1, ITGA7, COL11A1, THBS3, COL6A3, COL4A6, SV2C, FN1, SDC4, TNR, LAMA2, ITGA4, LAMC2, ITGA6, TNN, COL5A3, COL5A2, SPP1, ITGA2B, SEPT5, CD44, CD47, COL4A1, LAMA4, ITGB3 | hsa-miR-4668-5p, hsa-miR-3148, hsa-miR-27a-3p, hsa-miR-548x-3p, hsa-miR-574-5p,  hsa-miR-6785-5p, hsa-miR-3175, hsa-miR-6825-5p, hsa-miR-296-5p, hsa-miR-152-3p,  hsa-miR-3613-3p, hsa-miR-98-5p, hsa-miR-548a-3p, hsa-miR-7-1-3p, hsa-miR-6893-5p,  hsa-miR-29c-3p, hsa-miR-664b-3p, hsa-miR-195-3p, hsa-miR-338-5p, hsa-miR-3201,  hsa-miR-548ac, hsa-let-7f-1-3p, hsa-miR-8063, hsa-miR-6830-5p, hsa-miR-629-3p,  hsa-miR-635, hsa-miR-6774-5p, hsa-miR-4439, hsa-miR-3128, hsa-miR-330-5p,  hsa-miR-6867-5p, hsa-miR-3194-3p, hsa-miR-4663, hsa-miR-4270, hsa-miR-885-3p,  hsa-miR-1468-3p, hsa-miR-34a-5p, hsa-miR-3613-5p, hsa-miR-1208, hsa-miR-101-5p,  hsa-miR-4448, hsa-miR-204-3p, hsa-miR-4720-5p, hsa-miR-4446-3p, hsa-miR-550a-3-5p, hsa-miR-7-5p, hsa-miR-3689f, hsa-miR-8084, hsa-miR-371b-5p, hsa-miR-6837-5p,  hsa-miR-5100, hsa-miR-6744-5p, hsa-miR-16-2-3p, hsa-miR-5088-5p, hsa-miR-4635 |
| **Axon guidance** | EFNB2, SEMA3G, SEMA6A, PLXNA2, GSK3B, ABLIM3, EPHB2, NTNG2, MET, ITGB1, ROCK1, SEMA4A, L1CAM, CXCR4, NRAS, CFL1, PAK2, ARHGEF12, SEMA5A, EPHA5, ROCK2, ABLIM2, PAK7, ROBO2, NTN1, PPP3R1, SEMA3C, SEMA4F, FES, SRGAP1, NTNG1, CXCL12, SEMA5B, EFNA3, LRRC4C, GNAI3, EFNA2, RHOA, KRAS, PAK3, FYN, SEMA6B, EFNA5, EPHA7, PAK1, SEMA3F, EFNA4, NFATC4, EPHB3, SEMA4G, NCK1, PPP3CA, PTK2, RASA1, PLXNA3, EFNB3, SLIT2, DCC, EPHB4, PPP3CB, SRGAP3, EPHA3, NCK2, DPYSL5, DPYSL2, SLIT1, NFATC2, LIMK2, UNC5C, CFL2, SEMA3D, NRP1, SEMA4C, RAC1, MAPK3, PAK4, CDC42, PAK6, PLXNB1, SEMA4B, SEMA3A, PAK6, EFNB1, EPHB6, GNAI2, PLXNC1, NTN4, SEMA7A, LIMK1, ABLIM1, SEMA4D, ROBO1, SEMA6D, SLIT3, EPHA4, NFATC3, UNC5D, MAPK1, LRRC4, ABL1, EPHB1, GNAI1, PPP3R2, EFNA1 | hsa-miR-6893-5p, hsa-miR-6785-5p, hsa-miR-7-5p, hsa-miR-629-3p, hsa-miR-548x-3p,  hsa-miR-3201, hsa-miR-664b-3p, hsa-miR-1208, hsa-miR-7-1-3p, hsa-miR-338-5p,  hsa-miR-8063, hsa-miR-3128, hsa-miR-3613-3p, hsa-miR-6830-5p, hsa-miR-548a-3p,  hsa-miR-34a-5p, hsa-miR-548ac, hsa-let-7f-1-3p, hsa-miR-204-3p, hsa-miR-4663,  hsa-miR-98-5p, hsa-miR-27a-3p, hsa-miR-550a-3-5p, hsa-miR-6825-5p, hsa-miR-4668-5p, hsa-miR-3148, hsa-miR-371b-5p, hsa-miR-6837-5p, hsa-miR-330-5p, hsa-miR-4270,  hsa-miR-4635, hsa-miR-8084, hsa-miR-296-5p, hsa-miR-574-5p, hsa-miR-16-2-3p,  hsa-miR-195-3p, hsa-miR-6813-5p, hsa-miR-635, hsa-miR-1468-3p, hsa-miR-659-3p,  hsa-miR-3609, hsa-miR-3175, hsa-miR-4446-3p, hsa-miR-3613-5p, hsa-miR-152-3p,  hsa-miR-342-5p, hsa-miR-6867-5p, hsa-miR-101-5p, hsa-miR-5189-3p, hsa-miR-6744-5p, hsa-miR-29c-3p, hsa-miR-6774-5p, hsa-miR-4720-5p, hsa-miR-1260b, hsa-miR-3194-3p,  hsa-miR-5088-5p, hsa-miR-3689f, hsa-miR-6507-3p, hsa-miR-4439, hsa-miR-885-3p |
| **ErbB signalling pathway** | CAMK2D, BRAF, GSK3B, HBEGF, PRKCA, ERBB2, SOS2, CBL, NRAS, CRKL, NRG4, CRK, SHC1, PIK3CB, PAK2, MAP2K7, TGFA, PIK3R5, PAK7, AREG, RAF1, CDKN1B, SHC2, EGFR, ERBB3, KRAS, PAK3, STAT5B, SHC3, RPS6KB2, PAK1, CAMK2A, NCK1, AKT2, PTK2, CBLB, MAPK9, PLCG1, PIK3CD, PIK3R3, NCK2, MAPK8, AKT1, MYC, NRG3, PIK3R1, SOS1, MAPK3, PAK4, PAK6, PLCG2, BTC, NRG1, PRKCB, GAB1, SHC4, AKT3, CAMK2B, PAK6, PIK3CA, CDKN1A, MAP2K1, MTOR, MAP2K4, MAPK1, ABL2, GRB2, ELK1, ABL1, EGF, ERBB4, MAPK10, RPS6KB1, EREG, PIK3R2 | hsa-miR-3613-3p, hsa-miR-330-5p, hsa-miR-6785-5p, hsa-miR-3175, hsa-miR-548ac,  hsa-miR-6825-5p, hsa-miR-548x-3p, hsa-miR-338-5p, hsa-miR-7-1-3p, hsa-miR-6893-5p,  hsa-miR-204-3p, hsa-miR-6837-5p, hsa-miR-4446-3p, hsa-miR-34a-5p, hsa-miR-4270,  hsa-miR-3148, hsa-miR-342-5p, hsa-miR-548a-3p, hsa-miR-664b-3p, hsa-miR-27a-3p,  hsa-miR-371b-5p, hsa-miR-8063, hsa-miR-1468-3p, hsa-let-7f-1-3p, hsa-miR-7-5p,  hsa-miR-98-5p, hsa-miR-1208, hsa-miR-4635, hsa-miR-6813-5p, hsa-miR-3194-3p,  hsa-miR-3613-5p, hsa-miR-4668-5p, hsa-miR-152-3p, hsa-miR-3609, hsa-miR-3128,  hsa-miR-4720-5p, hsa-miR-6830-5p, hsa-miR-4439, hsa-miR-16-2-3p, hsa-miR-195-3p,  hsa-miR-574-5p, hsa-miR-4663, hsa-miR-29c-3p, hsa-miR-659-3p, hsa-miR-629-3p,  hsa-miR-8084, hsa-miR-6867-5p, hsa-miR-6886-5p, hsa-miR-3689f, hsa-miR-6744-5p,  hsa-miR-5100, hsa-miR-4518, hsa-miR-885-3p, hsa-miR-550a-3-5p, hsa-miR-6507-3p |
| **TGF-beta signalling pathway** | FST, TGFBR1, ID2, ROCK1, SMAD2, SMAD6, NODAL, INHBB, SMAD9, THBS1, PPP2CA, ACVR1B, SMURF2, BMPR1B, BMP5, PITX2, SMAD3, CHRD, CUL1, INHBA, CDKN2B, LEFTY1, ID4, RHOA, TGFB1, BMP8B, ACVR1, SKP1, RPS6KB2, ACVR2B, ZFYVE16, DCN, SMAD4, E2F5, MYC, PPP2R1A, SMURF1, RBL1, SMAD5, ID1, ACVR2A, GDF6, MAPK3, BMP2, TFDP1, SP1, ACVR1C, GDF5, TGFB2, EP300, BMPR1A, RBX1, IFNG, LTBP1, ID3, SMAD7, TNF, NOG, BMP7, MAPK1, CREBBP, PPP2R1B, SMAD1, TGFBR2, GDF7, BMP4, BMPR2, RPS6KB1 | hsa-miR-3613-3p, hsa-miR-3148, hsa-miR-548ac, hsa-let-7f-1-3p, hsa-miR-6867-5p,  hsa-miR-548x-3p, hsa-miR-7-1-3p, hsa-miR-98-5p, hsa-miR-1208, hsa-miR-8063,  hsa-miR-885-3p, hsa-miR-330-5p, hsa-miR-34a-5p, hsa-miR-4668-5p, hsa-miR-6830-5p,  hsa-miR-574-5p, hsa-miR-4635, hsa-miR-338-5p, hsa-miR-27a-3p, hsa-miR-4462,  hsa-miR-548a-3p, hsa-miR-664b-3p, hsa-miR-5100, hsa-miR-1468-3p, hsa-miR-3689f,  hsa-miR-6744-5p, hsa-miR-152-3p, hsa-miR-6774-5p, hsa-miR-6893-5p, hsa-miR-204-3p,  hsa-miR-6813-5p, hsa-miR-371b-5p, hsa-miR-8084, hsa-miR-6825-5p, hsa-miR-16-2-3p,  hsa-miR-195-3p, hsa-miR-5088-5p, hsa-miR-3175, hsa-miR-629-3p, hsa-miR-7-5p,  hsa-miR-4448, hsa-miR-3194-3p, hsa-miR-3128, hsa-miR-3613-5p, hsa-miR-4423-3p,  hsa-miR-29c-3p, hsa-miR-6785-5p, hsa-miR-659-3p, hsa-miR-550a-3-5p, hsa-miR-4446-3p, hsa-miR-635, hsa-miR-4270, hsa-miR-6787-5p, hsa-miR-4518, hsa-miR-101-5p |
| **Thyroid hormone signalling pathway** | ESR1, ACTB, GSK3B, PRKCA, ATP1B2, MED13L, SLCO1C1, PLCD1, NRAS, HDAC3, RCAN1, MED14, PIK3CB, MED12, PLCD3, ATP1A2, RXRG, DIO2, PIK3R5, ACTG1, RAF1, MED13, SLC16A10, WNT4, ATP1B1, THRA, PLCZ1, NOTCH2, MED17, RCAN2, KRAS, RHEB, MED12L, NCOA3, GATA4, MED1, MED4, AKT2, MED16, ITGAV, PLCB1, SLC9A1, TBC1D4, NOTCH1, PLCG1, TSC2, PIK3CD, PIK3R3, CCND1, CTNNB1, ATP1B3, HIF1A, PLN, AKT1, MYC, MED30, PRKACG, PRKACA, NCOR1, NCOA2, PIK3R1, HDAC2, PRKX, KAT2A, ATP1B4, MAPK3, CASP9, PLCG2, THRB, PRKCB, KAT2B, EP300, NOTCH3, AKT3, SLC16A2, PLCE1, PIK3CA, MAP2K1, SLC2A1, PDPK1, PFKFB2, ATP2A2, STAT1, MTOR, ATP1A1 RXRB, FOXO1, MAPK1, CREBBP, MDM2, PLCB4, PLCB2, BMP4, NCOA1, SIN3A, PRKACB, ITGB3, PIK3R2 | hsa-miR-3613-3p, hsa-miR-29c-3p, hsa-miR-548a-3p, hsa-miR-548ac, hsa-miR-664b-3p,  hsa-miR-548x-3p, hsa-miR-8084, hsa-let-7f-1-3p, hsa-miR-3194-3p, hsa-miR-8063,  hsa-miR-3148, hsa-miR-629-3p, hsa-miR-1208, hsa-miR-4448, hsa-miR-4270, hsa-miR-4635, hsa-miR-574-5p, hsa-miR-7-1-3p, hsa-miR-659-3p, hsa-miR-6825-5p, hsa-miR-1468-3p,  hsa-miR-4720-5p, hsa-miR-6744-5p, hsa-miR-338-5p, hsa-miR-330-5p, hsa-miR-7-5p,  hsa-miR-4423-3p, hsa-miR-27a-3p, hsa-miR-6893-5p, hsa-miR-204-3p, hsa-miR-342-5p,  hsa-miR-34a-5p, hsa-miR-6837-5p, hsa-miR-4518, hsa-miR-635, hsa-miR-6774-5p,  hsa-miR-6867-5p, hsa-miR-6785-5p, hsa-miR-371b-5p, hsa-miR-4668-5p, hsa-miR-3609,  hsa-miR-1260b, hsa-miR-5100, hsa-miR-6507-3p, hsa-miR-6830-5p, hsa-miR-98-5p,  hsa-miR-152-3p, hsa-miR-3689f, hsa-miR-3175, hsa-miR-550a-3-5p, hsa-miR-4439,  hsa-miR-5189-3p, hsa-miR-6886-5p, hsa-miR-16-2-3p, hsa-miR-195-3p, hsa-miR-6813-5p, hsa-miR-5088-5p, hsa-miR-101-5p, hsa-miR-885-3p, hsa-miR-4663, hsa-miR-4446-3p |
| **Signalling pathways regulating pluripotency of stem cells** | BMI1, FZD7, PCGF6, JARID2, TCF3, GSK3B, DVL3, STAT3, FZD5, OTX1, ID2, WNT7A, KAT6A, PAX6, SMAD2, NRAS, FGFR3, NODAL, INHBB, SMAD9, APC, HOXB1, PIK3CB, WNT10B, REST, FGFR4, ACVR1B, WNT5A, BMPR1B, PIK3R5, MAPK14, TBX3, HAND1, FZD6, RAF1, SMAD3, SMARCAD1, WNT2B, INHBA, WNT4, WNT3, IGF1R, ZFHX3, ID4, FZD8, HESX1, KRAS, FZD3, POU5F1B, ACVR1, ACVR2B, AKT2, PCGF5, SETDB1, LIF, FZD4, RIF1, DLX5, PIK3CD, PIK3R3, JAK2, SMAD4, CTNNB1, AXIN2, AKT1, MYC, LIFR, SKIL, ESRRB, ZIC3, SMAD5, ONECUT1, ID1, PIK3R1, JAK3, ACVR2A, MAPK3, FGF2, BMP2, LHX5, FZD1, ACVR1C, IGF1, WNT10A, AKT3, BMPR1A, NANOG, PIK3CA, NEUROG1, IL6ST, MAP2K1, ISL1, WNT2, ID3, WNT9B, DUSP9, FGFR2, FGFR1, SOX2, PCGF1, MAPK1, PCGF3, GRB2, SMAD1, WNT7B, DVL2, KLF4, JAK1, WNT9A BMP4, MEIS1, BMPR2, COMMD3-BMI1, PIK3R2 | hsa-miR-29c-3p, hsa-miR-6785-5p, hsa-miR-548x-3p, hsa-miR-3613-3p, hsa-miR-98-5p,  hsa-miR-3148, hsa-miR-152-3p, hsa-miR-1468-3p, hsa-miR-548ac, hsa-miR-8063,  hsa-let-7f-1-3p, hsa-miR-4462, hsa-miR-204-3p, hsa-miR-664b-3p, hsa-miR-7-1-3p,  hsa-miR-338-5p, hsa-miR-27a-3p, hsa-miR-4668-5p, hsa-miR-548a-3p, hsa-miR-6825-5p, hsa-miR-6867-5p, hsa-miR-574-5p, hsa-miR-16-2-3p, hsa-miR-195-3p, hsa-miR-885-3p,  hsa-miR-3175, hsa-miR-8084, hsa-miR-101-5p, hsa-miR-629-3p, hsa-miR-4439,  hsa-miR-342-5p, hsa-miR-371b-5p, hsa-miR-330-5p, hsa-miR-4635, hsa-miR-34a-5p,  hsa-miR-5100, hsa-miR-659-3p, hsa-miR-4720-5p, hsa-miR-6744-5p, hsa-miR-3194-3p,  hsa-miR-1208, hsa-miR-7-5p, hsa-miR-4270, hsa-miR-3613-5p, hsa-miR-5189-3p,  hsa-miR-6830-5p, hsa-miR-6893-5p, hsa-miR-3689f, hsa-miR-296-5p, hsa-miR-4448,  hsa-miR-579-5p, hsa-miR-3128, hsa-miR-3201, hsa-miR-6886-5p, hsa-miR-3609,  hsa-miR-6507-3p, hsa-miR-1260b, hsa-miR-6837-5p, hsa-miR-6813-5p, hsa-miR-4423-3p, hsa-miR-6774-5p |
| **Adherens junction** | ACTB, ACTN2, CSNK2A2, TGFBR1, ERBB2, MET, WASF1, WASL, CTNND1, SMAD2, PVRL2, TCF7L2, ACTG1, TCF7L1, LMO7, IQGAP1, SNAI2, SMAD3, PTPRM, PTPN1, IGF1R, EGFR, VCL, PTPRF, SNAI1, RHOA, WASF3, TJP1, SORBS1, FYN, MLLT4, NLK, CDH1, PTPN6, SMAD4, CTNNB1, PVRL4, CTNNA1, WASF2, CSNK2B, FER, CSNK2A1, FARP2, PTPRJ, RAC1, INSR, MAPK3, CDC42, SSX2IP, ACTN4, EP300, CTNNA3, LEF1, PARD3, PVRL3, YES1, PTPRB, TCF7, CTNNA2, FGFR1, MAPK1, MAP3K7, CREBBP, TGFBR2, PVRL1 | hsa-miR-548a-3p, hsa-let-7f-1-3p, hsa-miR-6867-5p, hsa-miR-548x-3p, hsa-miR-3613-3p, hsa-miR-4668-5p, hsa-miR-3148, hsa-miR-6830-5p, hsa-miR-548ac, hsa-miR-574-5p,  hsa-miR-4635, hsa-miR-338-5p, hsa-miR-8084, hsa-miR-664b-3p, hsa-miR-27a-3p,  hsa-miR-4439, hsa-miR-7-5p, hsa-miR-1468-3p, hsa-miR-4446-3p, hsa-miR-7-1-3p,  hsa-miR-635, hsa-miR-659-3p, hsa-miR-6774-5p, hsa-miR-8063, hsa-miR-34a-5p,  hsa-miR-6893-5p, hsa-miR-885-3p, hsa-miR-6785-5p, hsa-miR-6825-5p, hsa-miR-3609,  hsa-miR-5189-3p, hsa-miR-16-2-3p, hsa-miR-3128, hsa-miR-98-5p, hsa-miR-6837-5p,  hsa-miR-4270, hsa-miR-204-3p, hsa-miR-330-5p, hsa-miR-629-3p, hsa-miR-29c-3p,  hsa-miR-5088-5p, hsa-miR-550a-3-5p, hsa-miR-4448, hsa-miR-3194-3p, hsa-miR-6886-5p, hsa-miR-371b-5p, hsa-miR-6813-5p, hsa-miR-1208, hsa-miR-1260b, hsa-miR-152-3p,  hsa-miR-3613-5p, hsa-miR-3175, hsa-miR-4445-3p, hsa-miR-3689f, hsa-miR-6744-5p,  hsa-miR-4663, hsa-miR-5100, hsa-miR-4720-5p, hsa-miR-4518, hsa-miR-4462 |
| **Focal adhesion** | PARVG, BRAF, LAMB2, ACTB, TLN2, GSK3B, PRKCA, PDGFRA, ACTN2, CAPN2, MYLK4, ERBB2, MET, ITGB1, FIGF, ROCK1, SOS2, ITGB8, LAMB1, FLNC, RAPGEF1, ITGA9, CRKL, CRK, SHC1, THBS1, ITGA8, PIK3CB, MYL12B, PAK2, THBS2, CAV1, COL4A5, COL24A1, PIK3R5, PPP1CC, RAP1A, CCND2, COL27A1, ACTG1, PXN, ROCK2, ITGB6, PAK7, ITGA5, RAF1, ITGA3, LAMA1, BCL2, SHC2, IGF1R, EGFR, MYLK2, ZYX, VCL, RHOA, PPP1R12B, COL6A6, CAV2, ITGA1, PAK3, COL3A1, FYN, SHC3, PAK1, VAV2, COL6A1, AKT2, ARHGAP35, PTK2, ITGAV, ITGA11, COL2A1, PPP1R12A, MAPK9, PIK3CD, PIK3R3, CCND1, CTNNB1, DOCK1l, COL4A2, MAPK8, AKT1, PGF, PARVB, PARVA, DIAPH1, RELN, COL5A1, FLNB, PDGFB, COL1A1, FLT1, VEGFC, FLNA, COL4A3, ITGA2, PIK3R1, COL4A4, SOS1, ITGA10, COL11A2, RAC1, MAPK3, PAK4, CDC42, PAK6, COL1A2, ACTN4, PRKCB, RASGRF1, LAMC1, IGF1, ITGA7, SHC4, AKT3, PDGFC, MYLK3, COL11A1, PAK6, THBS3, FLT4, COL6A3, PDGFD, PIK3CA, VASP, COL4A6, FN1, MAP2K1, BIRC3, TNR, PDPK1, LAMA2, ITGA4, LAMC2, HGF, ITGA6, TNN, VAV3, COL5A3, VEGFA, PTEN, MAPK1, TLN1, KDR, GRB2, RAP1B, COL5A2, SPP1, ARHGAP5, ELK1, EGF, MYLK, ITGA2B, CCND3, ILK, MAPK10, XIAP, COL4A1, MYL5, PPP1CB, PDGFA, LAMA4, ITGB3, PIK3R2 | hsa-miR-629-3p, hsa-miR-330-5p, hsa-miR-27a-3p, hsa-miR-6825-5p, hsa-miR-296-5p,  hsa-miR-3613-3p, hsa-miR-8063, hsa-miR-4270, hsa-miR-29c-3p, hsa-miR-664b-3p,  hsa-miR-16-2-3p, hsa-miR-195-3p, hsa-miR-4668-5p, hsa-miR-3148, hsa-miR-204-3p,  hsa-let-7f-1-3p, hsa-miR-7-1-3p, hsa-miR-6893-5p, hsa-miR-152-3p, hsa-miR-548x-3p,  hsa-miR-371b-5p, hsa-miR-4439, hsa-miR-4663, hsa-miR-98-5p, hsa-miR-548a-3p,  hsa-miR-548ac, hsa-miR-574-5p, hsa-miR-338-5p, hsa-miR-1468-3p, hsa-miR-6813-5p,  hsa-miR-6837-5p, hsa-miR-635, hsa-miR-6774-5p, hsa-miR-1208, hsa-miR-34a-5p,  hsa-miR-3194-3p, hsa-miR-7-5p, hsa-miR-6744-5p, hsa-miR-6867-5p, hsa-miR-4635,  hsa-miR-4448, hsa-miR-6785-5p, hsa-miR-4720-5p, hsa-miR-8084, hsa-miR-335-5p,  hsa-miR-3128, hsa-miR-659-3p, hsa-miR-3613-5p, hsa-miR-4446-3p, hsa-miR-342-5p,  hsa-miR-3609, hsa-miR-3175, hsa-miR-885-3p, hsa-miR-3689f, hsa-miR-6507-3p,  hsa-miR-5100, hsa-miR-5189-3p, hsa-miR-101-5p, hsa-miR-550a-3-5p, hsa-miR-6830-5p, hsa-miR-4423-3p, hsa-miR-3201, hsa-miR-5088-5p, hsa-miR-6886-5p |
| **Ras signalling pathway** | RASAL1, FGF12, PRKCA, KSR2, PDGFRA, GNG13, NFKB1, MET, FIGF, SOS2, KSR1, FGF14, GNG11, NRAS, FGFR3, STK4, RASA2, CALM3, CALM1, FGF13, SHC1, PIK3CB, PAK2, FGFR4, RASAL3, PIK3R5, RAP1A, ETS2, RASGRF2, ETS1, PAK7, GNG12, ANGPT2, RALA, FGF10, PLA2G4F, RAF1, CHUK, FOXO4, RALBP1, PLD1, SHC2, TIAM1, EFNA3, IGF1R, EGFR, MRAS, EFNA2, GNB1, FGF20, RHOA, KRAS, PAK3, SHC3, CALM2, RRAS2, FASLG, EFNA5, PAK1, EFNA4, IKBKB, FGF11, RAB5A, MLLT, AKT2, PLA2G3, RASA1, MAPK9, GNB2, PLCG1, PIK3CD, PIK3R3, PLA2G12A, RASGRP1, MAPK, AKT1, PGF, RGL2, RASGRP4, REL, PDGFB, PRKACG, BRAP, PRKACA, GNG7, FLT1, KIT, VEGFC, PLA2G2F, GNG2, PLA2G2E, PIK3R1, SOS1, FGF9, PTPN11, PRKX, RASA4, TBK1, KITLG, RAC1, INSR, MAPK3, RASA3, PAK4, FGF2, CDC42, PAK6, FGF18, PLCG2, PLA2G4D, NF1, NGF, FGF5, PRKCB, RASGRF1, IGF1, GAB1, SHC4, BCL2L1, AKT3, PDGFC, PAK6, ANGPT1, FLT4, PLCE1, PDGFD, PIK3CA, RALGDS, SYNGAP1, GAB2, FGF8, MAP2K1, RALB, ARF6, GNG4, RASAL2, RASGRP3, RGL1, RASSF1, FGF23, HGF, RAB5C, FGFR2, GRIN1, PLA2G4E, RELA, VEGFA, HTR7, FGFR1, FGF16, MAPK1, FGF1, ABL2, FGF7, KDR, CSF1, GNG5, GRIN2A, GRB2, RAP1B, GNB4, TEK, RAB5B, ELK1, NGFR, RAPGEF5, ABL1, EGF, GNB5, PLA2G4C, RRAS, MAPK10, PRKACB, PDGFA, EFNA1, GRIN2B, PIK3R2 | hsa-miR-29c-3p, hsa-miR-6867-5p, hsa-miR-338-5p, hsa-miR-6893-5p, hsa-miR-3148,  hsa-miR-548ac, hsa-miR-548x-3p, hsa-miR-371b-5p, hsa-miR-8063, hsa-miR-3613-3p,  hsa-let-7f-1-3p, hsa-miR-3613-5p, hsa-miR-6785-5p, hsa-miR-98-5p, hsa-miR-152-3p,  hsa-miR-6837-5p, hsa-miR-330-5p, hsa-miR-664b-3p, hsa-miR-659-3p, hsa-miR-4270,  hsa-miR-8084, hsa-miR-4668-5p, hsa-miR-1468-3p, hsa-miR-27a-3p, hsa-miR-3689f,  hsa-miR-16-2-3p, hsa-miR-195-3p, hsa-miR-7-1-3p, hsa-miR-3128, hsa-miR-548a-3p,  hsa-miR-6825-5p, hsa-miR-635, hsa-miR-3194-3p, hsa-miR-6774-5p, hsa-miR-550a-3-5p, hsa-miR-5100, hsa-miR-4720-5p, hsa-miR-1208, hsa-miR-204-3p, hsa-miR-6813-5p,  hsa-miR-6507-3p, hsa-miR-4635, hsa-miR-3175, hsa-miR-6787-5p, hsa-miR-4423-3p,  hsa-miR-7-5p, hsa-miR-342-5p, hsa-miR-885-3p, hsa-miR-5088-5p, hsa-miR-4439,  hsa-miR-4663, hsa-miR-335-5p, hsa-miR-5189-3p, hsa-miR-101-5p, hsa-miR-574-5p,  hsa-miR-34a-5p, hsa-miR-629-3p, hsa-miR-6830-5p, hsa-miR-4446-3p, hsa-miR-296-5p,  hsa-miR-4518, hsa-miR-3609, hsa-miR-1260b, hsa-miR-3201, hsa-miR-6746-5p,  hsa-miR-6886-5p, hsa-miR-4448, hsa-miR-6744-5p |
| **Rap1 signalling pathway** | PFN1, BRAF, ACTB, MAGI2, RAPGEF3, FGF12, TLN2, PRKCA, PDGFRA, MET, SIPA1L3, ADCY1, ADCY5, ITGB1, PARD6G, FIGF, RAPGEF1, CTNND1, FGF14, ADCY7, NRAS, FGFR3, ADORA2B, CRKL, ADCY2, CALM3, CALM1, LPAR3, FGF13, GNAS, CRK, THBS1, PIK3CB, PRKCI, FGFR4, PIK3R5, RAP1A, MAPK14, ACTG1, ANGPT2, RALA, FGF10, RAF1, MAGI3, MAP2K3, TIAM1, EFNA3, IGF1R, EGFR, GNAI3, MRAS, EFNA2, FGF20, F2RL3, RHOA, KRAS, CALM2, EFNA5, ADORA2A, MAP2K6, LPAR4, EFNA4, SIPA1L1, RAPGEF4, VAV2, FGF11, MLLT4, AKT2, KRIT1, PLCB1, CDH1, RAP1GAP, DRD2, PLCG1, PIK3CD, PIK3R3, PFN2, CTNNB1, LPAR1, RAPGEF2, AKT1, PGF, F2R, RAPGEF6, ITGAM, PDGFB, FLT1, KIT, VEGFC, ID1, PIK3R1, DOCK4, FGF9, FARP2, CNR1, KITLG, RAC1, INSR, MAPK3, FGF2, CDC42, FGF18, P2RY1, NGF, FGF5, PRKCB, FYB, IGF1, GNAQ, AKT3, PDGFC, MAGI1, ANGPT1, FLT4, PLCE1, PDGFD, GNAI2, PIK3CA, VASP, RALGDS, GNAO1, ADCY8, FGF8, MAP2K1, RALB, PRKD3, RASGRP3, SIPA1L2, PARD3, APBB1IP, PRKD1, FGF23, HGF, PRKD2, FGFR2, GRIN1, LCP2, VEGFA, FGFR1, FGF16, MAPK1, TLN1, FGF1, FGF7, KDR, CSF1, GRIN2A, ADCY9, ADCY4, PARD6B, RAP1B, TEK, NGFR, RAPGEF5, ITGAL, EGF, PLCB4, PLCB2, ITGA2B, RRAS, GNAI1, PDGFA, ADCY6, EFNA1, ITGB3, GRIN2B, PIK3R2 | hsa-miR-3613-3p, hsa-miR-4668-5p, hsa-miR-6830-5p, hsa-miR-548a-3p, hsa-miR-548ac, hsa-miR-664b-3p, hsa-miR-27a-3p, hsa-miR-4439, hsa-miR-548x-3p, hsa-miR-1208,  hsa-miR-16-2-3p, hsa-miR-195-3p, hsa-miR-7-1-3p, hsa-miR-338-5p, hsa-miR-1468-3p,  hsa-miR-34a-5p, hsa-miR-7-5p, hsa-miR-3689f, hsa-miR-8063, hsa-miR-3128, hsa-miR-3148, hsa-miR-659-3p, hsa-miR-330-5p, hsa-miR-4635, hsa-miR-342-5p, hsa-miR-371b-5p,  hsa-miR-8084, hsa-miR-6867-5p, hsa-miR-3609, hsa-miR-6785-5p, hsa-miR-3194-3p,  hsa-miR-98-5p, hsa-miR-335-5p, hsa-miR-4462, hsa-let-7f-1-3p, hsa-miR-550a-3-5p,  hsa-miR-4663, hsa-miR-4446-3p, hsa-miR-6825-5p, hsa-miR-296-5p, hsa-miR-5088-5p,  hsa-miR-885-3p, hsa-miR-29c-3p, hsa-miR-152-3p, hsa-miR-6837-5p, hsa-miR-4270,  hsa-miR-635, hsa-miR-6774-5p, hsa-miR-3613-5p, hsa-miR-574-5p, hsa-miR-6744-5p,  hsa-miR-5100, hsa-miR-5189-3p, hsa-miR-6893-5p, hsa-miR-629-3p, hsa-miR-204-3p,  hsa-miR-3175, hsa-miR-6813-5p, hsa-miR-101-5p, hsa-miR-6886-5p, hsa-miR-3201,  hsa-miR-4448 |
| **Lysine degradation** | SETMAR, CAMKMT, EHMT2, WHSC1L1, ALDH7A1, SETD7, ALDH3A2, AASS, SETD1B, SETDB2, PLOD2, SETD2, NSD1, ASH1L, SETDB1, TMLHE, SETD8, AADAT, PHYKPL, COLGALT2, HYKK, KMT2D, PIPOX, SUV420H1, DOT1L, WHSC, ALDH1B1, SUV420H2, KMT2A, EHHADH, BBOX1, SUV39H2, DLST, KMT2E, KMT2C, SETD1A, COLGALT1, EHMT1, SUV39H1 | hsa-miR-4668-5p, hsa-miR-152-3p, hsa-miR-29c-3p, hsa-miR-34a-5p, hsa-miR-27a-3p,  hsa-let-7f-1-3p, hsa-miR-5088-5p, hsa-miR-574-5p, hsa-miR-6893-5p, hsa-miR-3613-3p,  hsa-miR-548ac, hsa-miR-548x-3p, hsa-miR-7-5p, hsa-miR-6830-5p, hsa-miR-98-5p,  hsa-miR-4448, hsa-miR-548a-3p, hsa-miR-8063, hsa-miR-3148, hsa-miR-1468-3p,  hsa-miR-664b-3p, hsa-miR-8084, hsa-miR-3609, hsa-miR-1260b, hsa-miR-5100,  hsa-miR-6507-3p, hsa-miR-3689f, hsa-miR-7-1-3p, hsa-miR-338-5p, hsa-miR-16-2-3p,  hsa-miR-579-5p, hsa-miR-4663, hsa-miR-4270, hsa-miR-330-5p, hsa-miR-342-5p,  hsa-miR-6825-5p, hsa-miR-204-3p, hsa-miR-4518, hsa-miR-659-3p, hsa-miR-335-5p,  hsa-miR-4635, hsa-miR-371b-5p, hsa-miR-6813-5p, hsa-miR-6785-5p, hsa-miR-4446-3p,  hsa-miR-3175, hsa-miR-635, hsa-miR-296-5p, hsa-miR-6774-5p, hsa-miR-5189-3p,  hsa-miR-4439, hsa-miR-1208, hsa-miR-3194-3p, hsa-miR-101-5p, hsa-miR-629-3p,  hsa-miR-885-3p, hsa-miR-4720-5p |
| **Bacterial invasion of epithelial cells** | ARPC5, ACTB, ARPC5L, MET, WASF1, ITGB1, WASL, CBL, CRKL, SEPT8, CRK, SHC1, PIK3CB, CAV1, SEPT11, SEPT1, PIK3R5, ACTG1, PXN, ITGA5, SHC2, ELMO2, CLTC, VCL, RHOA, CD2AP, CAV2, SHC3, ARPC1B, SEPT12, CTTN, PTK2, CBLB, HCLS1, CDH1, PIK3CD, PIK3R3, CTNNB1, CTNNA1, DOCK1, CLTA, ARHGAP10, ARPC2, WASF2, PIK3R1, ARPC1A, RAC1, CDC42, GAB1, SHC4, SEPT3, ELMO1, CTNNA3, PIK3CA, FN1, SEPT2, DNM3, SEPT6, CTNNA2, CLTB, ARHGEF26, SEPT9, RHOG, ILK, PIK3R2 | hsa-miR-4448, hsa-miR-6785-5p, hsa-miR-3175, hsa-miR-6825-5p, hsa-miR-4270,  hsa-miR-5088-5p, hsa-miR-6893-5p, hsa-miR-8063, hsa-miR-29c-3p, hsa-miR-6837-5p,  hsa-miR-3613-3p, hsa-miR-664b-3p, hsa-let-7f-1-3p, hsa-miR-6867-5p, hsa-miR-4663,  hsa-miR-548a-3p, hsa-miR-548ac, hsa-miR-548x-3p, hsa-miR-1468-3p, hsa-miR-152-3p,  hsa-miR-3148, hsa-miR-296-5p, hsa-miR-342-5p, hsa-miR-27a-3p, hsa-miR-371b-5p,  hsa-miR-4668-5p, hsa-miR-629-3p, hsa-miR-3609, hsa-miR-6507-3p, hsa-miR-5189-3p,  hsa-miR-7-5p, hsa-miR-574-5p, hsa-miR-635, hsa-miR-3194-3p, hsa-miR-6774-5p,  hsa-miR-16-2-3p, hsa-miR-195-3p, hsa-miR-204-3p, hsa-miR-7-1-3p, hsa-miR-98-5p,  hsa-miR-6813-5p, hsa-miR-330-5p, hsa-miR-4635, hsa-miR-4439, hsa-miR-34a-5p,  hsa-miR-3613-5p, hsa-miR-550a-3-5p, hsa-miR-338-5p, hsa-miR-4446-3p, hsa-miR-1208, hsa-miR-6886-5p, hsa-miR-3689f, hsa-miR-4518, hsa-miR-8084, hsa-miR-885-3p,  hsa-miR-3128, hsa-miR-6830-5p, hsa-miR-5100, hsa-miR-659-3p, hsa-miR-6744-5p |
| **Glutamatergic synapse** | SLC38A1, PRKCA, GRIN3A, CACNA1A, GNG13, ADCY1, ADCY5, ADRBK1, GNG11, ADCY7, ADRBK2, ADCY2, GNAS, TRPC1, GRM5, GNG12, PPP3R1, PLA2G4F, PLD1, SLC17A6, GRIA1, GRM3, GNAI3, GLS, GNB1, HOMER2, GRM6, GRIK5, GRM2, GRIA2, PPP3CA, PLCB1, GNB2, DLG4, PPP3CB, GRM1, PRKACG, PRKACA, DLGAP1, GNG7, SLC38A2, GLUL, GNG2, ITPR1, SLC17A7, PRKX, MAPK3, GRM7, PLA2G4D, PRKCB, GNAQ, GRIA4, GNAI2, GRIN2D, HOMER1, GNAO1, ADCY8, GRIK4, GNG4, SLC1A3, SHANK2, GRIK2, GRM4, GRIN1, PLA2G4E, ITPR3, CACNA1D, SLC1A6, GRM8, MAPK1, ITPR2, GNG5, GRIN2A, GLS2, ADCY9, ADCY4, GNB4, KCNJ3, PLCB4, PLCB2, GNB5, PLA2G4C, SLC1A2, GNAI1, PRKACB, PPP3R2, GRIA3, ADCY6, GRIK3, GRIN2B | hsa-miR-3613-3p, hsa-miR-3148, hsa-miR-548ac, hsa-miR-635, hsa-let-7f-1-3p,  hsa-miR-548x-3p, hsa-miR-7-1-3p, hsa-miR-3128, hsa-miR-6837-5p, hsa-miR-1468-3p,  hsa-miR-330-5p, hsa-miR-8063, hsa-miR-3175, hsa-miR-6825-5p, hsa-miR-4668-5p,  hsa-miR-548a-3p, hsa-miR-664b-3p, hsa-miR-27a-3p, hsa-miR-3613-5p, hsa-miR-5088-5p, hsa-miR-6744-5p, hsa-miR-7-5p, hsa-miR-29c-3p, hsa-miR-338-5p, hsa-miR-6867-5p,  hsa-miR-371b-5p, hsa-miR-6893-5p, hsa-miR-6787-5p, hsa-miR-6785-5p, hsa-miR-3194-3p, hsa-miR-98-5p, hsa-miR-296-5p, hsa-miR-6774-5p, hsa-miR-6830-5p, hsa-miR-574-5p,  hsa-miR-152-3p, hsa-miR-34a-5p, hsa-miR-6507-3p, hsa-miR-335-5p, hsa-miR-3609,  hsa-miR-8084, hsa-miR-204-3p, hsa-miR-6813-5p, hsa-miR-1208, hsa-miR-629-3p,  hsa-miR-3689f, hsa-miR-16-2-3p, hsa-miR-195-3p, hsa-miR-4635, hsa-miR-5189-3p,  hsa-miR-3201, hsa-miR-4446-3p, hsa-miR-659-3p, hsa-miR-4270, hsa-miR-342-5p,  hsa-miR-4448, hsa-miR-885-3p, hsa-miR-4423-3p, hsa-miR-101-5p, hsa-miR-550a-3-5p |
| **Endocytosis** | RNF41, VPS4A, IL2RG, HSPA2, RAB4A, SMAP2, ARAP2, PSD4, PDGFRA, TGFBR1, CHMP7, MET, PARD6G, ADRBK1, DNAJC6, SMAD2, SMAD6, CBL, STAM, CXCR4, VPS37D, ADRBK2, FGFR3, ARF5, SH3KBP1, GRK5, GRK7, VTA1, IQSEC3, ARF3, WWP1, DAB2, PRKCI, CYTH2, PDCD6IP, CAV1, FGFR4, HSPA1L, AP2B1, SMURF2, CHMP1B, NEDD4L, HLA-E, RAB7A, EEA1, EHD4, ITCH, SMAD3, CHMP4C, VPS36, ARFGAP3, PSD, HLA-B, SH3GL1, PLD1, GRK4, AGAP1, ADRB2, ACAP2, EPN2, IGF1R, EGFR, ARF1, ADRB3, CLTC, ARFGEF2, RHOA, SH3GLB1, CAV2, ERBB3, GRK1, RAB31, RET, VPS37B, TGFB1, MVB12B, PML, PIP5K1B, CHMP4B, RABEP1, HLA-F, RAB11FIP4, TSG101, CYTH3, ASAP1, RAB5A, ASAP3, VPS4B, CBLB, PSD3, SH3GL2, LDLRAP1, ZFYVE16, RAB11FIP2, CHMP2B, EPN1, ARFGAP2, GIT1, CLTA, GIT2, VPS37A, TRAF6, F2R, NTRK1, AGAP2, SMAP1, EPS15, TFRC, SH3GLB2, SMURF1, FLT1, CHMP5, RAB11A, KIT, RAB11FIP5, STAM2, IL2RA, PIP5K1A, SH3GL3, CDC42, AP2A1, USP8, CXCR1, NEDD4, CXCR2, TGFB2, GRK6, EPN3, CHMP3, ZFYVE20, STAMBP, CYTH1, VPS37C, RAB22A, ARF6, LDLR, PARD3, VPS28, DNM3, RAB11B, SMAD7, EHD2, VPS25, RAB5C, FGFR2, HSPA1B, IQSEC2, RAB11FIP1, CLTB, EHD3, KDR, PARD6B, RUFY1, IQSEC1, TGFBR2, ADRB1, RAB5B, MDM2, EGF, ERBB4, HLA-A, ARRB1, ASAP2 | hsa-miR-3613-3p, hsa-miR-98-5p, hsa-miR-664b-3p, hsa-miR-27a-3p, hsa-let-7f-1-3p,  hsa-miR-548x-3p, hsa-miR-8063, hsa-miR-548ac, hsa-miR-3194-3p, hsa-miR-7-1-3p,  hsa-miR-6867-5p, hsa-miR-3148, hsa-miR-1468-3p, hsa-miR-3609, hsa-miR-6744-5p,  hsa-miR-330-5p, hsa-miR-371b-5p, hsa-miR-4635, hsa-miR-4668-5p, hsa-miR-6787-5p,  hsa-miR-6785-5p, hsa-miR-6825-5p, hsa-miR-635, hsa-miR-7-5p, hsa-miR-6774-5p,  hsa-miR-548a-3p, hsa-miR-34a-5p, hsa-miR-296-5p, hsa-miR-152-3p, hsa-miR-6893-5p,  hsa-miR-4439, hsa-miR-5100, hsa-miR-8084, hsa-miR-204-3p, hsa-miR-4663, hsa-miR-4270, hsa-miR-29c-3p, hsa-miR-16-2-3p, hsa-miR-195-3p, hsa-miR-659-3p, hsa-miR-574-5p,  hsa-miR-338-5p, hsa-miR-3689f, hsa-miR-3128, hsa-miR-1208, hsa-miR-101-5p,  hsa-miR-4446-3p, hsa-miR-550a-3-5p, hsa-miR-6837-5p, hsa-miR-885-3p, hsa-miR-3613-5p, hsa-miR-342-5p, hsa-miR-629-3p, hsa-miR-3175, hsa-miR-5088-5p, hsa-miR-6813-5p,  hsa-miR-5189-3p, hsa-miR-4448, hsa-miR-4720-5p, hsa-miR-6830-5p, hsa-miR-6886-5p,  hsa-miR-4423-3p |
| **Glioma** | CAMK2D, BRAF, PRKCA, PDGFRA, E2F1 SOS2, CDK4, E2F2, NRAS, CALM3, CALM1, SHC1, PIK3CB, TGFA, PIK3R5, RAF1, SHC2, IGF1R, EGFR, KRAS, CDK6, SHC3, CALM2, CAMK2A, AKT2, PLCG1, PIK3CD, PIK3R3, CCND1, E2F3, AKT1, PDGFB, PIK3R1, RB1, SOS1, MAPK3, PLCG2, PRKCB, IGF1, SHC4, AKT3, CAMK2B, PIK3CA, CDKN1A, MAP2K1, MTOR, PTEN, MAPK1, GRB2, MDM2, EGF, PDGFA, PIK3R2 | hsa-miR-8084, hsa-miR-664b-3p, hsa-miR-27a-3p, hsa-miR-4439, hsa-miR-548x-3p,  hsa-miR-7-5p, hsa-miR-330-5p, hsa-miR-1208, hsa-miR-4635, hsa-miR-3613-3p,  hsa-miR-29c-3p, hsa-miR-548a-3p, hsa-miR-548ac, hsa-miR-3148, hsa-miR-7-1-3p,  hsa-miR-6744-5p, hsa-miR-8063, hsa-let-7f-1-3p, hsa-miR-34a-5p, hsa-miR-550a-3-5p,  hsa-miR-98-5p, hsa-miR-152-3p, hsa-miR-1468-3p, hsa-miR-204-3p, hsa-miR-6813-5p,  hsa-miR-885-3p, hsa-miR-335-5p, hsa-miR-6867-5p, hsa-miR-6893-5p, hsa-miR-3609,  hsa-miR-6825-5p, hsa-miR-6886-5p, hsa-miR-6507-3p, hsa-miR-3194-3p, hsa-miR-6785-5p, hsa-miR-371b-5p, hsa-miR-6837-5p, hsa-miR-659-3p, hsa-miR-4270, hsa-miR-338-5p,  hsa-miR-3128, hsa-miR-4668-5p, hsa-miR-6830-5p, hsa-miR-16-2-3p, hsa-miR-195-3p,  hsa-miR-342-5p, hsa-miR-5100, hsa-miR-629-3p, hsa-miR-4518, hsa-miR-3613-5p,  hsa-miR-3175, hsa-miR-4720-5p, hsa-miR-4663 |
| **Adrenergic signalling in cardiomyocytes** | CAMK2D, RAPGEF3, SCN7A, PRKCA, CACNG8, CACNA2D3, PPP2R5E, ATP1B2, ADCY1, ADCY5, ATF2, TPM1, ADCY7, CACNA1F, ATP2B2, PPP2R3A, ADCY2, CALM3, CALM1, AGTR1, GNAS, PPP2R2C, PIK3CB, PPP2CA, CREB5, ATP1A2, CACNB4, CACNG7, PIK3R5, PPP1CC, MAPK14, BCL2, ATP1B1, ADRB2, PPP2R5D, PPP2R2B, GNAI3, PPP2R2D, PPP2R5C, PPP1R1A, CREB3, CALM2, TPM3, SCN4B, ATP2B1, CREM, PPP2R5A, RPS6KA5, RAPGEF4, CREB1, CAMK2A, AKT2, TPM4, PLCB1, SLC9A1, PPP2R5B, ACTC1, PIK3CD, PIK3R3, ATP1B3, RYR2, PLN, AKT1, PPP2R2A, PPP2R1A, PRKACG, PRKACA, CACNB1, SLC8A1, CACNG4, PIK3R1, PRKX, PPP2R3C, ATP1B4, MAPK3, CACNA2D4, CACNA2D2, CACNG2, GNAQ, AKT3, CAMK2B, CREB3L2, GNAI2, PIK3CA, CREB3L1, ADCY8, AGTR2, ATP2B4, CACNG6, CACNA2D1, ATP2A2, CACNB2, ATP2B3, ATP1A1, CACNA1D, MAPK1, PPP2R1B, ADCY9, SCN1B, ADCY4, KCNQ1, ADRA1D, ADRB1, PLCB4, PLCB2, TPM2, GNAI1, PRKACB, PPP1CB, CACNB3, KCNE1, ADCY6, PIK3R2 | hsa-miR-6830-5p, hsa-miR-6825-5p, hsa-miR-4462, hsa-miR-4518, hsa-miR-204-3p,  hsa-miR-6813-5p, hsa-miR-330-5p, hsa-miR-1208, hsa-miR-3613-3p, hsa-miR-3148,  hsa-miR-548ac, hsa-miR-548x-3p, hsa-miR-3194-3p, hsa-miR-6785-5p, hsa-miR-548a-3p,  hsa-miR-1468-3p, hsa-miR-34a-5p, hsa-miR-664b-3p, hsa-miR-7-5p, hsa-miR-3689f,  hsa-miR-338-5p, hsa-miR-8063, hsa-miR-4668-5p, hsa-miR-659-3p, hsa-miR-4635,  hsa-miR-7-1-3p, hsa-miR-152-3p, hsa-miR-629-3p, hsa-miR-29c-3p, hsa-miR-6837-5p,  hsa-miR-574-5p, hsa-miR-371b-5p, hsa-miR-4446-3p, hsa-miR-6744-5p, hsa-miR-4663,  hsa-miR-3175, hsa-miR-98-5p, hsa-miR-296-5p, hsa-miR-6893-5p, hsa-miR-8084,  hsa-let-7f-1-3p, hsa-miR-4750-5p, hsa-miR-3128, hsa-miR-4720-5p, hsa-miR-4270,  hsa-miR-27a-3p, hsa-miR-3613-5p, hsa-miR-335-5p, hsa-miR-6867-5p, hsa-miR-342-5p,  hsa-miR-550a-3-5p, hsa-miR-16-2-3p, hsa-miR-195-3p, hsa-miR-885-3p, hsa-miR-4423-3p, hsa-miR-5100, hsa-miR-4439, hsa-miR-6886-5p, hsa-miR-5088-5p, hsa-miR-635,  hsa-miR-6774-5p |
| **Pancreatic cancer** | BRAF, STAT3, E2F1, TGFBR1, ERBB2, NFKB1, CDK4, SMAD2, E2F2, PIK3CB, TGFA, PIK3R5, RALA, RAF1, SMAD3, CHUK, RALBP1, PLD1, BRCA2, EGFR, ARHGEF6, KRAS, CDK6, TGFB1, IKBKB, AKT2, MAPK9, PIK3CD, PIK3R3, CCND1, SMAD4, E2F3, MAPK8, AKT1, PIK3R1, RB1, RAC1, MAPK3, CDC42, CASP9, TGFB2, BCL2L1, AKT3, PIK3CA, RALGDS, MAP2K1, RALB, STAT1, RELA, VEGFA, MAPK1, TGFBR2, JAK1, EGF, MAPK10, PIK3R2 | hsa-miR-3613-3p, hsa-miR-3148, hsa-miR-342-5p, hsa-miR-548a-3p, hsa-miR-548ac,  hsa-miR-664b-3p, hsa-miR-27a-3p, hsa-miR-548x-3p, hsa-miR-371b-5p, hsa-miR-8063,  hsa-miR-4668-5p, hsa-miR-1468-3p, hsa-miR-330-5p, hsa-miR-34a-5p, hsa-miR-4462,  hsa-miR-7-1-3p, hsa-let-7f-1-3p, hsa-miR-98-5p, hsa-miR-204-3p, hsa-miR-338-5p,  hsa-miR-3128, hsa-miR-152-3p, hsa-miR-1208, hsa-miR-4635, hsa-miR-885-3p,  hsa-miR-4446-3p, hsa-miR-7-5p, hsa-miR-6785-5p, hsa-miR-4270, hsa-miR-6830-5p,  hsa-miR-4439, hsa-miR-16-2-3p, hsa-miR-195-3p, hsa-miR-6837-5p, hsa-miR-6825-5p,  hsa-miR-635, hsa-miR-3194-3p, hsa-miR-6774-5p, hsa-miR-550a-3-5p, hsa-miR-6813-5p, hsa-miR-4448, hsa-miR-6893-5p, hsa-miR-8084, hsa-miR-29c-3p, hsa-miR-5100,  hsa-miR-629-3p, hsa-miR-4518, hsa-miR-6886-5p, hsa-miR-659-3p, hsa-miR-6744-5p,  hsa-miR-6867-5p, hsa-miR-3175, hsa-miR-3613-5p, hsa-miR-3689f |
| **Morphine addiction** | GABBR1, GABRA1, PDE1C, PRKCA, PDE4B, CACNA1A, GNG13, ADCY1, ADCY5, ADRBK1, GNG11, ADCY7, ADRBK2, GRK5, ADCY2, GNAS, GABRG2, PDE10A, PDE3A, GNG12, DRD1, GRK4, GNAI3, GNB1, PDE1B, PDE4D, PDE3B, PDE8B, ADORA1, PDE1A, GABBR2, GABRB3, GABRA3, GNB2, CACNA1B, GABRA5, PDE8A, PDE11A, PRKACG, PRKACA, GNG7, GABRG1, GNG2, PRKX, GABRA6, PDE2A, KCNJ6, PRKCB, GRK6, PDE4C, GNAI2, PDE7B, GNAO1, ADCY8, KCNJ9, GABRA4, GNG4, GABR, PDE7A, GABRB2, GNG5, GABRP, ADCY9, ADCY4, GNB4, KCNJ5, KCNJ3, GABRA2, GNB5, GNAI1, ARRB1, PDE4A, PRKACB, ADCY6 | hsa-miR-3148, hsa-miR-6825-5p, hsa-miR-7-5p, hsa-miR-6744-5p, hsa-miR-3613-3p,  hsa-miR-34a-5p, hsa-miR-664b-3p, hsa-miR-27a-3p, hsa-miR-6867-5p, hsa-miR-548x-3p,  hsa-miR-4635, hsa-miR-7-1-3p, hsa-miR-6893-5p, hsa-miR-4448, hsa-miR-5088-5p,  hsa-miR-548a-3p, hsa-miR-101-5p, hsa-miR-338-5p, hsa-miR-8063, hsa-miR-1468-3p,  hsa-miR-330-5p, hsa-miR-6785-5p, hsa-miR-371b-5p, hsa-miR-635, hsa-miR-6774-5p,  hsa-miR-342-5p, hsa-miR-548ac, hsa-let-7f-1-3p, hsa-miR-6830-5p, hsa-miR-4668-5p,  hsa-miR-3175, hsa-miR-574-5p, hsa-miR-204-3p, hsa-miR-3194-3p, hsa-miR-4423-3p,  hsa-miR-629-3p, hsa-miR-16-2-3p, hsa-miR-195-3p, hsa-miR-296-5p, hsa-miR-98-5p,  hsa-miR-4439, hsa-miR-4270, hsa-miR-6787-5p, hsa-miR-29c-3p, hsa-miR-3128,  hsa-miR-4518, hsa-miR-6837-5p, hsa-miR-550a-3-5p, hsa-miR-3689f, hsa-miR-885-3p,  hsa-miR-1208, hsa-miR-3613-5p, hsa-miR-152-3p, hsa-miR-5100, hsa-miR-6813-5p,  hsa-miR-6746-5p, hsa-miR-4446-3p, hsa-miR-659-3p, hsa-miR-8084 |
| **Phosphatidylinositol signalling system** | INPP5D, PIK3C3, PRKCA, CDS1, INPP5B, CDS2, PLCD1, DGKE, INPPL1, CALM3, CALM1, PIK3CB, INPP5J, PLCD3, PIK3R5, INPP5E, SYNJ1, IPPK, PI4KB, INPP4B, PIK3C2B, PLCZ1, DGKG, CALM2, PIP5K1B, PIP4K2A, IMPA2, PLCB1, PIKFYVE, PLCG1, PIK3CD, DGKZ, PIK3R3, IMPAD1, INPP4A, MTM1, ITPR1, PIK3R1, PIP5K1A, PI4K2A, PIK3C2A, PLCG2, INPP1, SYNJ2, PRKCB, DGKB, PLCE1, PIK3CA, OCRL, INPP5A, DGKD, ITPR3, PTEN, PI4K2B, ITPR2, PIP4K2C, DGKQ, DGKI, PLCB4, PLCB2, PIK3C2G, DGKH, PIK3R2 | hsa-miR-3613-3p, hsa-miR-204-3p, hsa-miR-6785-5p, hsa-miR-664b-3p, hsa-miR-6507-3p, hsa-miR-8063, hsa-miR-3201, hsa-miR-3148, hsa-miR-548a-3p, hsa-miR-1208,  hsa-miR-101-5p, hsa-miR-330-5p, hsa-miR-659-3p, hsa-miR-27a-3p, hsa-miR-8084,  hsa-miR-7-5p, hsa-miR-885-3p, hsa-miR-4446-3p, hsa-miR-5088-5p, hsa-miR-342-5p,  hsa-miR-548ac, hsa-miR-548x-3p, hsa-miR-371b-5p, hsa-miR-4635, hsa-miR-3613-5p,  hsa-miR-4423-3p, hsa-miR-16-2-3p, hsa-miR-195-3p, hsa-miR-629-3p, hsa-miR-6813-5p,  hsa-miR-1468-3p, hsa-miR-34a-5p, hsa-miR-4668-5p, hsa-miR-3194-3p, hsa-miR-4270,  hsa-miR-7-1-3p, hsa-miR-338-5p, hsa-miR-5100, hsa-miR-152-3p, hsa-miR-4663,  hsa-miR-29c-3p, hsa-miR-6830-5p, hsa-miR-3609, hsa-let-7f-1-3p, hsa-miR-6867-5p,  hsa-miR-6825-5p, hsa-miR-3175, hsa-miR-3689f, hsa-miR-6744-5p, hsa-miR-6893-5p,  hsa-miR-5189-3p, hsa-miR-335-5p, hsa-miR-574-5p, hsa-miR-1260b, hsa-miR-6837-5p |
| **Adipocytokine signalling pathway** | IRS2, ACSL5, LEP, STAT3, PRKCQ, NFKB1, SLC2A4, ACSL3, PRKAA2, CD36, RXRG, PCK2, ACSBG1, STK11, CHUK, CAMKK1, TNFRSF1B, ADIPOQ, ADIPOR2, CAMKK2, SOCS3, G6PC3, IKBKB, AKT2, G6PC, CPT1A, CPT1C, MAPK9, JAK2, PPARA, MAPK8, AKT1, ACSBG2, PRKAG1, NFKBIA, PTPN11, IRS1, PRKAA1, G6PC2, PRKAB2, AKT3, PPARGC1A, ACACB, PRKAG2, ACSL4, TRADD, TNFRSF1A, PRKAB1, ACSL1, SLC2A1, LEPR, MTOR, TNF, RELA, RXRB, ACSL6, PRKAG3, MAPK10 | hsa-miR-3148, hsa-miR-6825-5p, hsa-miR-548x-3p, hsa-miR-7-5p, hsa-miR-7-1-3p,  hsa-miR-34a-5p, hsa-miR-574-5p, hsa-miR-4635, hsa-miR-8063, hsa-miR-3613-3p,  hsa-miR-548ac, hsa-miR-3194-3p, hsa-miR-6886-5p, hsa-miR-548a-3p, hsa-miR-4720-5p, hsa-let-7f-1-3p, hsa-miR-371b-5p, hsa-miR-6744-5p, hsa-miR-330-5p, hsa-miR-29c-3p,  hsa-miR-5100, hsa-miR-629-3p, hsa-miR-98-5p, hsa-miR-152-3p, hsa-miR-335-5p,  hsa-miR-6837-5p, hsa-miR-1468-3p, hsa-miR-664b-3p, hsa-miR-3613-5p, hsa-miR-5088-5p, hsa-miR-1208, hsa-miR-6785-5p, hsa-miR-296-5p, hsa-miR-8084, hsa-miR-659-3p,  hsa-miR-4423-3p, hsa-miR-4668-5p, hsa-miR-342-5p, hsa-miR-6867-5p, hsa-miR-4439,  hsa-miR-338-5p, hsa-miR-6787-5p, hsa-miR-27a-3p, hsa-miR-635, hsa-miR-6774-5p,  hsa-miR-1260b, hsa-miR-204-3p, hsa-miR-885-3p, hsa-miR-4446-3p, hsa-miR-6813-5p,  hsa-miR-6830-5p, hsa-miR-550a-3-5p, hsa-miR-4270, hsa-miR-6893-5p, hsa-miR-5189-3p, hsa-miR-3689f, hsa-miR-101-5p |
| **Gap junction** | PRKCA, PDGFRA, GUCY1B3, ADCY1, ADCY5, SOS2, ADCY7, NRAS, ADCY2, GNAS, GUCY1A3, MAPK7, TUBA1B, GRM5, DRD1, RAF1, TUBB6, CDK1, EGFR, GNAI3, TJP1, KRAS, HTR2B, GNA11, TUBB4A, PLCB1, PRKG2, CSNK1D, DRD2, LPAR1, TUBA1C, TUBB2B, GRM1, TUBB2A, PDGFB, PRKACG, PRKACA, ITPR1, SOS1, GJD2, PRKX, MAPK3, PRKCB, TUBB3, GNAQ, PDGFC, GUCY1A2, PDGFD, GNAI2, ADCY8, GJA1, MAP2K1, MAP3K2, HTR2C, PRKG1, ITPR3, MAPK1, ITPR2, GRB2, ADCY9, ADCY4, ADRB1, EGF, PLCB4, PLCB2, GNAI1, PRKACB, HTR2A, PDGFA, ADCY6 | hsa-miR-6785-5p, hsa-miR-27a-3p, hsa-miR-548x-3p, hsa-miR-548a-3p, hsa-miR-1468-3p, hsa-miR-629-3p, hsa-miR-664b-3p, hsa-let-7f-1-3p, hsa-miR-7-5p, hsa-miR-8063,  hsa-miR-3613-3p, hsa-miR-29c-3p, hsa-miR-6830-5p, hsa-miR-548ac, hsa-miR-7-1-3p,  hsa-miR-8084, hsa-miR-4668-5p, hsa-miR-3148, hsa-miR-204-3p, hsa-miR-34a-5p,  hsa-miR-338-5p, hsa-miR-5088-5p, hsa-miR-335-5p, hsa-miR-3609, hsa-miR-659-3p,  hsa-miR-3613-5p, hsa-miR-6507-3p, hsa-miR-101-5p, hsa-miR-371b-5p, hsa-miR-4445-3p, hsa-miR-1208, hsa-miR-5100, hsa-miR-6744-5p, hsa-miR-195-3p, hsa-miR-550a-3-5p,  hsa-miR-4270, hsa-miR-885-3p, hsa-miR-5189-3p, hsa-miR-6893-5p, hsa-miR-152-3p,  hsa-miR-4518, hsa-miR-330-5p, hsa-miR-4635, hsa-miR-6825-5p, hsa-miR-6867-5p,  hsa-miR-98-5p, hsa-miR-3689f, hsa-miR-3194-3p, hsa-miR-574-5p, hsa-miR-6837-5p,  hsa-miR-4439, hsa-miR-4423-3p, hsa-miR-4446-3p, hsa-miR-16-2-3p, hsa-miR-3175,  hsa-miR-635, hsa-miR-296-5p, hsa-miR-6774-5p, hsa-miR-6813-5p |
| **Oxytocin signalling pathway** | CAMK2D, ACTB, FOS, PRKCA, CACNG8, EEF2K, GUCY1B3, CACNA2D3, MYLK4, ADCY1, ADCY5, ROCK1, CAMK4, ADCY7, NRAS, PRKAA2, CACNA1F, CD38, ADCY2, RCAN1, CALM3, CALM1, GNAS, GUCY1A3, PIK3CB, MAPK7, CACNB4, CACNG7, PIK3R5, PPP1CC, ACTG1, ROCK2, PPP3R1, PLA2G4F, RAF1, CAMKK1, EGFR, MYLK2, GNAI3, RHOA, PPP1R12B, KRAS, CALM2, CAMKK2, NFATC4, CAMK2A, PPP3CA, MYL6B, RYR1, PLCB1, PPP1R12A, PIK3CD, PIK3R3, CCND1, PPP3CB, RYR2, NFATC2, PRKAG1, PRKACG, PRKACA, CACNB1, PTGS2, CACNG4, ITPR1, PIK3R1, PRKX, OXTR, MAPK3, PRKAA1, CAMK1, CACNA2D4, RYR3, KCNJ6, CACNA2D2, PLA2G4D, CACNG2, PRKCB, PRKAB2, KCNJ2, GNAQ, MYLK3, CAMK2B, PRKAG2, GUCY1A2, GNAI2, PIK3CA, GNAO1, ADCY8, PRKAB1, KCNJ9, CDKN1A, MAP2K1, CACNG6, CACNA2D1, CACNB2, MEF2C, CAMK1D, PLA2G4E, ITPR3, CACNA1D, NFATC3, MAPK1, ITPR2, NFATC1, ADCY9, ADCY4, CAMK1G, KCNJ5, KCNJ3, ELK1, PLCB4, PLCB2, MYLK, PRKAG3, PLA2G4C, GNAI1, PRKACB, PPP3R2, PPP1CB, CACNB3, ADCY6, PIK3R2 | hsa-miR-3613-3p, hsa-miR-4668-5p, hsa-miR-152-3p, hsa-miR-548ac, hsa-miR-664b-3p,  hsa-miR-548x-3p, hsa-miR-7-1-3p, hsa-miR-8063, hsa-miR-8084, hsa-miR-34a-5p,  hsa-miR-3194-3p, hsa-miR-4270, hsa-miR-296-5p, hsa-miR-4423-3p, hsa-miR-3148,  hsa-miR-29c-3p, hsa-miR-548a-3p, hsa-miR-1468-3p, hsa-miR-330-5p, hsa-miR-6785-5p,  hsa-miR-27a-3p, hsa-miR-635, hsa-miR-659-3p, hsa-miR-574-5p, hsa-miR-4635,  hsa-miR-371b-5p, hsa-miR-338-5p, hsa-miR-4446-3p, hsa-miR-550a-3-5p, hsa-miR-6893-5p, hsa-let-7f-1-3p, hsa-miR-6774-5p, hsa-miR-5088-5p, hsa-miR-6744-5p, hsa-miR-6825-5p, hsa-miR-7-5p, hsa-miR-3128, hsa-miR-6837-5p, hsa-miR-98-5p, hsa-miR-3609,  hsa-miR-204-3p, hsa-miR-5189-3p, hsa-miR-342-5p, hsa-miR-4663, hsa-miR-6867-5p,  hsa-miR-3689f, hsa-miR-629-3p, hsa-miR-16-2-3p, hsa-miR-195-3p, hsa-miR-1208,  hsa-miR-6830-5p, hsa-miR-3613-5p, hsa-miR-6813-5p, hsa-miR-3175, hsa-miR-4720-5p,  hsa-miR-335-5p, hsa-miR-6507-3p, hsa-miR-101-5p, hsa-miR-4439 |
| **MAPK signalling pathway** | TAOK3, BRAF, DUSP4, HSPA2, FGF12, FOS, NTRK2, PRKCA, NTF3, CACNG8, PDGFRA, CACNA1A, TGFBR1, CACNA2D3, NFKB1, CACNA1G, IL1R1, SOS2, MAP4K2, MAPK8IP2, ATF2, FLNC, FGF14, PPP5C, NRAS, FGFR3, CACNA1F, DUSP2, CRKL, STK4, RASA2, ELK4, FGF13, MAP3K3, CRK, CDC25B, MAPK7, PAK2, MAP2K7, FGFR4, CACNB4, HSPA1L, CACNG7, RAP1A, DUSP6, MAPK14, PTPRR, RASGRF2, GNG12, LAMTOR3, FGF10, PPP3R1, PLA2G4F, RAF1, CHUK, MAP4K3, MAP4K4, MAP2K3, RPS6KA1, EGFR, GADD45A, MRAS, FGF20, MAP3K4, MAP3K1, MAP3K13, TAB2, KRAS, TGFB1, BDNF, RRAS2, FASLG, TAOK1, RPS6KA5, PAK1, MAP2K6, HSPB1, CACNA1I, IKBKB, FGF11, PPP3CA, AKT2, NLK, DUSP10, RASA1, MAP3K12, MAPK9, CACNA1B, CASP3, PPP3CB, MAPKAPK5, RASGRP1, PPP5D1, RAPGEF2, MAPK8, TRAF6, AKT1, MYC, NTRK1, MAP4K1, PPM1A, RASGRP4, FLNB, MAPKAPK3, ZAK, PDGFB, PRKACG, PRKACA, CACNB1, FLNA, CACNA1E, CACNG4, SOS1, FGF9, PRKX, RAC1, DUSP8, MAPK3, FGF2, CDC42, STK3, CACNA2D4, FGF18, CACNA2D2, PLA2G4D, NF1, CACNG2, NGF, FGF5, DUSP3, PRKCB, MAX, FAS, RASGRF1, RPS6KA3, TGFB2, AKT3, JUND, TNFRSF1A, MECOM, FGF8, MAP2K1, STMN1, MAP3K2, MKNK1, MAP3K8, RASGRP3, PPM1B, CACNG6, CACNA2D1, CACNB2, PTPN5, MEF2C, FGF23, MKNK2, DUSP5, DUSP9, FGFR2, HSPA1B, IL1A, MAP2K4, TNF, PLA2G4E, RELA, CACNA1D, NFATC3, FGFR1, DUSP16, FGF16, MAPK1, MAP3K7, FGF1, SRF, NFATC1, FGF7, GRB2, RAP1B, MAPKAPK2, DUSP1, TGFBR2, ELK1, EGF, PLA2G4C, MAP3K5, RRAS, MAPK10, ARRB1, PRKACB, PPP3R2, CACNB3, PDGFA, RPS6KA2 | hsa-miR-3613-3p, hsa-miR-8084, hsa-miR-27a-3p, hsa-let-7f-1-3p, hsa-miR-548x-3p,  hsa-miR-1208, hsa-miR-7-1-3p, hsa-miR-3148, hsa-miR-548a-3p, hsa-miR-548ac,  hsa-miR-5189-3p, hsa-miR-34a-5p, hsa-miR-664b-3p, hsa-miR-6867-5p, hsa-miR-7-5p,  hsa-miR-4635, hsa-miR-6893-5p, hsa-miR-4446-3p, hsa-miR-204-3p, hsa-miR-338-5p,  hsa-miR-659-3p, hsa-miR-6837-5p, hsa-miR-6830-5p, hsa-miR-330-5p, hsa-miR-3194-3p, hsa-miR-342-5p, hsa-miR-6886-5p, hsa-miR-152-3p, hsa-miR-1468-3p, hsa-miR-6785-5p, hsa-miR-296-5p, hsa-miR-635, hsa-miR-6774-5p, hsa-miR-6825-5p, hsa-miR-5088-5p,  hsa-miR-6744-5p, hsa-miR-8063, hsa-miR-3175, hsa-miR-5100, hsa-miR-29c-3p,  hsa-miR-4663, hsa-miR-3689f, hsa-miR-4439, hsa-miR-4270, hsa-miR-629-3p,  hsa-miR-98-5p, hsa-miR-3613-5p, hsa-miR-550a-3-5p, hsa-miR-4668-5p, hsa-miR-6813-5p, hsa-miR-335-5p, hsa-miR-371b-5p, hsa-miR-3128, hsa-miR-4720-5p, hsa-miR-16-2-3p,  hsa-miR-195-3p, hsa-miR-574-5p, hsa-miR-101-5p, hsa-miR-4462, hsa-miR-6507-3p,  hsa-miR-885-3p, hsa-miR-6787-5p, hsa-miR-3609, hsa-miR-3605-3p, hsa-miR-4785,  hsa-miR-4423-3p |
| **cAMP signalling pathway** | SSTR1, GABBR1, CAMK2D, BRAF, RAPGEF3, FOS, GRIN3A, HTR1D, PDE4B, NFKB1, ATP1B2, ADCY1, ADCY5, ROCK1, ACOX1, CAMK4, ADCY7, CACNA1F, ATP2B2, ADCY2, CNGA4, CALM3, CALM1, GNAS, PIK3CB, PTCH1, MC2R, CREB5, ATP1A2, FSHR, HHIP, PIK3R5, PPP1CC, RAP1A, HTR1E, ACOX3, ROCK2, PDE3A, DRD1, RAF1, ADCY10, HTR1F, PLD1, SUCNR1, TIAM1, GRIA1, ATP1B1, ADRB2, GNAI3, RHOA, BDNF, CREB3, CALM2, RRAS2, ABCC4, NPY1R, PTGER3, ATP2B1, ADORA2A, PAK1, PDE4D, PDE3B, RAPGEF4, VAV2, CREB1, GRIA2, CAMK2A, ADORA1, GABBR2, MLLT4, AKT2, SLC9A1, VIPR2, PPP1R12A, MAPK9, DRD2, PIK3CD, PIK3R3, PPARA, ATP1B3, CNGB3, SOX9, RYR2, PLN, MAPK8, AKT1, F2R, SSTR2, PTGER2, GLI3, TSHR, PRKACG, PRKACA, NFKBIA, PIK3R1, PRKX, OXTR, RAC1, ATP1B4, MAPK3, CNGA2, CHRM2, FSHB, CNGA1, DRD5, EP300, AKT3, CAMK2B, PDE4C, CREB3L2, HCAR1, PLCE1, GRIA4, GNAI2, PIK3CA, GLP1R, GRIN2D, CREB3L1, ADCY8, MAP2K1, ATP2B4, CNGB1, EDNRA, ATP2A2, PPP1R1B, GRIN1, ATP2B3, ATP1A1, RELA, VAV3, CACNA1D, ADCYAP1R1, MAPK1, CREBBP, NFATC1, GRIN2A, ADCY9, ADCY4, RAP1B, ADRB1, HCN4, RRAS, MAPK10, GNAI1, PDE4A, PRKACB, SSTR5, PPP1CB, GRIA3, CHRM1, ADCY6, GRIN2B, PIK3R2 | hsa-miR-3148, hsa-miR-27a-3p, hsa-miR-3613-3p, hsa-miR-548ac, hsa-miR-548x-3p,  hsa-miR-1468-3p, hsa-miR-664b-3p, hsa-miR-6744-5p, hsa-miR-8063, hsa-miR-8084,  hsa-miR-6886-5p, hsa-miR-5100, hsa-miR-6825-5p, hsa-miR-7-5p, hsa-miR-204-3p,  hsa-miR-3689f, hsa-miR-152-3p, hsa-miR-7-1-3p, hsa-miR-338-5p, hsa-miR-98-5p,  hsa-miR-4270, hsa-miR-885-3p, hsa-miR-6867-5p, hsa-miR-4750-5p, hsa-miR-6893-5p,  hsa-miR-29c-3p, hsa-miR-548a-3p, hsa-miR-4668-5p, hsa-miR-1208, hsa-miR-635,  hsa-miR-6774-5p, hsa-miR-34a-5p, hsa-miR-371b-5p, hsa-miR-574-5p, hsa-miR-4448,  hsa-miR-5088-5p, hsa-miR-659-3p, hsa-let-7f-1-3p, hsa-miR-6785-5p, hsa-miR-6837-5p,  hsa-miR-550a-3-5p, hsa-miR-16-2-3p, hsa-miR-195-3p, hsa-miR-3201, hsa-miR-330-5p,  hsa-miR-4446-3p, hsa-miR-3194-3p, hsa-miR-5189-3p, hsa-miR-629-3p, hsa-miR-6830-5p, hsa-miR-4635, hsa-miR-296-5p, hsa-miR-3175, hsa-miR-3609, hsa-miR-4423-3p,  hsa-miR-342-5p, hsa-miR-4720-5p, hsa-miR-335-5p, hsa-miR-3128, hsa-miR-4518,  hsa-miR-4439, hsa-miR-3613-5p, hsa-miR-6813-5p, hsa-miR-4663, hsa-miR-101-5p |
| **Platelet activation** | ACTB, SNAP23, TLN2, GUCY1B3, MYLK4, GP5, PTGIR, ADCY1, ADCY5, ITGB1, ROCK1, ADCY7, ADCY2, GNAS, GUCY1A3, PIK3CB, MYL12B, PRKCI, SYK, ARHGEF12, COL24A1, PIK3R5, PPP1CC, RAP1A, MAPK14, GNA13, COL27A1, ACTG1, PTGS1, ROCK2, PLA2G4F, MYLK2, GNAI3, F2RL3, RHOA, COL3A1, FYN, ARHGEF1, AKT2, ARHGAP35, PLCB1, PRKG2, COL2A1, PPP1R12A, GP1BA, PIK3CD, PIK3R3, RASGRP1, AKT1, F2R, COL5A1, PRKACG, COL1A1, PRKACA, FCER1G, ITGA2, ITPR1, PIK3R1, PRKX, P2RX1, STIM1, COL11A2, MAPK3, P2RY1, PLCG2, PLA2G4D, COL1A2, GNAQ, AKT3, MYLK3, COL11A1, GUCY1A2, GNAI2, PIK3CA, VASP, ADCY8, FGA, APBB1IP, PRKG1, PLA2G4E, LCP2, ITPR3, COL5A3, MAPK1, TLN1, ITPR2, ADCY9, ADCY4, RAP1B, COL5A2, PLCB4, PLCB2, MYLK, ITGA2B, PLA2G4C, SEPT5, GNAI1, PRKACB, TBXA2R, PPP1CB, ADCY6, ITGB3, PIK3R2 | hsa-miR-548x-3p, hsa-miR-6825-5p, hsa-miR-5189-3p, hsa-miR-27a-3p, hsa-let-7f-1-3p,  hsa-miR-16-2-3p, hsa-miR-195-3p, hsa-miR-7-1-3p, hsa-miR-338-5p, hsa-miR-8063,  hsa-miR-98-5p, hsa-miR-29c-3p, hsa-miR-6785-5p, hsa-miR-6830-5p, hsa-miR-4635,  hsa-miR-629-3p, hsa-miR-4668-5p, hsa-miR-3613-3p, hsa-miR-330-5p, hsa-miR-548a-3p, hsa-miR-548ac, hsa-miR-1468-3p, hsa-miR-4270, hsa-miR-4663, hsa-miR-34a-5p,  hsa-miR-664b-3p, hsa-miR-3194-3p, hsa-miR-7-5p, hsa-miR-6744-5p, hsa-miR-4446-3p,  hsa-miR-6867-5p, hsa-miR-5088-5p, hsa-miR-6893-5p, hsa-miR-3148, hsa-miR-8084,  hsa-miR-6886-5p, hsa-miR-4448, hsa-miR-204-3p, hsa-miR-4720-5p, hsa-miR-296-5p,  hsa-miR-3689f, hsa-miR-885-3p, hsa-miR-635, hsa-miR-6774-5p, hsa-miR-574-5p,  hsa-miR-6813-5p, hsa-miR-1208, hsa-miR-152-3p, hsa-miR-659-3p, hsa-miR-3613-5p,  hsa-miR-342-5p, hsa-miR-3609, hsa-miR-6837-5p, hsa-miR-335-5p, hsa-miR-6507-3p,  hsa-miR-101-5p, hsa-miR-371b-5p, hsa-miR-5100, hsa-miR-550a-3-5p, hsa-miR-3175,  hsa-miR-3128, hsa-miR-4423-3p |
| **Circadian rhythm** | FBXL3, PRKAA2, BTRC, RORC, RORB, CUL1, PER3, BHLHE41, SKP1, CREB1, NPAS2, ARNTL, CSNK1D, CRY2, BHLHE40, PRKAG1, RORA, PRKAA1, PRKAB2, CSNK1E, PRKAG2, RBX1, NR1D1, PRKAB1, PER2, CRY1, FBXW11, PRKAG3, CLOCK | hsa-let-7f-1-3p, hsa-miR-3613-3p, hsa-miR-4668-5p, hsa-miR-152-3p, hsa-miR-548ac,  hsa-miR-664b-3p, hsa-miR-548x-3p, hsa-miR-7-1-3p, hsa-miR-8063, hsa-miR-3128,  hsa-miR-3148, hsa-miR-6825-5p, hsa-miR-6787-5p, hsa-miR-629-3p, hsa-miR-338-5p,  hsa-miR-4518, hsa-miR-6785-5p, hsa-miR-4663, hsa-miR-885-3p, hsa-miR-6893-5p,  hsa-miR-1468-3p, hsa-miR-6830-5p, hsa-miR-548a-3p, hsa-miR-1208, hsa-miR-8084,  hsa-miR-98-5p, hsa-miR-29c-3p, hsa-miR-34a-5p, hsa-miR-550a-3-5p, hsa-miR-3613-5p,  hsa-miR-5189-3p, hsa-miR-16-2-3p, hsa-miR-195-3p, hsa-miR-4635, hsa-miR-27a-3p,  hsa-miR-4720-5p, hsa-miR-4270, hsa-miR-371b-5p, hsa-miR-659-3p, hsa-miR-204-3p,  hsa-miR-6813-5p, hsa-miR-330-5p |
| **AMPK signalling pathway** | IRS2, LEP, RAB8A, TSC1, EEF2K, PPP2R5E, SLC2A4, HNF4A, SCD5, PRKAA2, CD36, PPP2R3A, CCNA2, HMGCR, SIRT1, PPP2R2C, PIK3CB, PPP2CA, CREB5, PIK3R5, PCK2, STK11, RAB2A, PPP2R5D, PPP2R2B, CAMKK1, IGF1R, PPP2R2D, PPP2R5C, RHEB, CREB3, SREBF1, ADIPOQ, ADIPOR2, CAMKK2, RPS6KB2, PPP2R5A, G6PC3, CREB1, AKT2, G6PC, AKT1S1, CPT1A, CPT1C, CRTC2, PPP2R5B, TSC2, PIK3CD, PIK3R3, CCND1, TBC1D1, AKT1, PPP2R2A, PRKAG1, PPP2R1A, GYS2, PIK3R1, PPP2R3C, IRS1, INSR, PRKAA1, ELAVL1, G6PC2, PRKAB2, IGF1, PPARG, AKT3, CREB3L2, PPARGC1A, ACACB, PRKAG2, PIK3CA, PFKM, SCD, CREB3L1, PRKAB1, FOXO3, PDPK1, PFKFB2, RAB11B, LEPR, MTOR, ULK1, FOXO1, CAB39, MAP3K7, PPP2R1B, RAB10, PFKFB4, RAB14, STRADA, CAB39L, PRKAG3, RPS6KB1, PFKFB3, PIK3R2 | hsa-miR-3613-3p, hsa-miR-29c-3p, hsa-miR-548a-3p, hsa-miR-548ac, hsa-miR-664b-3p,  hsa-miR-548x-3p, hsa-miR-3148, hsa-miR-6785-5p, hsa-miR-4668-5p, hsa-miR-3689f,  hsa-miR-629-3p, hsa-miR-338-5p, hsa-miR-8063, hsa-miR-330-5p, hsa-miR-6830-5p,  hsa-miR-4635, hsa-miR-6744-5p, hsa-miR-3128, hsa-miR-4448, hsa-miR-1468-3p,  hsa-miR-98-5p, hsa-miR-885-3p, hsa-let-7f-1-3p, hsa-miR-3613-5p, hsa-miR-7-1-3p,  hsa-miR-6825-5p, hsa-miR-7-5p, hsa-miR-4462, hsa-miR-4518, hsa-miR-152-3p,  hsa-miR-27a-3p, hsa-miR-635, hsa-miR-6774-5p, hsa-miR-574-5p, hsa-miR-371b-5p,  hsa-miR-4439, hsa-miR-1208, hsa-miR-6813-5p, hsa-miR-6837-5p, hsa-miR-34a-5p,  hsa-miR-3194-3p, hsa-miR-4270, hsa-miR-8084, hsa-miR-335-5p, hsa-miR-5088-5p,  hsa-miR-16-2-3p, hsa-miR-195-3p, hsa-miR-6893-5p, hsa-miR-550a-3-5p, hsa-miR-5100,  hsa-miR-4720-5p, hsa-miR-6886-5p, hsa-miR-659-3p, hsa-miR-4663, hsa-miR-6867-5p,  hsa-miR-1260b, hsa-miR-342-5p, hsa-miR-204-3p, hsa-miR-3175, hsa-miR-4446-3p,  hsa-miR-296-5p, hsa-miR-5189-3p |
| **Neurotrophin signalling pathway** | CAMK2D, BRAF, GSK3B, NTRK2, NTF3, NFKB1, ZNF274, SOS2, RIPK2, RAPGEF1, SH2B3, CAMK4, NRAS, CRKL, YWHAE, IRAK3, CALM3, CALM1, MAP3K3, CRK, SHC1, PIK3CB, MAPK7, MAP2K7, SORT1, PIK3R5, RAP1A, MAPK14, BAX, NTRK3, FRS2, RAF1, BCL2, RPS6KA1, SHC2, MAP3K1, RHOA, NGFRAP1, MAGED1, KRAS, SHC3, BDNF, CALM2, ARHGDIB, FASLG, RPS6KA5, IKBKB, CAMK2A, AKT2, MAPK9, PLCG1, PIK3CD, PIK3R3, TP73, MAPK8, TRAF6, AKT1, KIDINS220, NTRK1, NFKBIA, ARHGDIA, PIK3R1, SOS1, PTPN11, IRS1, RAC1, MAPK3, CDC42, PLCG2, PSEN1, NGF, RPS6KA3, GAB1, SHC4, AKT3, CAMK2B, PIK3CA, FOXO3, MAP2K1, IRAK2, PDPK1, SH2B1, PRDM4, RELA, IRAK1, IRAK4, MAPK1, GRB2, RAP1B, MAPKAPK2, NGFR, ABL1, MAP3K5, MAPK10, RPS6KA2, PIK3R2 | hsa-miR-3613-3p, hsa-miR-8084, hsa-miR-3148, hsa-miR-548ac, hsa-miR-6867-5p,  hsa-miR-548x-3p, hsa-miR-8063, hsa-miR-98-5p, hsa-miR-659-3p, hsa-miR-152-3p,  hsa-miR-1468-3p, hsa-miR-330-5p, hsa-miR-27a-3p, hsa-miR-6507-3p, hsa-miR-4635,  hsa-miR-7-1-3p, hsa-miR-4668-5p, hsa-miR-664b-3p, hsa-miR-4439, hsa-miR-6785-5p,  hsa-miR-6825-5p, hsa-miR-4785, hsa-miR-4270, hsa-let-7f-1-3p, hsa-miR-7-5p,  hsa-miR-3128, hsa-miR-3609, hsa-miR-1260b, hsa-miR-1208, hsa-miR-885-3p,  hsa-miR-29c-3p, hsa-miR-6893-5p, hsa-miR-548a-3p, hsa-miR-34a-5p, hsa-miR-3194-3p,  hsa-miR-6744-5p, hsa-miR-6837-5p, hsa-miR-4446-3p, hsa-miR-296-5p, hsa-miR-5088-5p, hsa-miR-342-5p, hsa-miR-6886-5p, hsa-miR-3613-5p, hsa-miR-4663, hsa-miR-371b-5p,  hsa-miR-635, hsa-miR-6774-5p, hsa-miR-6830-5p, hsa-miR-629-3p, hsa-miR-16-2-3p,  hsa-miR-195-3p, hsa-miR-338-5p, hsa-miR-335-5p, hsa-miR-204-3p, hsa-miR-3175,  hsa-miR-4720-5p, hsa-miR-4423-3p, hsa-miR-550a-3-5p, hsa-miR-6813-5p, hsa-miR-574-5p, hsa-miR-4462, hsa-miR-5100, hsa-miR-5189-3p, hsa-miR-101-5p, hsa-miR-3689f |
| **FoxO signalling pathway** | IRS2, BRAF, FBXO25, RBL2, STAT3, RAG1, TGFBR1, FBXO32, SLC2A4, SOS2, SMAD2, NRAS, PRKAA2, STK4, GABARAPL2, SIRT1, PIK3CB, SETD7, KLF2, PIK3R5, MAPK14, CDK2, CCND2, PCK2, STK11, RAF1, SMAD3, CHUK, FOXO4, CDKN1B, CDKN2B, IGF1R, EGFR, GADD45A, KRAS, HOMER2, TGFB1, FASLG, IL7R, G6PC3, IKBKB, AKT2, ATM, NLK, G6PC, RAG2, MAPK9, PIK3CD, PIK3R3, CCND1, SMAD4, S1PR1, SKP2, MAPK8, AKT1, GRM1, PRKAG1, PLK2, FOXG1, AGAP2, PIK3R1, SOS1, IRS1, INSR, MAPK3, PRKAA1, BCL6, G6PC2, PRKAB2, IGF1, TGFB2, CSNK1E, EP300, AKT3, SOD2, PRKAG2, PIK3CA, HOMER1, SGK2, PRKAB1, FOXO3, USP7, CDKN1A, MAP2K1, ATG12, PDPK1, PTEN, SGK3, FOXO1, PLK4, CDKN2D, MAPK1, CREBBP, IL10, GRB2, CCNG2, TGFBR2, SGK1, MDM2, EGF, PRKAG3, BCL2L11, MAPK10, PIK3R2, C8orf44-SGK3 | hsa-miR-3613-3p, hsa-miR-152-3p, hsa-miR-548ac, hsa-miR-548x-3p, hsa-miR-659-3p,  hsa-miR-8063, hsa-miR-3148, hsa-miR-16-2-3p, hsa-miR-195-3p, hsa-miR-7-1-3p,  hsa-miR-548a-3p, hsa-miR-664b-3p, hsa-miR-27a-3p, hsa-miR-338-5p, hsa-miR-6893-5p, hsa-miR-4270, hsa-miR-98-5p, hsa-miR-29c-3p, hsa-miR-1468-3p, hsa-miR-8084,  hsa-miR-4439, hsa-miR-7-5p, hsa-miR-3128, hsa-miR-1208, hsa-miR-4668-5p,  hsa-miR-629-3p, hsa-miR-6785-5p, hsa-miR-342-5p, hsa-miR-371b-5p, hsa-miR-330-5p,  hsa-miR-4635, hsa-let-7f-1-3p, hsa-miR-6825-5p, hsa-miR-3609, hsa-miR-6867-5p,  hsa-miR-34a-5p, hsa-miR-6830-5p, hsa-miR-296-5p, hsa-miR-635, hsa-miR-6774-5p,  hsa-miR-885-3p, hsa-miR-3613-5p, hsa-miR-6886-5p, hsa-miR-5100, hsa-miR-3194-3p,  hsa-miR-574-5p, hsa-miR-4448, hsa-miR-6507-3p, hsa-miR-4423-3p, hsa-miR-4663,  hsa-miR-204-3p, hsa-miR-6813-5p, hsa-miR-3689f, hsa-miR-6744-5p, hsa-miR-6837-5p,  hsa-miR-4462, hsa-miR-4720-5p, hsa-miR-101-5p, hsa-miR-550a-3-5p |
| **Small cell lung cancer** | LAMB2, E2F1, NFKB1, ITGB1, CDK4, LAMB1, E2F2, PIK3CB, RXRG, COL4A5, PIK3R5, CDK2, ITGA3, CHUK, LAMA1, RARB, BCL2, CDKN1B, TRAF4, CDKN2B, TRAF5, CDK6, IKBKB, APAF1, AKT2, PTK2, ITGAV, PIK3CD, PIK3R3, CCND1, CCNE2, SKP2, COL4A2, E2F3, TRAF6, AKT1, MYC, NFKBIA, PTGS2, COL4A3, ITGA2, PIK3R1, RB1, COL4A4, NOS2, CASP9, MAX, LAMC1, BCL2L1, AKT3, PIAS2, PIK3CA, COL4A6, FN1, BIRC3, LAMA2, CKS2, LAMC2, CYCS, ITGA6, RELA, RXRB, PTEN, TRAF3, TRAF1, ITGA2B, XIAP, COL4A1, LAMA4, PIK3R2 | hsa-miR-3613-3p, hsa-miR-3148, hsa-miR-548x-3p, hsa-miR-7-1-3p, hsa-miR-7-5p,  hsa-miR-885-3p, hsa-miR-29c-3p, hsa-miR-3128, hsa-miR-34a-5p, hsa-miR-548ac,  hsa-miR-664b-3p, hsa-miR-8063, hsa-miR-8084, hsa-miR-4668-5p, hsa-miR-27a-3p,  hsa-miR-629-3p, hsa-miR-330-5p, hsa-miR-3689f, hsa-miR-3201, hsa-miR-548a-3p,  hsa-miR-1468-3p, hsa-miR-6893-5p, hsa-miR-6837-5p, hsa-let-7f-1-3p, hsa-miR-1208,  hsa-miR-4446-3p, hsa-miR-3613-5p, hsa-miR-16-2-3p, hsa-miR-195-3p, hsa-miR-4635,  hsa-miR-338-5p, hsa-miR-6830-5p, hsa-miR-152-3p, hsa-miR-6785-5p, hsa-miR-371b-5p, hsa-miR-98-5p, hsa-miR-6825-5p, hsa-miR-635, hsa-miR-3194-3p, hsa-miR-6774-5p,  hsa-miR-6744-5p, hsa-miR-3609, hsa-miR-659-3p, hsa-miR-6507-3p, hsa-miR-5088-5p,  hsa-miR-204-3p, hsa-miR-4663, hsa-miR-4439, hsa-miR-342-5p, hsa-miR-4270,  hsa-miR-6886-5p, hsa-miR-6867-5p |
| **Regulation of actin cytoskeleton** | ARPC5, PFN1, BRAF, ACTB, FGF12, PDGFRA, ACTN2, BDKRB1, MYLK4, ARPC5L, WASF1, FGD1, SSH2, ITGB1, CYFIP2, EZR, CHRM5, ROCK1, WASL, ITGAX, SOS2, ITGB8, ITGA9, FGF14, NRAS, FGFR3, CRKL, FGF13, APC, CRK, SSH1, CFL1, IQGAP3, ITGA8, PIK3CB, MYL12B, PAK2, FGFR4, ARHGEF12, PIK3R5, PPP1CC, GNA13, ACTG1, PXN, ROCK2, ITGB6, BDKRB2, MYH10, PAK7, GNG12, TMSB4Y, FGF10, RDX, IQGAP1, ITGA5, RAF1, ITGA3, MYH9, INSRR, TIAM1, CHRM3, EGFR, MYLK2, MRAS, VCL, FGF20, RHOA, PPP1R12B, ITGA1, ARHGEF6, KRAS, PAK3, ARPC1B, RRAS2, MSN, PIP5K1B, ABI2, PIP4K2A, ARHGEF1, PAK1, VAV2, FGF11, ARHGAP35, PTK2, ITGAV, ITGA11, GSN, SLC9A1, MYH14, PIKFYVE, PPP1R12A, TMSB4X, PIK3CD, PIK3R3, PFN2, GIT1, DOCK1, F2R, LIMK2, IQGAP2, NCKAP1L, DIAPH1, FGD3, ARPC2, ITGAM, PDGFB, WASF2, DIAPH3, ITGA2, CFL2, PIK3R1, SOS1, FGF9, ARPC1A, ITGA10, RAC1, PIP5K1A, MAPK3, PAK4, FGF2, CDC42, CHRM2, PAK6, FGF18, FGF5, ACTN4, ITGA7, PDGFC, MYLK3, PAK6, PDGFD, PIK3CA, LIMK1, FN1, FGF8, MAP2K1, ITGA4, FGF23, ARHGEF7, NCKAP1, FGFR2, ITGA6, VAV3, FGFR1, FGF16, MAPK1, FGF1, FGF7, DIAPH2, PIP4K2C, ENAH, ITGAL, EGF, MYLK, ITGA2B, RRAS, MYL5, PPP1CB, CHRM1, PDGFA, ITGB3, PIK3R2 | hsa-miR-3613-3p, hsa-miR-664b-3p, hsa-let-7f-1-3p, hsa-miR-6867-5p, hsa-miR-4663,  hsa-miR-29c-3p, hsa-miR-6837-5p, hsa-miR-152-3p, hsa-miR-1468-3p, hsa-miR-27a-3p,  hsa-miR-659-3p, hsa-miR-3613-5p, hsa-miR-5189-3p, hsa-miR-7-5p, hsa-miR-4635,  hsa-miR-629-3p, hsa-miR-371b-5p, hsa-miR-6744-5p, hsa-miR-8063, hsa-miR-548a-3p,  hsa-miR-548ac, hsa-miR-548x-3p, hsa-miR-16-2-3p, hsa-miR-195-3p, hsa-miR-338-5p,  hsa-miR-4668-5p, hsa-miR-3148, hsa-miR-34a-5p, hsa-miR-204-3p, hsa-miR-6785-5p,  hsa-miR-1208, hsa-miR-6893-5p, hsa-miR-330-5p, hsa-miR-6825-5p, hsa-miR-4270,  hsa-miR-101-5p, hsa-miR-635, hsa-miR-6774-5p, hsa-miR-8084, hsa-miR-7-1-3p,  hsa-miR-574-5p, hsa-miR-98-5p, hsa-miR-4439, hsa-miR-342-5p, hsa-miR-550a-3-5p,  hsa-miR-6830-5p, hsa-miR-3609, hsa-miR-4446-3p, hsa-miR-3175, hsa-miR-6886-5p,  hsa-miR-3128, hsa-miR-3689f, hsa-miR-885-3p, hsa-miR-4423-3p, hsa-miR-3194-3p,  hsa-miR-3201, hsa-miR-5100, hsa-miR-6507-3p, hsa-miR-6813-5p, hsa-miR-5088-5p,  hsa-miR-4448 |
| **PI3K-Akt signalling pathway** | IL2RG, LAMB2, TLR2, PHLPP2, PRLR, FGF12, GSK3B, PRKCA, RBL2, TSC1, PDGFRA, GNG13, NFKB1, PPP2R5E, MET, YWHAH, MYB, ITGB1, FIGF, SOS2, ITGB8, CDK4, LAMB1, ATF2, ITGA9, FGF14, GNG11, NRAS, FGFR3, PRKAA2, PPP2R3A, YWHAE, IFNA8, LPAR3, FGF13, THBS1, PPP2R2C, ITGA8, PIK3CB, THBS2, PPP2CA, CREB5, FGFR4, SYK, COL4A5, CDC37, COL24A1, PIK3R5, YWHAG, MCL1, CDK2, CCND2, COL27A1, PCK2, IFNB1, ITGB6, GNG12, ANGPT2, STK11, FGF10, IL7, ITGA5, RAF1, ITGA3, CHUK, LAMA1, PIK3AP1, BCL2, CDKN1B, PPP2R5D, YWHAB, MTCP1, EFNA3, PPP2R2B, IGF1R, EGFR, EFNA2, GNB1, FGF20, PPP2R2D, TLR4, PPP2R5C, COL6A6, ITGA1, KRAS, CDK6, RHEB, COL3A1, LPAR6, CREB3, FASLG, IL7R, EFNA5, IFNAR2, YWHAQ, RPS6KB2, PPP2R5A, LPAR4, GHR, EFNA4, IFNAR1, G6PC3, CREB1, IKBKB, COL6A1, FGF11, AKT2, PTK2, G6PC, BRCA1, ITGAV, ITGA11, CRTC2, PPP2R5B, COL2A1, IL4R, DDIT4, GNB2, TSC2, PIK3CD, PIK3R3, CCND1, JAK2, EIF4E, LPAR1, CCNE2, EPOR, COL4A2, AKT1, PGF, PPP2R2A, F2R, MYC, MLST8, RELN, COL5A1, PPP2R1A, PDGFB, COL1A1, GNG7, FLT1, EIF4B, KIT, VEGFC, GYS2, GNG2, COL4A3, ITGA2, PIK3R1, COL4A4, SOS1, JAK3, IL2RA, FGF9, HSP90B1, YWHAZ, ITGA10, PHLPP1, PPP2R3C, KITLG, COL11A2, IRS1, RAC1, INSR, MAPK3, PRKAA1, FGF2, CHRM2, CASP9, FGF18, NGF, FGF5, COL1A2, G6PC2, LAMC1, IGF1, ITGA7, BCL2L1, AKT3, PDGFC, COL11A1, THBS3, EIF4E2, CREB3L2, ANGPT1, FLT4, COL6A3, PDGFD, PIK3CA, CREB3L1, SGK2, COL4A6, OSM, FOXO3, FN1, FGF8, PKN2, CDKN1A, MAP2K1, TNR, GNG4, PDPK1, LAMA2, ITGA4, LAMC2, TCL1A, FGF23, HGF, MTOR, FGFR2, ITGA6, TNN, RELA, COL5A3, VEGFA, PTEN, FGFR1, SGK3, FGF16, OSMR, MAPK1, FGF1, PPP2R1B, FGF7, KDR, CSF1, GNG5, GRB2, COL5A2, GNB4, SPP1, TEK, CSF3, SGK1, JAK1, MDM2, NGFR, EGF, ITGA2B, GNB5, BCL2L11, CCND3, RPS6KB1, COL4A1, CHRM1, IL6R, PDGFA, LAMA4, EFNA1, ITGB3, PIK3R2, C8orf44-SGK3 | hsa-miR-3613-3p, hsa-miR-3148, hsa-miR-548x-3p, hsa-miR-574-5p, hsa-miR-371b-5p,  hsa-miR-338-5p, hsa-miR-8063, hsa-miR-27a-3p, hsa-miR-635, hsa-miR-6774-5p,  hsa-miR-548ac, hsa-miR-7-1-3p, hsa-miR-8084, hsa-miR-548a-3p, hsa-miR-664b-3p,  hsa-let-7f-1-3p, hsa-miR-195-3p, hsa-miR-6830-5p, hsa-miR-4668-5p, hsa-miR-659-3p,  hsa-miR-29c-3p, hsa-miR-4635, hsa-miR-6825-5p, hsa-miR-1468-3p, hsa-miR-1208,  hsa-miR-152-3p, hsa-miR-3609, hsa-miR-4270, hsa-miR-6893-5p, hsa-miR-6785-5p,  hsa-miR-6867-5p, hsa-miR-3194-3p, hsa-miR-34a-5p, hsa-miR-885-3p, hsa-miR-98-5p,  hsa-miR-330-5p, hsa-miR-4446-3p, hsa-miR-296-5p, hsa-miR-5088-5p, hsa-miR-629-3p,  hsa-miR-3689f, hsa-miR-4439, hsa-miR-6813-5p, hsa-miR-6837-5p, hsa-miR-7-5p,  hsa-miR-6744-5p, hsa-miR-5100, hsa-miR-5189-3p, hsa-miR-204-3p, hsa-miR-342-5p,  hsa-miR-16-2-3p, hsa-miR-335-5p, hsa-miR-550a-3-5p, hsa-miR-3613-5p, hsa-miR-4448,  hsa-miR-4720-5p, hsa-miR-4663, hsa-miR-3128, hsa-miR-6507-3p, hsa-miR-4462,  hsa-miR-3175, hsa-miR-6787-5p, hsa-miR-6886-5p, hsa-miR-3201, hsa-miR-4518 |
| **mTOR signalling pathway** | BRAF, PRKCA, TSC1, RRAGD, PRKAA2, PIK3CB, PIK3R5, STK11, RPS6KA1, RHEB, RPS6KB2, RICTOR, IKBKB, AKT2, AKT1S1, DDIT4, TSC2, PIK3CD, PIK3R3, EIF4E, HIF1A, AKT1, MLST8, EIF4B, PIK3R1, IRS1, MAPK3, PRKAA1, ULK3, PRKCB, RPS6KA3, IGF1, AKT3, EIF4E2, PIK3CA, PDPK1, MTOR, ULK1, TNF, VEGFA, PTEN, CAB39, MAPK1, RRAGC, ULK2, STRADA, CAB39L, RPS6KB1, RPS6KA2, PIK3R2 | hsa-miR-664b-3p, hsa-miR-1208, hsa-miR-7-5p, hsa-miR-8063, hsa-miR-8084,  hsa-miR-548ac, hsa-miR-548x-3p, hsa-miR-6886-5p, hsa-miR-3613-3p, hsa-miR-4668-5p, hsa-miR-6830-5p, hsa-miR-548a-3p, hsa-miR-27a-3p, hsa-miR-4439, hsa-miR-16-2-3p,  hsa-miR-195-3p, hsa-miR-7-1-3p, hsa-miR-338-5p, hsa-miR-152-3p, hsa-miR-6785-5p,  hsa-miR-98-5p, hsa-miR-6837-5p, hsa-miR-5100, hsa-miR-29c-3p, hsa-miR-4270,  hsa-miR-6825-5p, hsa-miR-635, hsa-miR-3194-3p, hsa-miR-6774-5p, hsa-miR-3148,  hsa-miR-6867-5p, hsa-miR-371b-5p, hsa-miR-342-5p, hsa-miR-330-5p, hsa-miR-4635,  hsa-miR-3613-5p, hsa-miR-550a-3-5p, hsa-miR-574-5p, hsa-miR-885-3p, hsa-let-7f-1-3p,  hsa-miR-1468-3p, hsa-miR-3689f, hsa-miR-4448, hsa-miR-34a-5p, hsa-miR-6893-5p,  hsa-miR-204-3p, hsa-miR-6813-5p, hsa-miR-659-3p, hsa-miR-6744-5p, hsa-miR-101-5p,  hsa-miR-3609 |
| **Estrogen signalling pathway** | ESR1, GABBR1, HSPA2, FOS, HBEGF, ADCY1, ADCY5, SOS2, ATF2, ADCY7, NRAS, ADCY2, CALM3, CALM1, GNAS, SHC1, PIK3CB, FKBP4, CREB5, HSPA1L, PIK3R5, RAF1, SHC2, EGFR, GNAI3, KRAS, SHC3, CREB3, CALM2, CREB1, GABBR2, AKT2, PLCB1, MMP2, ESR2, PIK3CD, PIK3R3, AKT1, GRM1, PRKACG, PRKACA, FKBP5, ITPR1, PIK3R1, SOS1, HSP90B1, PRKX, MAPK3, KCNJ6, SP1, SHC4, GNAQ, AKT3, CREB3L2, GNAI2, PIK3CA, CREB3L1, GNAO1, ADCY8, KCNJ9, MAP2K1, HSPA1B, ITPR3, MAPK1, ITPR2, GRB2, ADCY9, ADCY4, KCNJ5, KCNJ3, PLCB4, PLCB2, GNAI1, PRKACB, ADCY6, PIK3R2 | hsa-miR-3613-3p, hsa-miR-152-3p, hsa-miR-548a-3p, hsa-miR-6785-5p, hsa-miR-548ac,  hsa-miR-548x-3p, hsa-miR-27a-3p, hsa-miR-3148, hsa-miR-6830-5p, hsa-miR-664b-3p,  hsa-let-7f-1-3p, hsa-miR-4270, hsa-miR-16-2-3p, hsa-miR-195-3p, hsa-miR-4635,  hsa-miR-7-1-3p, hsa-miR-371b-5p, hsa-miR-338-5p, hsa-miR-8063, hsa-miR-6893-5p,  hsa-miR-5088-5p, hsa-miR-204-3p, hsa-miR-6813-5p, hsa-miR-330-5p, hsa-miR-1208,  hsa-miR-6825-5p, hsa-miR-1468-3p, hsa-miR-6507-3p, hsa-miR-635, hsa-miR-296-5p,  hsa-miR-6774-5p, hsa-miR-4446-3p, hsa-miR-3194-3p, hsa-miR-7-5p, hsa-miR-29c-3p,  hsa-miR-6744-5p, hsa-miR-342-5p, hsa-miR-34a-5p, hsa-miR-335-5p, hsa-miR-6867-5p,  hsa-miR-4668-5p, hsa-miR-3128, hsa-miR-6837-5p, hsa-miR-629-3p, hsa-miR-3689f,  hsa-miR-574-5p, hsa-miR-8084, hsa-miR-4423-3p, hsa-miR-6886-5p, hsa-miR-4663,  hsa-miR-98-5p, hsa-miR-659-3p, hsa-miR-3175, hsa-miR-550a-3-5p, hsa-miR-3613-5p,  hsa-miR-4720-5p, hsa-miR-885-3p, hsa-miR-4439 |
| **Chronic myeloid leukemia** | BRAF, E2F1, TGFBR1, NFKB1, SOS2, CDK4, CBL, E2F2, NRAS, CRKL, CRK, SHC1, RUNX1, PIK3CB, CTBP1, PIK3R5, BCR, CTBP2, RAF1, SMAD3, CHUK, CDKN1B, SHC2, KRAS, CDK6, STAT5B, SHC3, TGFB1, IKBKB, AKT2, CBLB, PIK3CD, PIK3R3, CCND1, SMAD4, E2F3, AKT1, MYC, NFKBIA, PIK3R1, RB1, SOS1, PTPN11, HDAC2, MAPK3, TGFB2, SHC4, BCL2L1, AKT3, PIK3CA, GAB2, MECOM, CDKN1A, MAP2K1, RELA, MAPK1, GRB2, TGFBR2, MDM2, ABL1, PIK3R2 | hsa-miR-330-5p, hsa-miR-1208, hsa-miR-7-5p, hsa-miR-4635, hsa-miR-4446-3p,  hsa-miR-5088-5p, hsa-miR-548a-3p, hsa-miR-548ac, hsa-miR-4439, hsa-miR-548x-3p,  hsa-miR-7-1-3p, hsa-miR-98-5p, hsa-miR-6785-5p, hsa-miR-6825-5p, hsa-miR-4270,  hsa-miR-371b-5p, hsa-miR-3128, hsa-miR-3613-3p, hsa-miR-3148, hsa-miR-152-3p,  hsa-miR-1468-3p, hsa-miR-8063, hsa-let-7f-1-3p, hsa-miR-3609, hsa-miR-34a-5p,  hsa-miR-664b-3p, hsa-miR-4668-5p, hsa-miR-6830-5p, hsa-miR-27a-3p, hsa-miR-16-2-3p, hsa-miR-195-3p, hsa-miR-338-5p, hsa-miR-3613-5p, hsa-miR-6893-5p, hsa-miR-6774-5p, hsa-miR-6507-3p, hsa-miR-8084, hsa-miR-6886-5p, hsa-miR-29c-3p, hsa-miR-6744-5p,  hsa-miR-885-3p, hsa-miR-629-3p, hsa-miR-4663, hsa-miR-6867-5p, hsa-miR-4462,  hsa-miR-204-3p, hsa-miR-3194-3p, hsa-miR-6837-5p, hsa-miR-659-3p, hsa-miR-635,  hsa-miR-342-5p, hsa-miR-4448, hsa-miR-4423-3p, hsa-miR-6813-5p |
| **Prostate cancer** | BRAF, GSK3B, PDGFRA, E2F1, ERBB2, NFKB1, SOS2, E2F2, NRAS, PIK3CB, CREB5, TCF7L2, TGFA, PIK3R5, CDK2, TCF7L1, RAF1, CHUK, BCL2, CDKN1B, INSRR, IGF1R, EGFR, KRAS, CREB3, CREB1, IKBKB, AKT2, AR, PIK3CD, PIK3R3, CCND1, CTNNB1, CCNE2, E2F3, AKT1, PDGFB, NFKBIA, PIK3R1, RB1, SOS1, HSP90B1, MAPK3, CASP9, IGF1, EP300, NKX3-1, AKT3, PDGFC, CREB3L2, PDGFD, PIK3CA, CREB3L1, LEF1, CDKN1A, MAP2K1, PDPK1, MTOR, FGFR2, RELA, TCF7, PTEN, FGFR1, FOXO1, MAPK1, CREBBP, GRB2, MDM2, EGF, PDGFA, PIK3R2 | hsa-miR-152-3p, hsa-miR-548a-3p, hsa-miR-548x-3p, hsa-miR-338-5p, hsa-miR-3613-3p,  hsa-miR-3148, hsa-miR-330-5p, hsa-miR-548ac, hsa-miR-27a-3p, hsa-miR-7-1-3p,  hsa-miR-6744-5p, hsa-miR-8063, hsa-miR-6785-5p, hsa-miR-371b-5p, hsa-miR-6893-5p,  hsa-miR-4668-5p, hsa-miR-29c-3p, hsa-miR-6867-5p, hsa-miR-4635, hsa-miR-629-3p,  hsa-miR-4270, hsa-miR-8084, hsa-miR-664b-3p, hsa-miR-4439, hsa-miR-7-5p,  hsa-let-7f-1-3p, hsa-miR-6837-5p, hsa-miR-6825-5p, hsa-miR-635, hsa-miR-3194-3p,  hsa-miR-6774-5p, hsa-miR-34a-5p, hsa-miR-4720-5p, hsa-miR-4518, hsa-miR-6830-5p,  hsa-miR-16-2-3p, hsa-miR-195-3p, hsa-miR-659-3p, hsa-miR-574-5p, hsa-miR-4446-3p,  hsa-miR-5088-5p, hsa-miR-342-5p, hsa-miR-98-5p, hsa-miR-1468-3p, hsa-miR-885-3p,  hsa-miR-6507-3p, hsa-miR-4663, hsa-miR-204-3p, hsa-miR-3128, hsa-miR-1208,  hsa-miR-3609, hsa-miR-5100, hsa-miR-6813-5p, hsa-miR-6886-5p, hsa-miR-3689f,  hsa-miR-550a-3-5p, hsa-miR-3613-5p |
| **Ubiquitin mediated proteolysis** | RNF7, UBE2Z, UBE2R2, UBE2Q2, UBE3B, UBE2E3, WWP2, CBL, BTRC, RFWD2, FBXW7, TRIM37, UBR5, WWP1, CUL2, SMURF2, NEDD4L, HUWE1, ITCH, UBE2E2, FBXO4, CUL1, NHLRC1, DET1, UBE4A, RHOBTB1, PRPF19, HERC3, MAP3K1, ANAPC1, UBE2J1, UBE2I, HERC1, TCEB1, MID1, PML, VHL, SKP1, CUL4A, CDC34, UBA2, TRIP12, SOCS3, UBA6, KLHL13, CBLB, BRCA1, PIAS3, CUL5, ANAPC4, ANAPC10, UBE2A, SKP2, SOCS1, TRAF6, UBE3A, HERC2, UBE3C, BIRC6, SMURF1, UBE2Q1, ANAPC7, UBE2N, RCHY1, UBE2K, SAE1, SIAH1, UBE2D4, MGRN1, CDC23, NEDD4, UBE2H, ERCC8, PIAS2, UBE2D3, HERC4, UBE2E1, UBE2G2, UBE2D1, CDC27, RBX1, UBE2M, KLHL9, UBE2D2, UBE2G1, BIRC3, CUL3, UBE2W, ANAPC13, UBE2B, UBA3, PPIL2, FBXW11, RHOBTB2, PARK2, UBE4B, TRIM32, PIAS1, UBE2C, MDM2, SYVN1, UBE2J2, XIAP, CUL4B, UBOX5 | hsa-miR-3613-3p, hsa-miR-1468-3p, hsa-miR-548ac, hsa-miR-664b-3p, hsa-miR-548x-3p, hsa-miR-7-1-3p, hsa-miR-3175, hsa-miR-659-3p, hsa-miR-3194-3p, hsa-miR-6744-5p,  hsa-miR-3148, hsa-let-7f-1-3p, hsa-miR-338-5p, hsa-miR-548a-3p, hsa-miR-3128,  hsa-miR-6785-5p, hsa-miR-34a-5p, hsa-miR-8063, hsa-miR-4446-3p, hsa-miR-330-5p,  hsa-miR-152-3p, hsa-miR-7-5p, hsa-miR-3613-5p, hsa-miR-8084, hsa-miR-1208,  hsa-miR-4635, hsa-miR-629-3p, hsa-miR-4668-5p, hsa-miR-16-2-3p, hsa-miR-27a-3p,  hsa-miR-5100, hsa-miR-6507-3p, hsa-miR-98-5p, hsa-miR-6830-5p, hsa-miR-4663,  hsa-miR-29c-3p, hsa-miR-101-5p, hsa-miR-4518, hsa-miR-371b-5p, hsa-miR-335-5p,  hsa-miR-4720-5p, hsa-miR-5189-3p, hsa-miR-4270, hsa-miR-6893-5p, hsa-miR-204-3p,  hsa-miR-6825-5p, hsa-miR-6867-5p, hsa-miR-5088-5p, hsa-miR-3609, hsa-miR-195-3p,  hsa-miR-3201, hsa-miR-4448, hsa-miR-296-5p, hsa-miR-3689f, hsa-miR-635,  hsa-miR-6774-5p, hsa-miR-6837-5p, hsa-miR-574-5p, hsa-miR-6787-5p, hsa-miR-885-3p |
| **Dorso-ventral axis formation** | PIWIL1, SPIRE1, SOS2, ETV6, PIWIL3, CPEB4, PIWIL4, ETS2, ETS1, PIWIL2, CPEB1, EGFR, NOTCH2, KRAS, NOTCH1, SOS1, CPEB2, MAPK3, NOTCH3, FMN2, MAP2K1, CPEB3, MAPK1, GRB2, SPIRE2 | hsa-miR-3613-3p, hsa-miR-3148, hsa-miR-152-3p, hsa-miR-548a-3p, hsa-miR-1468-3p,  hsa-miR-330-5p, hsa-miR-548ac, hsa-miR-548x-3p, hsa-miR-7-1-3p, hsa-miR-338-5p,  hsa-miR-8063, hsa-miR-6893-5p, hsa-miR-29c-3p, hsa-miR-664b-3p, hsa-miR-635,  hsa-miR-6774-5p, hsa-miR-4668-5p, hsa-miR-3609, hsa-miR-4270, hsa-miR-7-5p,  hsa-miR-98-5p, hsa-miR-204-3p, hsa-miR-6813-5p, hsa-miR-34a-5p, hsa-let-7f-1-3p,  hsa-miR-1208, hsa-miR-16-2-3p, hsa-miR-195-3p, hsa-miR-4635, hsa-miR-27a-3p,  hsa-miR-6744-5p, hsa-miR-8084, hsa-miR-4439, hsa-miR-6785-5p, hsa-miR-371b-5p,  hsa-miR-6507-3p, hsa-miR-101-5p |
| **Long-term depression** | BRAF, PRKCA, CACNA1A, GUCY1B3, NRAS, GNAS, GUCY1A3, PPP2CA, GNAZ, GNA13, PLA2G4F, RAF1, GRIA1, IGF1R, GNAI3, KRAS, GRIA2, NOS1, GNA11, RYR1, PLCB1, PRKG2, GRM1, PPP2R1A, ITPR1, MAPK3, PLA2G4D, GRID2, PRKCB, IGF1, GNAQ, GUCY1A2, GNAI2, GNAO1, MAP2K1, PRKG1, PLA2G4E, ITPR3, MAPK1, ITPR2, PPP2R1B, PLCB4, PLCB2, PLA2G4C, GNAI1, GRIA3 | hsa-miR-8084, hsa-miR-3148, hsa-miR-7-5p, hsa-miR-3613-3p, hsa-miR-4668-5p,  hsa-miR-6830-5p, hsa-miR-548a-3p, hsa-miR-548ac, hsa-miR-664b-3p, hsa-miR-27a-3p,  hsa-miR-4439, hsa-miR-548x-3p, hsa-miR-1208, hsa-miR-16-2-3p, hsa-miR-195-3p,  hsa-miR-7-1-3p, hsa-miR-338-5p, hsa-miR-6867-5p, hsa-miR-6785-5p, hsa-miR-3128,  hsa-miR-6837-5p, hsa-miR-574-5p, hsa-miR-8063, hsa-miR-98-5p, hsa-miR-885-3p,  hsa-let-7f-1-3p, hsa-miR-3613-5p, hsa-miR-5088-5p, hsa-miR-4635, hsa-miR-4270,  hsa-miR-6825-5p, hsa-miR-6893-5p, hsa-miR-1468-3p, hsa-miR-3175, hsa-miR-629-3p,  hsa-miR-335-5p, hsa-miR-3609, hsa-miR-659-3p, hsa-miR-6507-3p, hsa-miR-101-5p,  hsa-miR-371b-5p, hsa-miR-34a-5p, hsa-miR-3689f, hsa-miR-204-3p, hsa-miR-330-5p,  hsa-miR-152-3p, hsa-miR-29c-3p, hsa-miR-4448, hsa-miR-6813-5p, hsa-miR-6744-5p,  hsa-miR-3194-3p |
| **Prolactin signalling pathway** | ESR1, PRLR, FOS, GSK3B, STAT3, NFKB1, SOCS4, SOS2, NRAS, SOCS2, SHC1, PIK3CB, GALT, SLC2A2, PIK3R5, MAPK14, CCND2, RAF1, CYP17A1, TNFRSF11A, SHC2, KRAS, STAT5B, SHC3, TNFSF11, SOCS6, SOCS3, AKT2, MAPK9, ESR2, PIK3CD, PIK3R3, CCND1, JAK2, SOCS1, CISH, MAPK8, AKT1, SOCS5, PIK3R1, SOS1, MAPK3, SHC4, AKT3, PIK3CA, FOXO3, LHCGR, MAP2K1, STAT1, RELA, ELF5, MAPK1, GRB2, IRF1, MAPK10, PIK3R2 | hsa-miR-27a-3p, hsa-miR-548x-3p, hsa-miR-29c-3p, hsa-miR-6837-5p, hsa-miR-3613-3p,  hsa-miR-152-3p, hsa-miR-548a-3p, hsa-miR-6785-5p, hsa-miR-548ac, hsa-miR-4423-3p,  hsa-miR-6867-5p, hsa-miR-4439, hsa-miR-16-2-3p, hsa-miR-195-3p, hsa-miR-98-5p,  hsa-miR-330-5p, hsa-miR-664b-3p, hsa-miR-659-3p, hsa-miR-4270, hsa-miR-3148,  hsa-miR-7-1-3p, hsa-miR-6744-5p, hsa-miR-8063, hsa-miR-371b-5p, hsa-miR-342-5p,  hsa-miR-4668-5p, hsa-miR-6830-5p, hsa-miR-3609, hsa-let-7f-1-3p, hsa-miR-1468-3p,  hsa-miR-629-3p, hsa-miR-550a-3-5p, hsa-miR-4635, hsa-miR-204-3p, hsa-miR-6813-5p,  hsa-miR-1208, hsa-miR-4446-3p, hsa-miR-7-5p, hsa-miR-335-5p, hsa-miR-34a-5p,  hsa-miR-4518, hsa-miR-3194-3p, hsa-miR-3128, hsa-miR-5100, hsa-miR-338-5p,  hsa-miR-6893-5p, hsa-miR-4663, hsa-miR-4462, hsa-miR-3689f, hsa-miR-4720-5p,  hsa-miR-1260b, hsa-miR-8084, hsa-miR-6886-5p, hsa-miR-6507-3p, hsa-miR-885-3p |
| **Protein processing in endoplasmic reticulum** | UBQLNL, ATF6, HSPA2, SAR1A, DNAJA1, CAPN2, RPN1, PLAA, UBE2E3, SSR1, SAR1B, RAD23B, EIF2AK3, AMFR, DNAJC3, UGGT1, MBTPS1, ERO1L, UBQLN4, YOD1, NPLOC4, MAP2K7, SEL1L, UBQLN1, HSPA1L, DNAJB2, SEC23A, BAX, DNAJB11, DNAJC10, UBE2E2, HSPA5, BCL2, DNAJC5B, CUL1, MBTPS2, STT3A, DNAJC5, P4HB, XBP1, SEC61A1, UBE2J1, STT3B, HSPH1, PDIA6, CANX, SKP1, PREB, SEC61A2, EDEM1, SEC63, EDEM3, UBQLN2, MAN1A2, EIF2AK2, DERL2, MAPK9, MAN1C1, DNAJB1, BAG1, TUSC3, SEC23B, MAPK8, SEL1L2, RNF185, DERL3, PRKCSH, BLOC1S5-TXNDC5, VCP, UBQLN3, MOGS, SVIP, SEC62, SEC24A, ERP29, HSP90B1, PDIA3, ERN1, SEC31A, SEC61B, UBE2D4, HSPA4L, BAG2, EIF2S1, SSR3, NFE2L2, LMAN1, UBE2D3, UBE2E1, CKAP4, UGGT2, UBE2G2, SEC24B, UBE2D1, RBX1, NGLY1, UBE2D2, BAK1, UBE2G1, DNAJB12, RPN2, DERL1, CALR, ATXN3, VIMP, MAN1A1, HSPA1B, SEC31B, DNAJA2, MARCH6, TRAM1, NSFL1C, PARK2, UBE4B, ERO1LB, HYOU1, SYVN1, UBE2J2, SEC24D, MAP3K5, MAPK10, ERLEC1 | hsa-miR-4635, hsa-miR-3613-3p, hsa-miR-4668-5p, hsa-miR-3148, hsa-miR-204-3p,  hsa-miR-342-5p, hsa-miR-6830-5p, hsa-miR-548ac, hsa-miR-664b-3p, hsa-miR-27a-3p,  hsa-let-7f-1-3p, hsa-miR-548x-3p, hsa-miR-4270, hsa-miR-16-2-3p, hsa-miR-629-3p,  hsa-miR-7-1-3p, hsa-miR-338-5p, hsa-miR-8063, hsa-miR-5189-3p, hsa-miR-152-3p,  hsa-miR-34a-5p, hsa-miR-3128, hsa-miR-548a-3p, hsa-miR-1208, hsa-miR-330-5p,  hsa-miR-1468-3p, hsa-miR-98-5p, hsa-miR-371b-5p, hsa-miR-6837-5p, hsa-miR-6785-5p, hsa-miR-6825-5p, hsa-miR-29c-3p, hsa-miR-8084, hsa-miR-6893-5p, hsa-miR-550a-3-5p,  hsa-miR-101-5p, hsa-miR-1260b, hsa-miR-4720-5p, hsa-miR-6867-5p, hsa-miR-4439,  hsa-miR-6744-5p, hsa-miR-659-3p, hsa-miR-6787-5p, hsa-miR-4518, hsa-miR-574-5p,  hsa-miR-885-3p, hsa-miR-4785, hsa-miR-3194-3p, hsa-miR-4446-3p, hsa-miR-7-5p,  hsa-miR-4663, hsa-miR-335-5p, hsa-miR-3689f, hsa-miR-3609, hsa-miR-195-3p,  hsa-miR-3613-5p, hsa-miR-4448, hsa-miR-635, hsa-miR-6774-5p, hsa-miR-3175,  hsa-miR-5088-5p, hsa-miR-296-5p, hsa-miR-6813-5p, hsa-miR-4462, hsa-miR-5100,  hsa-miR-3201, hsa-miR-6507-3p |
| **Acute myeloid leukemia** | BRAF, PIM2, STAT3, NFKB1, SOS2, NRAS, RUNX1, PIK3CB, TCF7L2, ZBTB16, PIK3R5, TCF7L1, RAF1, CHUK, KRAS, STAT5B, PML, RPS6KB2, IKBKB, AKT2, RUNX1T1, PIK3CD, PIK3R3, CCND1, AKT1, CEBPA, MYC, PIM1, KIT, PIK3R1, SOS1, MAPK3, RARA, FLT3, AKT3, PIK3CA, LEF1, MAP2K1, MTOR, RELA, TCF7, MAPK1, GRB2, JUP, PPARD, RPS6KB1, PIK3R2 | hsa-miR-3613-3p, hsa-miR-34a-5p, hsa-miR-548ac, hsa-miR-195-3p, hsa-miR-3148,  hsa-miR-342-5p, hsa-miR-548a-3p, hsa-miR-664b-3p, hsa-miR-27a-3p, hsa-miR-548x-3p,  hsa-miR-371b-5p, hsa-miR-8063, hsa-miR-330-5p, hsa-miR-4635, hsa-miR-1208,  hsa-miR-7-5p, hsa-miR-4439, hsa-miR-7-1-3p, hsa-miR-4448, hsa-miR-6893-5p,  hsa-miR-4270, hsa-miR-8084, hsa-miR-6886-5p, hsa-miR-4668-5p, hsa-miR-6830-5p,  hsa-miR-16-2-3p, hsa-miR-338-5p, hsa-miR-1468-3p, hsa-miR-6744-5p, hsa-miR-152-3p,  hsa-miR-4446-3p, hsa-miR-6867-5p, hsa-miR-3609, hsa-miR-6507-3p, hsa-miR-29c-3p,  hsa-miR-98-5p, hsa-miR-6837-5p, hsa-miR-659-3p, hsa-miR-5100, hsa-miR-629-3p,  hsa-miR-6785-5p, hsa-miR-4720-5p, hsa-miR-4518, hsa-let-7f-1-3p, hsa-miR-885-3p,  hsa-miR-574-5p, hsa-miR-6825-5p, hsa-miR-635, hsa-miR-3194-3p, hsa-miR-6774-5p,  hsa-miR-204-3p, hsa-miR-6813-5p |
| **Prion diseases** | EGR1, BAX, HSPA5, SOD1, NCAM1, FYN, NCAM2, NOTCH1, PRKACG, PRKACA, PRNP, PRKX, MAPK3, C7, LAMC1, C6, MAP2K1, C8B, IL1A, MAPK1, ELK1, PRKACB | hsa-miR-5088-5p, hsa-miR-664b-3p, hsa-miR-548x-3p, hsa-miR-574-5p, hsa-miR-7-1-3p,  hsa-miR-8063, hsa-miR-4423-3p, hsa-miR-27a-3p, hsa-miR-6893-5p, hsa-miR-4270,  hsa-miR-3613-3p, hsa-miR-3128, hsa-miR-3148, hsa-miR-152-3p, hsa-miR-5100,  hsa-miR-3175, hsa-miR-6825-5p, hsa-miR-330-5p, hsa-miR-6785-5p, hsa-miR-548ac,  hsa-let-7f-1-3p, hsa-miR-550a-3-5p, hsa-miR-635, hsa-miR-7-5p, hsa-miR-16-2-3p,  hsa-miR-195-3p, hsa-miR-6774-5p, hsa-miR-338-5p, hsa-miR-4668-5p, hsa-miR-29c-3p,  hsa-miR-4439, hsa-miR-34a-5p, hsa-miR-1208, hsa-miR-204-3p, hsa-miR-6813-5p,  hsa-miR-8084, hsa-miR-1468-3p, hsa-miR-4635 |
| **Retrograde**  **endocannabinoid signalling** | GABRA1, PRKCA, CACNA1A, GNG13, ADCY1, ADCY5, GNG11, ADCY7, CACNA1F, ADCY2, GABRG2, GRM5, MAPK14, GNG12, SLC17A6, GRIA1, GNAI3, GNB1, MGLL, RIMS1, DAGLA, GRIA2, GABRB3, PLCB1, GABRA3, MAPK9, GNB2, CACNA1B, GABRA5, MAPK8, GRM1, PRKACG, PRKACA, GNG7, GABRG1, GNG2, PTGS2, ITPR1, SLC17A7, PRKX, CNR1, GABRA6, MAPK3, KCNJ6, PRKCB, GNAQ, GRIA4, GNAI2, GNAO1, ADCY8, KCNJ9, NAPEPLD, GABRA4, GNG4, GABRE, GABRB2, ITPR3, CACNA1D, ABHD6, MAPK1, ITPR2, GNG5, GABRP, ADCY9, ADCY4, GNB4, KCNJ5, KCNJ3, GABRA2, PLCB4, PLCB2, GNB5, MAPK10, GNAI1, PRKACB, GRIA3, ADCY6 | hsa-miR-27a-3p, hsa-miR-550a-3-5p, hsa-miR-3613-3p, hsa-miR-3148, hsa-miR-548a-3p,  hsa-miR-548ac, hsa-miR-548x-3p, hsa-miR-3194-3p, hsa-miR-4270, hsa-miR-16-2-3p,  hsa-miR-195-3p, hsa-miR-4635, hsa-miR-8063, hsa-miR-8084, hsa-miR-7-5p, hsa-miR-4448, hsa-miR-204-3p, hsa-miR-6813-5p, hsa-miR-330-5p, hsa-miR-34a-5p, hsa-miR-5088-5p,  hsa-miR-6893-5p, hsa-miR-3175, hsa-miR-6825-5p, hsa-miR-371b-5p, hsa-miR-6830-5p,  hsa-miR-664b-3p, hsa-miR-3613-5p, hsa-miR-7-1-3p, hsa-miR-1468-3p, hsa-let-7f-1-3p,  hsa-miR-1208, hsa-miR-4446-3p, hsa-miR-6867-5p, hsa-miR-3689f, hsa-miR-6744-5p,  hsa-miR-98-5p, hsa-miR-6787-5p, hsa-miR-6837-5p, hsa-miR-6785-5p, hsa-miR-4663,  hsa-miR-338-5p, hsa-miR-101-5p, hsa-miR-29c-3p, hsa-miR-4423-3p, hsa-miR-4668-5p,  hsa-miR-152-3p, hsa-miR-635, hsa-miR-659-3p, hsa-miR-574-5p, hsa-miR-296-5p,  hsa-miR-6774-5p, hsa-miR-3128, hsa-miR-629-3p, hsa-miR-4518, hsa-miR-342-5p |
| **Colorectal cancer** | BRAF, FOS, GSK3B, TGFBR1, SMAD2, APC, PIK3CB, MSH3, TCF7L2, PIK3R5, BAX, TCF7L1, RAF1, SMAD3, BCL2, BIRC5, RHOA, APPL1, KRAS, TGFB1, AKT2, MAPK9, DCC, CASP3, PIK3CD, PIK3R3, CCND1, SMAD4, CTNNB1, MSH6, AXIN2, MAPK8, AKT1, MYC, PIK3R1, RAC1, MAPK3, CASP9, TGFB2, AKT3, PIK3CA, RALGDS, LEF1, MAP2K1, CYCS, TCF7, MAPK1, TGFBR2, MAPK10, PIK3R2 | hsa-miR-3613-3p, hsa-miR-4668-5p, hsa-miR-3148, hsa-miR-1468-3p, hsa-miR-548ac,  hsa-miR-548x-3p, hsa-miR-3689f, hsa-miR-4635, hsa-miR-7-1-3p, hsa-miR-6744-5p,  hsa-miR-8063, hsa-miR-659-3p, hsa-miR-152-3p, hsa-miR-664b-3p, hsa-miR-27a-3p,  hsa-miR-3613-5p, hsa-miR-5189-3p, hsa-miR-7-5p, hsa-miR-629-3p, hsa-miR-371b-5p,  hsa-miR-6830-5p, hsa-miR-548a-3p, hsa-miR-3609, hsa-miR-6785-5p, hsa-let-7f-1-3p,  hsa-miR-34a-5p, hsa-miR-3194-3p, hsa-miR-330-5p, hsa-miR-4462, hsa-miR-98-5p,  hsa-miR-204-3p, hsa-miR-338-5p, hsa-miR-4720-5p, hsa-miR-4518, hsa-miR-29c-3p,  hsa-miR-6837-5p, hsa-miR-1208, hsa-miR-4663, hsa-miR-6893-5p, hsa-miR-3128,  hsa-miR-4270, hsa-miR-342-5p, hsa-miR-6813-5p, hsa-miR-885-3p, hsa-miR-6825-5p,  hsa-miR-4446-3p, hsa-miR-635, hsa-miR-6774-5p, hsa-miR-4448, hsa-miR-8084,  hsa-miR-6886-5p, hsa-miR-550a-3-5p, hsa-miR-6867-5p, hsa-miR-4439, hsa-miR-16-2-3p, hsa-miR-195-3p |
| **Choline metabolism in cancer** | SLC44A2, FOS, PRKCA, TSC1, PDGFRA, WASF1, SLC44A5, WASL, SOS2, NRAS, DGKE, PIK3CB, PIK3R5, PCYT1A, SLC22A3, PLA2G4F, RAF1, PLD1, EGFR, CHPT1, WASF3, DGKG, KRAS, RHEB, SLC22A5, PIP5K1B, RPS6KB2, AKT2, MAPK9, PLCG1, TSC2, PIK3CD, DGKZ, PIK3R3, HIF1A, MAPK8, AKT1, PPAP2B, PDGFB, WASF2, PIK3R1, SOS1, SLC22A2, RAC1, PIP5K1A, MAPK3, PLA2G4D, SP1, PRKCB, DGKB, SLC44A1, GPCPD1, AKT3, PDGFC, PDGFD, PIK3CA, RALGDS, MAP2K1, PCYT1B, PDPK1, DGKD, MTOR, PLA2G4E, CHKA, MAPK1, GRB2, DGKQ, DGKI, LYPLA1, PPAP2A, EGF, PLA2G4C, SLC44A3, MAPK10, RPS6KB1, DGKH, SLC5A7, PDGFA, PIK3R2 | hsa-miR-3613-3p, hsa-miR-3148, hsa-miR-34a-5p, hsa-miR-3613-5p, hsa-miR-4635,  hsa-miR-4448, hsa-miR-4270, hsa-miR-152-3p, hsa-miR-548a-3p, hsa-miR-6785-5p,  hsa-miR-548ac, hsa-miR-548x-3p, hsa-miR-1208, hsa-miR-338-5p, hsa-miR-6744-5p,  hsa-miR-98-5p, hsa-miR-6813-5p, hsa-let-7f-1-3p, hsa-miR-7-1-3p, hsa-miR-8063,  hsa-miR-664b-3p, hsa-miR-29c-3p, hsa-miR-6837-5p, hsa-miR-8084, hsa-miR-27a-3p,  hsa-miR-7-5p, hsa-miR-659-3p, hsa-miR-3175, hsa-miR-6825-5p, hsa-miR-5088-5p,  hsa-miR-6893-5p, hsa-miR-3128, hsa-miR-1468-3p, hsa-miR-330-5p, hsa-miR-6867-5p,  hsa-miR-16-2-3p, hsa-miR-371b-5p, hsa-miR-635, hsa-miR-3194-3p, hsa-miR-6774-5p,  hsa-miR-342-5p, hsa-miR-550a-3-5p, hsa-miR-574-5p, hsa-miR-4668-5p, hsa-miR-6830-5p, hsa-miR-4439, hsa-miR-5100, hsa-miR-6507-3p, hsa-miR-101-5p, hsa-miR-885-3p,  hsa-miR-204-3p, hsa-miR-3609, hsa-miR-1260b, hsa-miR-3689f, hsa-miR-195-3p,  hsa-miR-629-3p, hsa-miR-4423-3p, hsa-miR-4446-3p, hsa-miR-4720-5p, hsa-miR-6886-5p |
| **Dilated cardiomyopathy** | ACTB, CACNG8, DES, CACNA2D3, ADCY1, ADCY5, ITGB1, ITGB8, TPM1, ITGA9, ADCY7, CACNA1F, ADCY2, GNAS, ITGA8, CACNB4, CACNG7, ACTG1, TTN, ITGB6, SGCD, ITGA5, ITGA3, ITGA1, TGFB1, TPM3, TPM4, ITGAV, ITGA11, DMD, ACTC1, RYR2, PLN, PRKACG, PRKACA, CACNB1, SLC8A1, DAG1, CACNG4, ITGA2, ITGA10, PRKX, CACNA2D4, CACNA2D2, CACNG2, SGCA, IGF1, TGFB2, ITGA7, ADCY8, LMNA, LAMA2, ITGA4, CACNG6, CACNA2D1, ATP2A2, CACNB2, ITGA6, TNF, CACNA1D, ADCY9, ADCY4, SGCB, ADRB1, ITGA2B, TPM2, PRKACB, CACNB3, ADCY6, ITGB3 | hsa-miR-3613-3p, hsa-miR-98-5p, hsa-miR-6837-5p, hsa-miR-548ac, hsa-miR-4270,  hsa-miR-635, hsa-miR-296-5p, hsa-miR-6774-5p, hsa-miR-8063, hsa-miR-5088-5p,  hsa-miR-4635, hsa-miR-6893-5p, hsa-miR-548x-3p, hsa-miR-6785-5p, hsa-miR-3148,  hsa-miR-548a-3p, hsa-miR-3613-5p, hsa-miR-3194-3p, hsa-miR-338-5p, hsa-miR-4668-5p, hsa-let-7f-1-3p, hsa-miR-1208, hsa-miR-8084, hsa-miR-664b-3p, hsa-miR-7-1-3p,  hsa-miR-574-5p, hsa-miR-4423-3p, hsa-miR-27a-3p, hsa-miR-659-3p, hsa-miR-1468-3p,  hsa-miR-6744-5p, hsa-miR-629-3p, hsa-miR-204-3p, hsa-miR-885-3p, hsa-miR-330-5p,  hsa-miR-34a-5p, hsa-miR-6825-5p, hsa-miR-7-5p, hsa-miR-16-2-3p, hsa-miR-371b-5p,  hsa-miR-152-3p, hsa-miR-29c-3p, hsa-miR-101-5p, hsa-miR-6867-5p, hsa-miR-195-3p,  hsa-miR-4663, hsa-miR-3175, hsa-miR-342-5p, hsa-miR-3128, hsa-miR-4446-3p,  hsa-miR-550a-3-5p, hsa-miR-3201 |
| **GABAergic synapse** | GABBR1, SLC38A1, GABRA1, PLCL1, PRKCA, CACNA1A, GNG13, ADCY1, ADCY5, GPHN, GNG11, ADCY7, CACNA1F, ADCY2, GABARAPL2, HAP1, GABRG2, GNG12, GNAI3, GLS, GNB1, GABBR2, GABRB3, GABRA3, TRAK2, GNB2, CACNA1B, GABRA5, SLC6A11, PRKACG, PRKACA, GNG7, SLC38A2, GABRG1, GLUL, GNG2, GAD2, PRKX, GABRA6, KCNJ6, PRKCB, GNAI2, ABAT, GNAO1, NSF, ADCY8, GABRA4, GNG4, GABRE, SLC12A5, GABRB2, CACNA1D, GNG5, GLS2, GABRP, ADCY9, ADCY4, GNB4, GABRA2, SLC6A1, GNB5, GNAI1, PRKACB, ADCY6 | hsa-miR-3613-3p, hsa-miR-3148, hsa-miR-342-5p, hsa-miR-635, hsa-miR-296-5p,  hsa-miR-6774-5p, hsa-miR-8063, hsa-miR-548a-3p, hsa-miR-27a-3p, hsa-miR-371b-5p,  hsa-miR-548x-3p, hsa-miR-7-1-3p, hsa-miR-6893-5p, hsa-miR-4668-5p, hsa-miR-204-3p,  hsa-miR-6785-5p, hsa-miR-664b-3p, hsa-miR-152-3p, hsa-miR-550a-3-5p, hsa-miR-629-3p,  hsa-miR-4448, hsa-miR-574-5p, hsa-miR-548ac, hsa-miR-4423-3p, hsa-miR-1468-3p,  hsa-miR-6867-5p, hsa-miR-101-5p, hsa-miR-330-5p, hsa-miR-4270, hsa-miR-4635,  hsa-miR-98-5p, hsa-miR-29c-3p, hsa-miR-3175, hsa-miR-6825-5p, hsa-let-7f-1-3p,  hsa-miR-5100, hsa-miR-1208, hsa-miR-6787-5p, hsa-miR-3128, hsa-miR-7-5p,  hsa-miR-16-2-3p, hsa-miR-4518, hsa-miR-34a-5p, hsa-miR-338-5p, hsa-miR-3613-5p,  hsa-miR-3194-3p, hsa-miR-4446-3p, hsa-miR-659-3p, hsa-miR-195-3p, hsa-miR-6830-5p,  hsa-miR-6744-5p, hsa-miR-8084, hsa-miR-885-3p, hsa-miR-5189-3p |
| **Other types of O-glycan biosynthesis** | ST3GAL3, FUT4, OGT, POMT2, POFUT2, MFNG, ST6GAL2, GXYLT1, POGLUT1, B3GAT2, B4GALT1, ST6GAL1, B3GAT1, GXYLT2, POMGNT1, POMT1, COLGALT2, EOGT, FUT9, B4GALT3, B3GALTL, POFUT1, MGAT5B, COLGALT1 | hsa-miR-34a-5p, hsa-miR-3613-3p, hsa-miR-548a-3p, hsa-miR-548ac, hsa-miR-664b-3p,  hsa-miR-548x-3p, hsa-miR-574-5p, hsa-miR-371b-5p, hsa-miR-8063, hsa-miR-98-5p,  hsa-miR-152-3p, hsa-miR-4270, hsa-miR-7-5p, hsa-miR-4635, hsa-miR-338-5p,  hsa-miR-3148, hsa-miR-4462, hsa-miR-6744-5p, hsa-miR-204-3p, hsa-miR-6837-5p,  hsa-miR-8084, hsa-miR-27a-3p, hsa-let-7f-1-3p, hsa-miR-4439, hsa-miR-4663,  hsa-miR-7-1-3p, hsa-miR-4448, hsa-miR-1468-3p, hsa-miR-6830-5p, hsa-miR-4446-3p,  hsa-miR-29c-3p, hsa-miR-330-5p, hsa-miR-6785-5p, hsa-miR-6825-5p, hsa-miR-4668-5p, hsa-miR-1208 |
| **T cell receptor signalling pathway** | FOS, GSK3B, PRKCQ, NFKB1, SOS2, CDK4, CBL, NRAS, PIK3CB, PAK2, MAP2K7, TEC, DLG1, PIK3R5, MAPK14, PAK7, PPP3R1, CD40LG, RAF1, CHUK, BCL10, CD3D, RHOA, KRAS, PAK3, FYN, PAK1, VAV2, IKBKB, NCK1, PTPRC, PPP3CA, AKT2, CBLB, CD28, MAPK9, PLCG1, PIK3CD, PIK3R3, PTPN6, PPP3CB, RASGRP1, NCK2, NFATC2, AKT1, ICOS, IL5, NFKBIA, PIK3R1, SOS1, MAPK3, PAK4, CDC42, PAK6, CD247, AKT3, PAK6, CD4, PIK3CA, CD3G, MALT1, IFNG, GRAP2, MAP2K1, MAP3K8, PDPK1, CTLA4, TNF, RELA, LCP2, VAV3, NFATC3, CD8A, MAPK1, MAP3K7, NFATC1, IL10, GRB2, PDCD1, PPP3R2, PIK3R2 | hsa-miR-3128, hsa-miR-8063, hsa-miR-1468-3p, hsa-miR-29c-3p, hsa-miR-6867-5p,  hsa-miR-4446-3p, hsa-miR-7-5p, hsa-miR-3148, hsa-miR-548ac, hsa-miR-664b-3p,  hsa-miR-3613-3p, hsa-miR-330-5p, hsa-let-7f-1-3p, hsa-miR-548x-3p, hsa-miR-635,  hsa-miR-7-1-3p, hsa-miR-338-5p, hsa-miR-34a-5p, hsa-miR-574-5p, hsa-miR-4635,  hsa-miR-6837-5p, hsa-miR-6785-5p, hsa-miR-27a-3p, hsa-miR-371b-5p, hsa-miR-6825-5p, hsa-miR-98-5p, hsa-miR-548a-3p, hsa-miR-3194-3p, hsa-miR-6744-5p, hsa-miR-4668-5p, hsa-miR-6830-5p, hsa-miR-3609, hsa-miR-152-3p, hsa-miR-6507-3p, hsa-miR-1208,  hsa-miR-204-3p, hsa-miR-6813-5p, hsa-miR-4663, hsa-miR-8084, hsa-miR-6886-5p,  hsa-miR-550a-3-5p, hsa-miR-4270, hsa-miR-342-5p, hsa-miR-16-2-3p, hsa-miR-195-3p,  hsa-miR-659-3p, hsa-miR-6893-5p, hsa-miR-885-3p, hsa-miR-5189-3p, hsa-miR-4439,  hsa-miR-629-3p, hsa-miR-6774-5p, hsa-miR-5088-5p |
| **Gastric acid secretion** | CAMK2D, ACTB, PRKCA, MYLK4, ATP1B2, ADCY1, ADCY5, EZR, ADCY7, ADCY2, CALM3, CALM1, GNAS, ATP1A2, CA2, ACTG1, KCNJ16, CCKBR, KCNK2, CHRM3, ATP1B1, MYLK2, GNAI3, KCNJ1, CALM2, SLC26A7, CAMK2A, PLCB1, SLC9A1, ATP1B3, SSTR2, PRKACG, PRKACA, KCNJ10, ITPR1, PRKX, ATP1B4, PRKCB, KCNJ2, GNAQ, MYLK3, CAMK2B, GNAI2, ADCY8, ATP4B, SLC9A4, ATP1A1, ITPR3, ITPR2, ADCY9, ADCY4, KCNQ1, HRH2, PLCB4, PLCB2, MYLK, GNAI1, KCNJ15, PRKACB, ADCY6 | hsa-miR-152-3p, hsa-miR-8063, hsa-miR-6825-5p, hsa-miR-3148, hsa-miR-27a-3p,  hsa-miR-3613-3p, hsa-miR-8084, hsa-miR-4668-5p, hsa-miR-204-3p, hsa-miR-548a-3p,  hsa-miR-1468-3p, hsa-miR-34a-5p, hsa-miR-548ac, hsa-miR-664b-3p, hsa-let-7f-1-3p,  hsa-miR-548x-3p, hsa-miR-338-5p, hsa-miR-3128, hsa-miR-3175, hsa-miR-4720-5p,  hsa-miR-7-1-3p, hsa-miR-5088-5p, hsa-miR-3613-5p, hsa-miR-4663, hsa-miR-335-5p,  hsa-miR-6867-5p, hsa-miR-574-5p, hsa-miR-4423-3p, hsa-miR-6893-5p, hsa-miR-1208,  hsa-miR-7-5p, hsa-miR-4635, hsa-miR-629-3p, hsa-miR-4446-3p, hsa-miR-550a-3-5p,  hsa-miR-3194-3p, hsa-miR-6744-5p, hsa-miR-6785-5p, hsa-miR-330-5p, hsa-miR-371b-5p, hsa-miR-342-5p, hsa-miR-6830-5p, hsa-miR-6813-5p, hsa-miR-6837-5p, hsa-miR-4270,  hsa-miR-29c-3p, hsa-miR-98-5p, hsa-miR-635, hsa-miR-296-5p, hsa-miR-6774-5p,  hsa-miR-4439, hsa-miR-3689f, hsa-miR-659-3p, hsa-miR-101-5p |
| **Endometrial cancer** | BRAF, GSK3B, ERBB2, SOS2, NRAS, APC, PIK3CB, TCF7L2, PIK3R5, TCF7L1, RAF1, EGFR, KRAS, AKT2, CDH1, PIK3CD, PIK3R3, CCND1, CTNNB1, CTNNA1, AXIN2, AKT1, MYC, PIK3R1, SOS1, MAPK3, CASP9, AKT3, CTNNA3, PIK3CA, LEF1, FOXO3, MAP2K1, PDPK1, TCF7, CTNNA2, PTEN, MAPK1, GRB2, ELK1, EGF, ILK, PIK3R2 | hsa-miR-664b-3p, hsa-miR-4439, hsa-miR-6774-5p, hsa-miR-8063, hsa-miR-330-5p,  hsa-miR-4635, hsa-miR-3613-3p, hsa-miR-8084, hsa-miR-4668-5p, hsa-miR-6830-5p,  hsa-miR-548a-3p, hsa-miR-5100, hsa-miR-548x-3p, hsa-miR-7-1-3p, hsa-miR-371b-5p,  hsa-miR-6893-5p, hsa-miR-3148, hsa-miR-3609, hsa-miR-6785-5p, hsa-miR-548ac,  hsa-miR-27a-3p, hsa-let-7f-1-3p, hsa-miR-34a-5p, hsa-miR-1208, hsa-miR-885-3p,  hsa-miR-6825-5p, hsa-miR-16-2-3p, hsa-miR-195-3p, hsa-miR-338-5p, hsa-miR-204-3p,  hsa-miR-6813-5p, hsa-miR-6837-5p, hsa-miR-4446-3p, hsa-miR-4270, hsa-miR-3175,  hsa-miR-7-5p, hsa-miR-29c-3p, hsa-miR-4663, hsa-miR-4462, hsa-miR-152-3p,  hsa-miR-1468-3p, hsa-miR-6886-5p, hsa-miR-5189-3p, hsa-miR-3194-3p, hsa-miR-6744-5p, hsa-miR-6867-5p, hsa-miR-6507-3p, hsa-miR-4720-5p, hsa-miR-4518, hsa-miR-98-5p,  hsa-miR-659-3p, hsa-miR-635, hsa-miR-296-5p, hsa-miR-342-5p, hsa-miR-3613-5p,  hsa-miR-629-3p |
| **Circadian entrainment** | CAMK2D, FOS, PRKCA, GUCY1B3, GNG13, CACNA1G, ADCY1, ADCY5, GNG11, ADCY7, ADCY2, CALM3, CALM1, GNAS, GUCY1A3, NOS1AP, GNG12, ADCY10, PER3, MTNR1B, GRIA1, GNAI3, GNB1, CALM2, RPS6KA5, CACNA1I, CREB1, GRIA2, CAMK2A, NOS1, RYR1, PLCB1, PRKG2, GNB2, RYR2, PRKACG, PRKACA, GNG7, GNG2, ITPR1, PRKX, MAPK3, RYR3, KCNJ6, PRKCB, GNAQ, CAMK2B, GUCY1A2, GRIA4, GNAI2, GRIN2D, GNAO1, ADCY8, KCNJ9, GNG4, PER2, PRKG1, GRIN1, ITPR3, CACNA1D, ADCYAP1R1, MAPK1, GNG5, GRIN2A, ADCY9, ADCY4, GNB4, KCNJ5, KCNJ3, PLCB4, PLCB2, GNB5, GNAI1, PRKACB, GRIA3, ADCY6, GRIN2B | hsa-miR-3613-3p, hsa-miR-4423-3p, hsa-miR-4668-5p, hsa-miR-3148, hsa-miR-152-3p,  hsa-miR-29c-3p, hsa-miR-548a-3p, hsa-miR-1468-3p, hsa-miR-330-5p, hsa-miR-6785-5p, hsa-miR-548ac, hsa-miR-664b-3p, hsa-miR-27a-3p, hsa-miR-635, hsa-miR-659-3p,  hsa-miR-548x-3p, hsa-miR-574-5p, hsa-miR-4635, hsa-miR-7-1-3p, hsa-miR-371b-5p,  hsa-miR-338-5p, hsa-miR-8063, hsa-let-7f-1-3p, hsa-miR-6825-5p, hsa-miR-3194-3p,  hsa-miR-204-3p, hsa-miR-6813-5p, hsa-miR-1208, hsa-miR-16-2-3p, hsa-miR-195-3p,  hsa-miR-3201, hsa-miR-8084, hsa-miR-4446-3p, hsa-miR-4270, hsa-miR-5189-3p,  hsa-miR-7-5p, hsa-miR-629-3p, hsa-miR-34a-5p, hsa-miR-5088-5p, hsa-miR-6893-5p,  hsa-miR-6744-5p, hsa-miR-3605-3p, hsa-miR-3689f, hsa-miR-3128, hsa-miR-6837-5p,  hsa-miR-6774-5p, hsa-miR-335-5p, hsa-miR-6867-5p, hsa-miR-6830-5p, hsa-miR-342-5p, hsa-miR-4448, hsa-miR-3175, hsa-miR-550a-3-5p, hsa-miR-4720-5p, hsa-miR-98-5p,  hsa-miR-3613-5p, hsa-miR-4663, hsa-miR-6787-5p, hsa-miR-3609, hsa-miR-6507-3p,  hsa-miR-101-5p, hsa-miR-296-5p |
| **Amoebiasis** | LAMB2, TLR2, PRKCA, ARG2, ACTN2, NFKB1, ADCY1, ARG1, SERPINB4, SERPINB13, IL1R1, LAMB1, CXCL8, GNAS, PIK3CB, COL4A5, COL24A1, PIK3R5, RAB7A, COL27A1, SERPINB9, LAMA1, VCL, TLR4, COL3A1, TGFB1, SERPINB10, HSPB1, RAB5A, PTK2, GNA11, PLCB1, COL2A1, CASP3, PIK3CD, PIK3R3, COL4A2, ITGAM, COL5A1, PRKACG, COL1A1, PRKACA, COL4A3, SERPINB3, PIK3R1, COL4A4, PRKX, COL11A2, NOS2, SERPINB1, COL1A2, ACTN4, PRKCB, LAMC1, TGFB2, GNAL, GNAQ, COL11A1, PIK3CA, COL4A6, IFNG, FN1, LAMA2, LAMC2, C8B, RAB5C, TNF, RELA, COL5A3, IL10, COL5A2, RAB5B, PLCB4, PLCB2, PRKACB, COL4A1, LAMA4, PIK3R2 | hsa-miR-3128, hsa-miR-3613-3p, hsa-miR-3148, hsa-miR-152-3p, hsa-miR-548ac,  hsa-miR-548x-3p, hsa-miR-1208, hsa-miR-29c-3p, hsa-miR-6825-5p, hsa-miR-98-5p,  hsa-miR-4448, hsa-miR-204-3p, hsa-miR-6785-5p, hsa-miR-4720-5p, hsa-miR-27a-3p,  hsa-miR-296-5p, hsa-miR-629-3p, hsa-miR-3609, hsa-miR-659-3p, hsa-miR-5088-5p,  hsa-miR-342-5p, hsa-miR-548a-3p, hsa-miR-664b-3p, hsa-miR-371b-5p, hsa-miR-8063,  hsa-miR-6830-5p, hsa-miR-8084, hsa-miR-7-5p, hsa-miR-3175, hsa-miR-330-5p,  hsa-miR-4635, hsa-miR-34a-5p, hsa-miR-3689f, hsa-miR-7-1-3p, hsa-miR-4446-3p,  hsa-miR-550a-3-5p, hsa-miR-3194-3p, hsa-miR-6893-5p, hsa-miR-3613-5p, hsa-miR-1468-3p, hsa-miR-6867-5p, hsa-miR-4668-5p, hsa-let-7f-1-3p, hsa-miR-16-2-3p, hsa-miR-195-3p,  hsa-miR-6744-5p, hsa-miR-335-5p, hsa-miR-4270, hsa-miR-338-5p, hsa-miR-635,  hsa-miR-6774-5p, hsa-miR-6837-5p, hsa-miR-4439, hsa-miR-574-5p, hsa-miR-4423-3p |
| **p53 signalling pathway** | CCNG1, ZMAT3, CDK4, BID, BAI1, RFWD2, THBS1, RPRM, CDK2, CCND2, BAX, PERP, RRM2B, CDK1, GADD45A, CDK6, CHEK1, APAF1, ATM, PMAIP1, CD82, CASP3, TSC2, CCND1, CCNE2, TP73, SHISA5, EI24, RCHY1, SESN1, SIAH1, CASP9, SESN2, TNFRSF10B, MDM4, FAS, IGF1, BBC3, CASP8, SERPINB5, IGFBP3, CDKN1A, TP53AIP1, CHEK2, RRM2, SESN3, CYCS, PTEN, SERPINE1, CCNG2, PIDD1, PPM1D, MDM2, CCND3, ATR | hsa-miR-8084, hsa-miR-4668-5p, hsa-miR-659-3p, hsa-miR-4270, hsa-miR-3613-3p,  hsa-miR-548ac, hsa-miR-664b-3p, hsa-miR-6825-5p, hsa-miR-548x-3p, hsa-miR-8063,  hsa-miR-6785-5p, hsa-miR-4635, hsa-miR-371b-5p, hsa-miR-3148, hsa-miR-152-3p,  hsa-miR-29c-3p, hsa-miR-548a-3p, hsa-miR-6867-5p, hsa-miR-16-2-3p, hsa-miR-195-3p,  hsa-miR-7-1-3p, hsa-miR-98-5p, hsa-miR-1468-3p, hsa-miR-204-3p, hsa-miR-4663,  hsa-miR-6830-5p, hsa-miR-27a-3p, hsa-miR-550a-3-5p, hsa-miR-3689f, hsa-miR-338-5p,  hsa-miR-6893-5p, hsa-miR-330-5p, hsa-miR-4439, hsa-miR-574-5p, hsa-miR-885-3p,  hsa-miR-34a-5p, hsa-miR-4423-3p, hsa-miR-6774-5p, hsa-miR-6837-5p, hsa-let-7f-1-3p,  hsa-miR-3613-5p, hsa-miR-629-3p, hsa-miR-4448, hsa-miR-635, hsa-miR-3609,  hsa-miR-3175, hsa-miR-6507-3p, hsa-miR-4518, hsa-miR-296-5p, hsa-miR-5189-3p,  hsa-miR-7-5p |
| **Chagas disease (American trypanosomiasis)** | CCL2, TLR2, FOS, TGFBR1, NFKB1, ADCY1, CXCL8, SMAD2, GNAS, PPP2R2C, PIK3CB, PPP2CA, IFNGR2, PIK3R5, MAPK14, IFNB1, BDKRB2, SMAD3, CHUK, CD3D, PPP2R2B, ACE, GNAI3, PPP2R2D, TLR4, IFNGR1, TGFB1, FASLG, FADD, IKBKB, AKT2, GNA11, PLCB1, MYD88, MAPK9, PIK3CD, PIK3R3, MAPK8, TRAF6, AKT1, PPP2R2A, PPP2R1A, NFKBIA, PIK3R1, CFLAR, NOS2, MAPK3, FAS, TGFB2, GNAL, CD247, CASP8, GNAQ, AKT3, GNAI2, PIK3CA, CD3G, GNAO1, TNFRSF1A, IFNG, CALR, TLR9, MAP2K4, TNF, RELA, IRAK1, IRAK4, MAPK1, PPP2R1B, IL10, SERPINE1, TGFBR2, PLCB4, PLCB2, MAPK10, GNAI1, PIK3R2 | hsa-miR-3148, hsa-miR-548a-3p, hsa-miR-548x-3p, hsa-miR-7-1-3p, hsa-miR-6893-5p,  hsa-miR-27a-3p, hsa-miR-3175, hsa-miR-6825-5p, hsa-miR-629-3p, hsa-miR-6785-5p,  hsa-miR-7-5p, hsa-miR-4663, hsa-miR-4448, hsa-miR-330-5p, hsa-miR-3194-3p,  hsa-miR-3613-3p, hsa-miR-4446-3p, hsa-miR-6813-5p, hsa-let-7f-1-3p, hsa-miR-8084,  hsa-miR-4668-5p, hsa-miR-204-3p, hsa-miR-1468-3p, hsa-miR-34a-5p, hsa-miR-548ac,  hsa-miR-664b-3p, hsa-miR-338-5p, hsa-miR-6867-5p, hsa-miR-1208, hsa-miR-6886-5p,  hsa-miR-29c-3p, hsa-miR-8063, hsa-miR-3128, hsa-miR-152-3p, hsa-miR-4635,  hsa-miR-3689f, hsa-miR-6774-5p, hsa-miR-6744-5p, hsa-miR-885-3p, hsa-miR-659-3p,  hsa-miR-6830-5p, hsa-miR-4439, hsa-miR-4720-5p, hsa-miR-371b-5p, hsa-miR-4270,  hsa-miR-550a-3-5p, hsa-miR-98-5p, hsa-miR-6837-5p, hsa-miR-5088-5p, hsa-miR-635,  hsa-miR-6787-5p, hsa-miR-342-5p |
| **Non-small cell lung cancer** | BRAF, PRKCA, E2F1, ERBB2, SOS2, CDK4, E2F2, NRAS, STK4, PIK3CB, RXRG, TGFA, PIK3R5, EML4, RAF1, RARB, EGFR, KRAS, CDK6, AKT2, PLCG1, PIK3CD, PIK3R3, CCND1, E2F3, AKT1, PIK3R1, RB1, SOS1, MAPK3, CASP9, PLCG2, PRKCB, AKT3, PIK3CA, FOXO3, MAP2K1, PDPK1, RASSF1, RXRB, MAPK1, GRB2, EGF, PIK3R2 | hsa-miR-3613-3p, hsa-miR-29c-3p, hsa-miR-6830-5p, hsa-miR-664b-3p, hsa-miR-1208,  hsa-miR-4663, hsa-miR-4462, hsa-miR-98-5p, hsa-miR-152-3p, hsa-miR-6837-5p,  hsa-miR-330-5p, hsa-miR-548ac, hsa-miR-659-3p, hsa-miR-548x-3p, hsa-miR-4270,  hsa-miR-8084, hsa-miR-6886-5p, hsa-miR-3148, hsa-miR-548a-3p, hsa-miR-27a-3p,  hsa-miR-7-1-3p, hsa-miR-6744-5p, hsa-miR-8063, hsa-miR-5100, hsa-miR-7-5p,  hsa-miR-629-3p, hsa-miR-4518, hsa-miR-1468-3p, hsa-miR-6507-3p, hsa-miR-4635,  hsa-miR-6785-5p, hsa-miR-371b-5p, hsa-miR-6867-5p, hsa-miR-6774-5p, hsa-miR-34a-5p, hsa-miR-3194-3p, hsa-miR-885-3p, hsa-miR-6893-5p, hsa-miR-204-3p, hsa-miR-4446-3p, hsa-miR-6825-5p, hsa-miR-4668-5p, hsa-miR-4439, hsa-miR-16-2-3p, hsa-miR-195-3p,  hsa-miR-338-5p, hsa-miR-3128, hsa-miR-342-5p, hsa-miR-6813-5p, hsa-let-7f-1-3p,  hsa-miR-3613-5p |
| **Cholinergic synapse** | CAMK2D, FOS, PRKCA, CACNA1A, GNG13, ADCY1, ADCY5, CHRM5, KCNQ2, GNG11, CAMK4, ADCY7, NRAS, CACNA1F, ADCY2, PIK3CB, CREB5, KCNQ5, PIK3R5, GNG12, KCNQ3, BCL2, CHRM3, GNAI3, GNB1, KRAS, FYN, CREB3, CREB1, CAMK2A, AKT2, GNA11, PLCB1, GNB2, CACNA1B, PIK3CD, PIK3R3, JAK2, AKT1, PRKACG, PRKACA, GNG7, GNG2, ITPR1, PIK3R1, PRKX, MAPK3, CHRM2, KCNJ6, PRKCB, CHRNB2, KCNJ2, GNAQ, AKT3, CAMK2B, CREB3L2, GNAI2, PIK3CA, CREB3L1, GNAO1, ADCY8, MAP2K1, GNG4, ITPR3, CACNA1D, MAPK1, ITPR2, GNG5, ADCY9, ADCY4, CHRNA7, GNB4, KCNQ1, KCNJ3, KCNQ4, PLCB4, PLCB2, GNB5, GNAI1, PRKACB, SLC5A7, CHRM1, ACHE, ADCY6, PIK3R2 | hsa-miR-98-5p, hsa-miR-6837-5p, hsa-miR-330-5p, hsa-miR-6785-5p, hsa-miR-6825-5p,  hsa-miR-3613-3p, hsa-miR-3148, hsa-miR-664b-3p, hsa-miR-8084, hsa-miR-548ac,  hsa-miR-548x-3p, hsa-miR-6886-5p, hsa-miR-29c-3p, hsa-miR-34a-5p, hsa-miR-27a-3p,  hsa-miR-371b-5p, hsa-miR-8063, hsa-let-7f-1-3p, hsa-miR-1208, hsa-miR-574-5p,  hsa-miR-7-1-3p, hsa-miR-7-5p, hsa-miR-4423-3p, hsa-miR-4668-5p, hsa-miR-152-3p,  hsa-miR-548a-3p, hsa-miR-1468-3p, hsa-miR-635, hsa-miR-659-3p, hsa-miR-4635,  hsa-miR-338-5p, hsa-miR-6893-5p, hsa-miR-3175, hsa-miR-6867-5p, hsa-miR-6744-5p,  hsa-miR-4446-3p, hsa-miR-550a-3-5p, hsa-miR-3194-3p, hsa-miR-296-5p, hsa-miR-342-5p, hsa-miR-3689f, hsa-miR-629-3p, hsa-miR-885-3p, hsa-miR-204-3p, hsa-miR-4270,  hsa-miR-4448, hsa-miR-6830-5p, hsa-miR-16-2-3p, hsa-miR-195-3p, hsa-miR-6813-5p,  hsa-miR-6774-5p, hsa-miR-6787-5p, hsa-miR-3613-5p, hsa-miR-5088-5p, hsa-miR-3128,  hsa-miR-4720-5p, hsa-miR-4663 |
| **Glycosaminoglycan biosynthesis - heparan sulfate / heparin** | HS6ST2, EXT1, NDST1, EXTL3, HS2ST1, NDST2, HS3ST1, B3GAT3, EXT2, HS3ST5, HS3ST3B1, HS3ST3A1, HS3ST2, XYLT2, GLCE, NDST3, XYLT1, B4GALT7, RP11-574K11.31 | hsa-miR-3148, hsa-miR-7-5p, hsa-miR-338-5p, hsa-miR-98-5p, hsa-miR-664b-3p,  hsa-miR-548x-3p, hsa-miR-3613-5p, hsa-miR-3128, hsa-miR-3613-3p, hsa-miR-885-3p,  hsa-miR-29c-3p, hsa-miR-6785-5p, hsa-miR-4720-5p, hsa-miR-4270, hsa-miR-4668-5p,  hsa-miR-1468-3p, hsa-miR-34a-5p, hsa-miR-548ac, hsa-miR-1208, hsa-miR-4663,  hsa-miR-5189-3p, hsa-miR-6744-5p, hsa-miR-6893-5p, hsa-miR-548a-3p, hsa-miR-7-1-3p, hsa-miR-8063, hsa-miR-204-3p, hsa-miR-635, hsa-miR-550a-3-5p, hsa-miR-6774-5p,  hsa-let-7f-1-3p, hsa-miR-6507-3p, hsa-miR-16-2-3p, hsa-miR-195-3p, hsa-miR-1260b,  hsa-miR-371b-5p, hsa-miR-629-3p, hsa-miR-152-3p, hsa-miR-330-5p, hsa-miR-4635 |
| **Hepatitis B** | TLR2, FOS, PRKCA, STAT3, ATP6AP1, E2F1, TGFBR1, NFKB1, CDK4, DDX58, CXCL8, ATF2, E2F2, NRAS, PTK2B, IFNA8, CCNA2, PIK3CB, CREB5, PIK3R5, CDK2, BAX, IFNB1, RAF1, SMAD3, CHUK, BCL2, CDKN1B, BIRC5, YWHAB, MAP3K1, TLR4, KRAS, CDK6, STAT5B, DDX3X, TGFB1, CREB3, FASLG, YWHAQ, EGR3, HSPG2, FADD, NFATC4, IFNAR1, CREB1, IKBKB, APAF1, AKT2, MAVS, MYD88, MAPK9, CASP3, PIK3CD, PIK3R3, CCND1, SMAD4, CCNE2, E2F3, NFATC2, MAPK8, AKT1, STAT2, TIRAP, MYC, EGR2, TLR3, NFKBIA, PIK3R1, RB1, YWHAZ, TBK1, MAPK3, CASP9, PRKCB, FAS, TGFB2, EP300, CASP8, AKT3, CREB3L2, PIK3CA, CREB3L1, CDKN1A, MAP2K1, STAT1, CYCS, STAT6, MAP2K4, TNF, RELA, PTEN, NFATC3, MAPK1, CREBBP, NFATC1, GRB2, JAK1, ELK1, MAPK10, VDAC3, PIK3R2 | hsa-miR-3613-3p, hsa-miR-548ac, hsa-miR-548x-3p, hsa-miR-7-1-3p, hsa-miR-6744-5p,  hsa-miR-3148, hsa-miR-1468-3p, hsa-miR-330-5p, hsa-miR-6785-5p, hsa-miR-371b-5p,  hsa-miR-8063, hsa-miR-152-3p, hsa-miR-27a-3p, hsa-miR-548a-3p, hsa-miR-6837-5p,  hsa-miR-6825-5p, hsa-miR-5100, hsa-miR-4720-5p, hsa-miR-34a-5p, hsa-miR-6893-5p,  hsa-miR-7-5p, hsa-miR-629-3p, hsa-miR-664b-3p, hsa-miR-1208, hsa-miR-8084,  hsa-miR-6867-5p, hsa-miR-29c-3p, hsa-miR-16-2-3p, hsa-miR-195-3p, hsa-miR-885-3p,  hsa-let-7f-1-3p, hsa-miR-3613-5p, hsa-miR-4635, hsa-miR-338-5p, hsa-miR-4668-5p,  hsa-miR-98-5p, hsa-miR-659-3p, hsa-miR-4270, hsa-miR-3689f, hsa-miR-4446-3p,  hsa-miR-101-5p, hsa-miR-6830-5p, hsa-miR-4663, hsa-miR-6787-5p, hsa-miR-635,  hsa-miR-3194-3p, hsa-miR-6774-5p, hsa-miR-4462, hsa-miR-204-3p, hsa-miR-574-5p,  hsa-miR-4518, hsa-miR-3128, hsa-miR-550a-3-5p, hsa-miR-4448, hsa-miR-342-5p,  hsa-miR-3175, hsa-miR-5088-5p, hsa-miR-4439, hsa-miR-3609, hsa-miR-6813-5p,  hsa-miR-6886-5p |
| **cGMP-PKG signalling pathway** | IRS2, GUCY1B3, MYLK4, ATP1B2, ADCY1, ADCY5, VDAC1, ROCK1, ATF2, ADCY7, CACNA1F, ATP2B2, ADCY2, CALM3, CALM1, AGTR1, GUCY1A3, PIK3CB, SLC25A31, CREB5, ATP1A2, PIK3R5, PPP1CC, GNA13, ROCK2, BDKRB2, PDE3A, PPP3R1, RAF1, KCNMA1, MEF2D, ATP1B1, ADRB2, SLC8A2, SLC25A5, MYLK2, GNAI3, ADRB3, RHOA, CREB3, CALM2, PRKCE, ADRA2A, KCNMB1, ATP2B1, TRPC6, GATA4, NFATC4, SLC8A3, PDE3B, CREB1, ADORA1, PPP3CA, AKT2, GNA11, PLCB1, PRKG2, ATP2A1, PPP1R12A, PIK3CD, PIK3R3, PPP3CB, ATP1B3, PLN, GTF2IRD1, NFATC2, AKT1, EDNRB, SLC8A1, ITPR1, KCNMB2, PIK3R1, PDE2A, IRS1, INSR, ATP1B4, MAPK3, MRVI1, CNGA1, KCNMB4, GNAQ, AKT3, MYLK3, CREB3L2, GUCY1A2, GNAI2, PIK3CA, VASP, CREB3L1, PDE5A, ADCY8, MAP2K1, ATP2B4, CNGB1, EDNRA, ATP2A2, MEF2C, PRKG1, ATP2B3, ATP1A1, ITPR3, CACNA1D, NFATC3, ATP2A3, MAPK1, ITPR2, SRF, NFATC1, ADCY9, ADCY4, ADRA1D, MEF2A, ADRB1, PLCB4, PLCB2, MYLK, GNAI1, PPP3R2, PPP1CB, VDAC3, ADCY6, PIK3R2 | hsa-miR-3128, hsa-miR-3613-3p, hsa-miR-6785-5p, hsa-miR-548ac, hsa-miR-664b-3p,  hsa-miR-6867-5p, hsa-miR-548x-3p, hsa-miR-6507-3p, hsa-miR-4270, hsa-miR-7-5p,  hsa-miR-101-5p, hsa-miR-16-2-3p, hsa-miR-629-3p, hsa-miR-8063, hsa-miR-6893-5p,  hsa-miR-659-3p, hsa-miR-3148, hsa-miR-330-5p, hsa-miR-4446-3p, hsa-miR-335-5p,  hsa-miR-548a-3p, hsa-miR-27a-3p, hsa-miR-1208, hsa-miR-7-1-3p, hsa-miR-6744-5p,  hsa-miR-204-3p, hsa-miR-342-5p, hsa-miR-5088-5p, hsa-miR-8084, hsa-miR-885-3p,  hsa-miR-29c-3p, hsa-miR-98-5p, hsa-let-7f-1-3p, hsa-miR-195-3p, hsa-miR-338-5p,  hsa-miR-6825-5p, hsa-miR-3194-3p, hsa-miR-4668-5p, hsa-miR-1468-3p, hsa-miR-34a-5p, hsa-miR-4635, hsa-miR-371b-5p, hsa-miR-152-3p, hsa-miR-6830-5p, hsa-miR-5189-3p,  hsa-miR-4448, hsa-miR-550a-3-5p, hsa-miR-4750-5p, hsa-miR-4439, hsa-miR-635,  hsa-miR-6774-5p, hsa-miR-3609, hsa-miR-574-5p, hsa-miR-3613-5p, hsa-miR-4663,  hsa-miR-6837-5p, hsa-miR-296-5p, hsa-miR-3175, hsa-miR-5100, hsa-miR-3689f,  hsa-miR-6746-5p, hsa-miR-6886-5p, hsa-miR-6813-5p |
| **Calcium signalling pathway** | CAMK2D, PDE1C, PRKCA, PDGFRA, CACNA1A, BDKRB1, MYLK4, ERBB2, PTGFR, CACNA1G, ADCY1, VDAC1, CHRM5, CAMK4, PLCD1, ADCY7, ADORA2B, CACNA1F, ORAI2, PTK2B, ATP2B2, CD38, ADCY2, CALM3, CALM1, AGTR1, GNAS, SLC25A31, PLCD3, P2RX5, GRM5, BDKRB2, STIM2, PPP3R1, DRD1, CCKBR, CHRM3, ADRB2, SLC8A2, SLC25A5, EGFR, MYLK2, ADRB3, PLCZ1, ERBB3, HTR2B, CALM2, PTGER3, ATP2B1, ADORA2A, PDE1B, SLC8A3, CACNA1I, CAMK2A, PDE1A, PPP3CA, NOS1, GNA11, RYR1, PLCB1, ATP2A1, CACNA1B, PLCG1, CYSLTR2, PPP3CB, RYR2, PLN, F2R, GRM1, EDNRB, PRKACG, PRKACA, SLC8A1, CACNA1E, ITPR1, PRKX, P2RX1, STIM1, NTSR1, OXTR, NOS2, CHRM2, RYR3, PHKG2, CYSLTR1, PHKA1, PLCG2, DRD5, PRKCB, GNAL, HTR5A, GNAQ, TACR1, MYLK3, CAMK2B, PLCE1, GRIN2D, ADCY8, TACR3, LHCGR, ATP2B4, EDNRA, ATP2A2, HTR2C, PTAFR, GRIN1, ATP2B3, ITPR3, CACNA1D, HTR7, ATP2A3, PHKB, ITPR2, GRIN2A, ADCY9, ADCY4, CHRNA7, HRH2, ADRA1D, ADRB1, PLCB4, PLCB2, ORAI3, MYLK, ERBB4, PRKACB, PPP3R2, TBXA2R, HTR2A, VDAC3, CHRM1 | hsa-miR-3148, hsa-miR-27a-3p, hsa-miR-98-5p, hsa-miR-548x-3p, hsa-miR-4668-5p,  hsa-miR-1468-3p, hsa-miR-548ac, hsa-miR-664b-3p, hsa-miR-7-5p, hsa-miR-4518,  hsa-miR-3128, hsa-miR-6837-5p, hsa-miR-6785-5p, hsa-miR-574-5p, hsa-miR-8063,  hsa-miR-4750-5p, hsa-miR-6893-5p, hsa-miR-3613-3p, hsa-miR-8084, hsa-miR-885-3p,  hsa-miR-204-3p, hsa-miR-548a-3p, hsa-miR-330-5p, hsa-miR-34a-5p, hsa-miR-6825-5p,  hsa-miR-659-3p, hsa-let-7f-1-3p, hsa-miR-1208, hsa-miR-16-2-3p, hsa-miR-4635,  hsa-miR-629-3p, hsa-miR-7-1-3p, hsa-miR-371b-5p, hsa-miR-338-5p, hsa-miR-550a-3-5p, hsa-miR-5189-3p, hsa-miR-152-3p, hsa-miR-6867-5p, hsa-miR-4270, hsa-miR-4439,  hsa-miR-4423-3p, hsa-miR-5088-5p, hsa-miR-4446-3p, hsa-miR-3194-3p, hsa-miR-335-5p, hsa-miR-3175, hsa-miR-5100, hsa-miR-6744-5p, hsa-miR-6830-5p, hsa-miR-4720-5p,  hsa-miR-29c-3p, hsa-miR-3613-5p, hsa-miR-4663, hsa-miR-635, hsa-miR-296-5p,  hsa-miR-6774-5p, hsa-miR-195-3p, hsa-miR-3689f, hsa-miR-6746-5p, hsa-miR-101-5p,  hsa-miR-3605-3p |
| **Melanoma** | BRAF, FGF12, PDGFRA, E2F1, MET, CDK4, FGF14, E2F2, NRAS, FGF13, PIK3CB, PIK3R5, FGF10, RAF1, IGF1R, EGFR, FGF20, KRAS, CDK6, MITF, FGF11, AKT2, CDH1, PIK3CD, PIK3R3, CCND1, E2F3, AKT1, PDGFB, PIK3R1, RB1, FGF9, MAPK3, FGF2, FGF18, FGF5, IGF1, AKT3, PDGFC, PDGFD, PIK3CA, FGF8, CDKN1A, MAP2K1, FGF23, HGF, PTEN, FGFR1, FGF16, MAPK1, FGF1, FGF7, MDM2, EGF, PDGFA, PIK3R2 | hsa-miR-3613-3p, hsa-miR-6785-5p, hsa-miR-548ac, hsa-miR-664b-3p, hsa-miR-548x-3p, hsa-miR-4635, hsa-miR-371b-5p, hsa-miR-8063, hsa-miR-548a-3p, hsa-miR-34a-5p,  hsa-miR-3194-3p, hsa-miR-7-5p, hsa-miR-7-1-3p, hsa-miR-6744-5p, hsa-miR-3148,  hsa-miR-152-3p, hsa-miR-29c-3p, hsa-miR-6867-5p, hsa-miR-16-2-3p, hsa-miR-195-3p,  hsa-miR-885-3p, hsa-miR-27a-3p, hsa-let-7f-1-3p, hsa-miR-3613-5p, hsa-miR-629-3p,  hsa-miR-338-5p, hsa-miR-3128, hsa-miR-98-5p, hsa-miR-4668-5p, hsa-miR-330-5p,  hsa-miR-1208, hsa-miR-635, hsa-miR-4270, hsa-miR-6774-5p, hsa-miR-659-3p,  hsa-miR-574-5p, hsa-miR-6837-5p, hsa-miR-6830-5p, hsa-miR-1468-3p, hsa-miR-3609,  hsa-miR-342-5p, hsa-miR-5189-3p, hsa-miR-4439, hsa-miR-8084, hsa-miR-550a-3-5p,  hsa-miR-204-3p, hsa-miR-6813-5p, hsa-miR-4446-3p, hsa-miR-6825-5p, hsa-miR-6893-5p, hsa-miR-6886-5p, hsa-miR-3689f |
| **HIF-1 signalling pathway** | CAMK2D, PRKCA, STAT3, ERBB2, NFKB1, PIK3CB, CUL2, IFNGR2, PIK3R5, ARNT, ANGPT2, BCL2, CDKN1B, IGF1R, EGFR, TLR4, IFNGR1, TCEB1, VHL, HK2, RPS6KB2, CAMK2A, AKT2, PDHA1, NOX1, PLCG1, PIK3CD, PIK3R3, EIF4E, HIF1A, AKT1, TFRC, HMOX1, FLT1, EGLN3, PDK1, PIK3R1, LDHA, NOS2, INSR, MAPK3, CYBB, PLCG2, EGLN2, PRKCB, IGF1, EP300, AKT3, CAMK2B, EIF4E2, ANGPT1, PIK3CA, RBX1, IFNG, ENO3, CDKN1A, MAP2K1, SLC2A1, MKNK1, PFKFB2, EDN1, MKNK2, MTOR, RELA, VEGFA, MAPK1, CREBBP, SERPINE1, PFKFB4, TEK, PGK1, ENO2, EGF, RPS6KB1, EGLN1, PFKFB3, TIMP1, IL6R, PIK3R2 | hsa-miR-4270, hsa-miR-338-5p, hsa-miR-34a-5p, hsa-miR-3194-3p, hsa-miR-3613-3p,  hsa-miR-3148, hsa-miR-548a-3p, hsa-miR-548ac, hsa-miR-664b-3p, hsa-miR-27a-3p,  hsa-miR-548x-3p, hsa-miR-3613-5p, hsa-miR-1208, hsa-miR-7-5p, hsa-miR-195-3p,  hsa-miR-371b-5p, hsa-miR-8063, hsa-miR-6893-5p, hsa-miR-3689f, hsa-miR-1468-3p,  hsa-miR-4635, hsa-miR-16-2-3p, hsa-miR-7-1-3p, hsa-miR-6867-5p, hsa-miR-5100,  hsa-miR-6774-5p, hsa-miR-29c-3p, hsa-miR-8084, hsa-miR-6886-5p, hsa-miR-4668-5p,  hsa-miR-4448, hsa-miR-4446-3p, hsa-miR-152-3p, hsa-miR-6825-5p, hsa-miR-574-5p,  hsa-miR-629-3p, hsa-miR-330-5p, hsa-miR-6785-5p, hsa-miR-3128, hsa-let-7f-1-3p,  hsa-miR-98-5p, hsa-miR-4439, hsa-miR-885-3p, hsa-miR-5189-3p, hsa-miR-6830-5p,  hsa-miR-659-3p, hsa-miR-204-3p, hsa-miR-6813-5p, hsa-miR-6744-5p, hsa-miR-3175,  hsa-miR-6837-5p, hsa-miR-3609, hsa-miR-4720-5p, hsa-miR-4663, hsa-miR-342-5p |
| **Hypertrophic cardiomyopathy (HCM)** | ACTB, CACNG8, DES, CACNA2D3, ITGB1, ITGB8, TPM1, ITGA9, PRKAA2, CACNA1F, ITGA8, CACNB4, CACNG7, ACTG1, TTN, ITGB6, SGCD, ITGA5, ITGA3, ACE, ITGA1, TGFB1, TPM3, TPM4, ITGAV, ITGA11, DMD, ACTC1, RYR2, PRKAG1, CACNB1, SLC8A1, DAG1, CACNG4, ITGA2, ITGA10, PRKAA1, CACNA2D4, CACNA2D2, CACNG2, SGCA, PRKAB2, IGF1, TGFB2, ITGA7, PRKAG2, PRKAB1, LMNA, LAMA2, ITGA4, CACNG6, CACNA2D1, ATP2A2, CACNB2, ITGA6, TNF, CACNA1D, SGCB, ITGA2B, PRKAG3, TPM2, CACNB3, ITGB3 | hsa-miR-152-3p, hsa-miR-6867-5p, hsa-miR-574-5p, hsa-miR-3613-3p, hsa-miR-3128,  hsa-miR-330-5p, hsa-miR-27a-3p, hsa-miR-34a-5p, hsa-miR-4663, hsa-miR-4270,  hsa-miR-629-3p, hsa-miR-98-5p, hsa-miR-29c-3p, hsa-miR-6837-5p, hsa-miR-548a-3p,  hsa-miR-1468-3p, hsa-miR-664b-3p, hsa-miR-550a-3-5p, hsa-miR-548x-3p, hsa-miR-16-2-3p, hsa-miR-195-3p, hsa-miR-338-5p, hsa-miR-3148, hsa-miR-548ac, hsa-miR-3613-5p,  hsa-miR-3194-3p, hsa-miR-6825-5p, hsa-miR-635, hsa-miR-6774-5p, hsa-miR-6893-5p,  hsa-miR-659-3p, hsa-miR-7-5p, hsa-miR-7-1-3p, hsa-miR-101-5p, hsa-miR-204-3p,  hsa-miR-296-5p, hsa-miR-8063, hsa-miR-371b-5p, hsa-miR-3175, hsa-let-7f-1-3p,  hsa-miR-6744-5p, hsa-miR-6785-5p, hsa-miR-4668-5p, hsa-miR-4635, hsa-miR-342-5p,  hsa-miR-1208, hsa-miR-5088-5p, hsa-miR-8084, hsa-miR-885-3p, hsa-miR-4720-5p,  hsa-miR-3201 |
| **Shigellosis** | ARPC5, PFN1, ACTB, ARPC5L, NFKB1, WASF1, ITGB1, ROCK1, WASL, RIPK2, CXCL8, CRKL, BTRC, CRK, MAPK14, ACTG1, ROCK2, NOD2, ITGA5, CHUK, ELMO2, NOD1, VCL, ARPC1B, CTTN, IKBKB, HCLS1, MAPK9, PFN2, DOCK1, U2AF1L4, MAPK8, DIAPH1, ARPC2, WASF2, NFKBIA, ARPC1A, RAC1, MAPK3, CDC42, ELMO1, UBE2D2, ATG5, RELA, MAPK1, FBXW11, ABL1, RHOG, MAPK10, CD44 | hsa-miR-3128, hsa-miR-548x-3p, hsa-miR-4635, hsa-miR-4446-3p, hsa-miR-7-5p,  hsa-miR-4270, hsa-miR-3613-3p, hsa-miR-152-3p, hsa-miR-6785-5p, hsa-miR-34a-5p,  hsa-miR-548ac, hsa-miR-664b-3p, hsa-miR-659-3p, hsa-let-7f-1-3p, hsa-miR-3689f,  hsa-miR-29c-3p, hsa-miR-550a-3-5p, hsa-miR-338-5p, hsa-miR-98-5p, hsa-miR-6813-5p,  hsa-miR-7-1-3p, hsa-miR-8063, hsa-miR-196b-3p, hsa-miR-330-5p, hsa-miR-3148,  hsa-miR-574-5p, hsa-miR-4668-5p, hsa-miR-6825-5p, hsa-miR-27a-3p, hsa-miR-6886-5p, hsa-miR-1468-3p, hsa-miR-342-5p, hsa-miR-548a-3p, hsa-miR-3613-5p, hsa-miR-4423-3p, hsa-miR-371b-5p, hsa-miR-8084, hsa-miR-885-3p, hsa-miR-6837-5p, hsa-miR-635,  hsa-miR-3194-3p, hsa-miR-6774-5p, hsa-miR-4448, hsa-miR-3175, hsa-miR-5088-5p,  hsa-miR-6893-5p, hsa-miR-204-3p, hsa-miR-1208, hsa-miR-6867-5p, hsa-miR-4663,  hsa-miR-3609, hsa-miR-4439 |
| **Cocaine addiction** | GRIN3A, NFKB1, ADCY5, ATF2, GNAS, CREB5, DRD1, SLC18A2, GRM3, GNAI3, CDK5R1, BDNF, CREB3, GRM2, CREB1, GRIA2, MAOB, DRD2, DLG4, MAOA, FOSB, PDYN, PRKACG, PRKACA, RGS9, PRKX, CREB3L2, GNAI2, GRIN2D, CREB3L1, PPP1R1B, GRIN1, RELA, GRIN2A, GNAI1, PRKACB, GRIN2B | hsa-miR-3148, hsa-miR-6867-5p, hsa-miR-371b-5p, hsa-miR-4663, hsa-miR-338-5p,  hsa-miR-3613-3p, hsa-miR-548ac, hsa-let-7f-1-3p, hsa-miR-548x-3p, hsa-miR-5100,  hsa-miR-8063, hsa-miR-6744-5p, hsa-miR-659-3p, hsa-miR-7-1-3p, hsa-miR-664b-3p,  hsa-miR-574-5p, hsa-miR-3201, hsa-miR-8084, hsa-miR-4668-5p, hsa-miR-152-3p,  hsa-miR-548a-3p, hsa-miR-1468-3p, hsa-miR-330-5p, hsa-miR-6785-5p, hsa-miR-4446-3p, hsa-miR-27a-3p, hsa-miR-1208, hsa-miR-3194-3p, hsa-miR-4270, hsa-miR-5189-3p,  hsa-miR-7-5p, hsa-miR-16-2-3p, hsa-miR-195-3p, hsa-miR-629-3p, hsa-miR-4423-3p,  hsa-miR-6893-5p, hsa-miR-6825-5p, hsa-miR-4635, hsa-miR-3175, hsa-miR-4720-5p,  hsa-miR-29c-3p, hsa-miR-3689f, hsa-miR-5088-5p, hsa-miR-6830-5p, hsa-miR-3128,  hsa-miR-3613-5p |
| **Wnt signalling pathway** | FZD7, CTNNBIP1, CAMK2D, DAAM2, GSK3B, PRKCA, SFRP4, DVL3, CSNK2A2, FZD5, LRP6, TBL1X, WNT7A, BTRC, APC, VANGL1, WNT10B, TCF7L2, WNT5A, CTBP1, CHD8, SOX17, PORCN, CCND2, DKK2, ROCK2, TCF7L1, FZD6, PPP3R1, CTBP2, SMAD3, WNT2B, CUL1, WNT4, WNT3, LRP5, CSNK1A1L, RHOA, FZD8, FZD3, SKP1, NFATC4, FRAT2, CAMK2A, PPP3CA, PRICKLE1, NLK, PLCB1, FZD4, SENP2, SOST, MAPK9, GPC4, CCND1, SMAD4, CTNNB1, PPP3CB, AXIN2, NFATC2, MAPK8, NKD1, CSNK1A1, MYC, PRKACG, PRKACA, VANGL2, CSNK2B, CSNK2A1, PRKX, RAC1, SIAH1, PSEN1, FZD1, PRKCB, PRICKLE2, CSNK1E, EP300, WNT10A, CAMK2B, CXXC4, RBX1, LEF1, SFRP1, WNT2, WIF1, WNT9B, FOSL1, TCF7, NFATC3, DAAM1, MAP3K7, CREBBP, FBXW11, NFATC1, WNT7B, DVL2, TBL1XR1, PPARD, WNT9A, PLCB4, PLCB2, CCND3, MAPK10, PRKACB, PPP3R2 | hsa-miR-3613-3p, hsa-miR-548a-3p, hsa-miR-27a-3p, hsa-miR-548x-3p, hsa-miR-7-1-3p,  hsa-miR-4668-5p, hsa-miR-3148, hsa-miR-34a-5p, hsa-miR-548ac, hsa-let-7f-1-3p,  hsa-miR-664b-3p, hsa-miR-1208, hsa-miR-8063, hsa-miR-6825-5p, hsa-miR-6893-5p,  hsa-miR-6867-5p, hsa-miR-7-5p, hsa-miR-4635, hsa-miR-152-3p, hsa-miR-4448,  hsa-miR-330-5p, hsa-miR-338-5p, hsa-miR-4439, hsa-miR-1468-3p, hsa-miR-6785-5p,  hsa-miR-371b-5p, hsa-miR-3175, hsa-miR-16-2-3p, hsa-miR-195-3p, hsa-miR-204-3p,  hsa-miR-98-5p, hsa-miR-659-3p, hsa-miR-4270, hsa-miR-5189-3p, hsa-miR-29c-3p,  hsa-miR-4446-3p, hsa-miR-3613-5p, hsa-miR-629-3p, hsa-miR-6744-5p, hsa-miR-5088-5p, hsa-miR-574-5p, hsa-miR-296-5p, hsa-miR-6830-5p, hsa-miR-4423-3p, hsa-miR-4720-5p, hsa-miR-4518, hsa-miR-4462, hsa-miR-3194-3p, hsa-miR-8084, hsa-miR-635,  hsa-miR-6774-5p, hsa-miR-3128, hsa-miR-4663, hsa-miR-6837-5p, hsa-miR-885-3p,  hsa-miR-3689f, hsa-miR-3609, hsa-miR-550a-3-5p, hsa-miR-342-5p, hsa-miR-1260b,  hsa-miR-6507-3p, hsa-miR-6787-5p |
| **Caffeine metabolism** | NAT1, CYP1A2, CYP2A6, NAT2, XDH | hsa-miR-664b-3p, hsa-miR-8063, hsa-miR-3605-3p, hsa-miR-3613-3p, hsa-miR-6785-5p,  hsa-miR-548ac, hsa-miR-29c-3p, hsa-miR-338-5p, hsa-miR-16-2-3p, hsa-miR-195-3p |
| **Dopaminergic synapse** | CAMK2D, FOS, GSK3B, PRKCA, CACNA1A, GNG13, PPP2R5E, KIF5A, ADCY5, ATF2, GNG11, PPP2R3A, CALM3, CALM1, GNAS, PPP2R2C, PPP2CA, CREB5, PPP1CC, MAPK14, GNG12, DRD1, SLC18A2, GRIA1, PPP2R5D, PPP2R2B, GNAI3, GNB1, PPP2R2D, PPP2R5C, CREB3, CALM2, PPP2R5A, CREB1, GRIA2, CAMK2A, PPP3CA, AKT2, ARNTL, MAOB, PLCB1, PPP2R5B, MAPK9, DRD2, GNB2, CACNA1B, PPP3CB, MAOA, MAPK8, AKT1, PPP2R2A, SCN1A, PPP2R1A, PRKACG, PRKACA, GNG7, GNG2, ITPR1, PRKX, PPP2R3C, KCNJ6, DRD5, PRKCB, GNAL, GNAQ, AKT3, CAMK2B, CREB3L2, GRIA4, GNAI2, CREB3L1, GNAO1, KCNJ9, GNG4, PPP1R1B, ITPR3, CACNA1D, ITPR2, PPP2R1B, GNG5, GRIN2A, CALY, GNB4, KIF5C, KCNJ5, KCNJ3, PLCB4, PLCB2, GNB5, CLOCK, KIF5B, MAPK10, GNAI1, PRKACB, PPP1CB, GRIA3, GRIN2B | hsa-miR-3613-3p, hsa-miR-548a-3p, hsa-miR-664b-3p, hsa-miR-3613-5p, hsa-miR-8063,  hsa-miR-3148, hsa-miR-548ac, hsa-miR-27a-3p, hsa-miR-548x-3p, hsa-miR-7-1-3p,  hsa-miR-6893-5p, hsa-let-7f-1-3p, hsa-miR-371b-5p, hsa-miR-635, hsa-miR-3128,  hsa-miR-6837-5p, hsa-miR-1468-3p, hsa-miR-330-5p, hsa-miR-204-3p, hsa-miR-3175,  hsa-miR-550a-3-5p, hsa-miR-4423-3p, hsa-miR-6867-5p, hsa-miR-3689f, hsa-miR-6787-5p, hsa-miR-4635, hsa-miR-629-3p, hsa-miR-8084, hsa-miR-7-5p, hsa-miR-34a-5p,  hsa-miR-29c-3p, hsa-miR-4663, hsa-miR-338-5p, hsa-miR-4668-5p, hsa-miR-3194-3p,  hsa-miR-6830-5p, hsa-miR-4270, hsa-miR-16-2-3p, hsa-miR-195-3p, hsa-miR-5088-5p,  hsa-miR-152-3p, hsa-miR-6785-5p, hsa-miR-659-3p, hsa-miR-574-5p, hsa-miR-6825-5p,  hsa-miR-4462, hsa-miR-4518, hsa-miR-3201, hsa-miR-4446-3p, hsa-miR-1208,  hsa-miR-5189-3p, hsa-miR-5100, hsa-miR-6744-5p, hsa-miR-6813-5p, hsa-miR-3605-3p,  hsa-miR-101-5p, hsa-miR-4720-5p, hsa-miR-3609, hsa-miR-6886-5p, hsa-miR-98-5p,  hsa-miR-335-5p, hsa-miR-342-5p, hsa-miR-4439, hsa-miR-4448, hsa-miR-885-3p |
| **Thyroid hormone synthesis** | PRKCA, ATP1B2, ADCY1, ADCY5, ATF2, TTF2, ADCY7, ADCY2, SLC26A4, GNAS, CREB5, ATP1A2, TPO, HSPA5, ATP1B1, GPX7, CREB3, PAX8, CANX, GSR, CREB1, PLCB1, LRP2, ATP1B3, GPX3, DUOX2, TSHR, PRKACG, PRKACA, ITPR1, HSP90B1, PRKX, ATP1B4, GPX8, PRKCB, GNAQ, CREB3L2, CREB3L1, ADCY8, TG, IYD, SLC5A5, ATP1A1, ITPR3, ITPR2, ADCY9, ADCY4, GPX5, PLCB4, PLCB2, PRKACB, ADCY6 | hsa-miR-3613-3p, hsa-miR-8084, hsa-miR-3148, hsa-miR-7-5p, hsa-miR-6830-5p,  hsa-miR-548a-3p, hsa-miR-548ac, hsa-miR-664b-3p, hsa-miR-27a-3p, hsa-let-7f-1-3p,  hsa-miR-548x-3p, hsa-miR-4270, hsa-miR-16-2-3p, hsa-miR-195-3p, hsa-miR-4635,  hsa-miR-7-1-3p, hsa-miR-371b-5p, hsa-miR-338-5p, hsa-miR-8063, hsa-miR-6893-5p,  hsa-miR-4663, hsa-miR-29c-3p, hsa-miR-629-3p, hsa-miR-6825-5p, hsa-miR-3689f,  hsa-miR-204-3p, hsa-miR-4668-5p, hsa-miR-1468-3p, hsa-miR-34a-5p, hsa-miR-5088-5p, hsa-miR-152-3p, hsa-miR-885-3p, hsa-miR-574-5p, hsa-miR-6785-5p, hsa-miR-342-5p,  hsa-miR-1208, hsa-miR-635, hsa-miR-6774-5p, hsa-miR-550a-3-5p, hsa-miR-4423-3p,  hsa-miR-4446-3p, hsa-miR-3194-3p, hsa-miR-4439, hsa-miR-330-5p, hsa-miR-3128,  hsa-miR-6867-5p, hsa-miR-3613-5p, hsa-miR-6744-5p, hsa-miR-296-5p, hsa-miR-3175 |
